# Genome-wide association studies reveal novel QTLs, QTL-by-environment interactions and their candidate genes for tocopherol content in soybean seed

**DOI:** 10.3389/fpls.2022.1026581

**Published:** 2022-10-27

**Authors:** Kuanwei Yu, Huanran Miao, Hongliang Liu, Jinghang Zhou, Meinan Sui, Yuhang Zhan, Ning Xia, Xue Zhao, Yingpeng Han

**Affiliations:** Key Laboratory of Soybean Biology in Chinese Ministry of Education (Key Laboratory of Soybean Biology and Breeding/Genetics of Chinese Agriculture Ministry), Northeast Agricultural University, Harbin, China

**Keywords:** GWAS, 3VmrMLM, soybean, tocopherol content, QTL, candidate genes

## Abstract

Genome-wide association studies (GWAS) is an efficient method to detect quantitative trait locus (QTL), and has dissected many complex traits in soybean [*Glycine max* (L.) Merr.]. Although these results have undoubtedly played a far-reaching role in the study of soybean biology, environmental interactions for complex traits in traditional GWAS models are frequently overlooked. Recently, a new GWAS model, 3VmrMLM, was established to identify QTLs and QTL-by-environment interactions (QEIs) for complex traits. In this study, the GLM, MLM, CMLM, FarmCPU, BLINK, and 3VmrMLM models were used to identify QTLs and QEIs for tocopherol (Toc) content in soybean seed, including δ‐Tocotrienol (δ‐Toc) content, γ‐Tocotrienol (γ‐Toc) content, α‐Tocopherol (α‐Toc) content, and total Tocopherol (T-Toc) content. As a result, 101 QTLs were detected by the above methods in single-environment analysis, and 57 QTLs and 13 QEIs were detected by 3VmrMLM in multi-environment analysis. Among these QTLs, some QTLs (Group I) were repeatedly detected three times or by at least two models, and some QTLs (Group II) were repeatedly detected only by 3VmrMLM. In the two Groups, 3VmrMLM was able to correctly detect all known QTLs in group I, while good results were achieved in Group II, for example, 8 novel QTLs were detected in Group II. In addition, comparative genomic analysis revealed that the proportion of *Glyma_max* specific genes near QEIs was higher, in other words, these QEIs nearby genes are more susceptible to environmental influences. Finally, around the 8 novel QTLs, 11 important candidate genes were identified using haplotype, and validated by RNA-Seq data and qRT-PCR analysis. In summary, we used phenotypic data of Toc content in soybean, and tested the accuracy and reliability of 3VmrMLM, and then revealed novel QTLs, QEIs and candidate genes for these traits. Hence, the 3VmrMLM model has broad prospects and potential for analyzing the genetic structure of complex quantitative traits in soybean.

## Introduction

Soybean [*Glycine max* (L.) Merr.] is an important crop, and provided a great source of protein, oil, vitamin, and other nutrients for humans around the world. As one of the functional nutrients of soybean, tocopherol (Toc) has strong antioxidative capabilities and benefits to human health. It can scavenge free radicals in the body and increase immune function (Meagher et al., 2001; Kumar et al., 2009). According to the chemical structure, Tocs are composed of four members: α-tocopherol (α-Toc), β-tocopherol (β-Toc), γ-tocopherol (γ-Toc), and δ-tocopherol (δ-Toc) (Wan et al., 2008; Rozanowska et al., 2019; Barouh et al., 2022). Among them, α-Toc has the highest activity (Shaw et al., 2016). Edible oil is one of the main sources of Toc (Packer and Fuchs, 1993). As the most widely produced vegetable oil in the world, soybean oil has the highest total-Toc content, however, γ-Toc in soybean oil accounts for more than 70%. Although γ-Toc has antioxidant and other physiological activities, α-Toc is more excellent (Bramley et al., 2000). Hence, elevating the α-Toc content and total-Toc content in soybean genetics is important for quality improvement.

The Toc content of soybean seed is a typical quantitative trait, and it is difficult to breed this target trait of soybean variety using traditional breeding. This requires a lengthy selection process (Britz et al., 2008; Seguin et al., 2010). As an ancient tetraploid plant (Blanc and Wolfe, 2004), the soybean owing to its large and complex genome background brings great challenges and difficulties in genetic improvement (Young and Bharti, 2012; Tian et al., 2020; Lemay et al., 2022).

Genome-wide association studies (GWAS) is a powerful genomics tool, and it can base on natural populations to detect quantitative trait locus (QTL) underlying complex quantitative traits (Burton et al., 2007; Hamblin et al., 2011). GWAS has the advantage of high-resolution and high-throughput, thus, this method for analysis provides great convenience for the study of genetic variation in soybean (Anderson et al., 2020). Since the first GWAS conducted in soybean until now, almost all the important agronomic traits have been covered and dissected (Zhou et al., 2015; Fang et al., 2017). And yet, different GWAS models yield different GWAS results when we owe high-quality genotype and phenotype data (Chatterjee et al., 2013). Therefore, selecting the most suitable model for GWAS analysis can increase the accuracy to identify QTLs.

The general linear model (GLM) (Price et al., 2006), the mixed linear model (MLM) (Yu et al., 2006), and the compressed mixed linear model (CMLM) (Zhang et al., 2010) are single-marker genome-wide scan models, and these models can comprise a one-dimensional genome scan by testing one marker at a time. Among them, CMLM is frequently used in the genomic dissection of soybean quantitative traits (Jing et al., 2018; Zhao et al., 2019; Sui et al., 2020). However, single-marker genome-wide scan models require Bonferroni correction and multiple tests (Wang et al., 2016). Bonferroni correction is a stringent criterion, although greatly reduced false positive rates, many important loci associated with the target traits were missed (Zhang et al., 2019). With the rapid development of statistical methods, several multi-locus GWAS approaches have been developed to improve the power of QTL detection (Segura et al., 2012; Wen et al., 2018). Such as the Bayesian-information and linkage disequilibrium iteratively nested keyway (BLINK) (Huang et al., 2018), and the fixed and random model circulating probability unification (FarmCPU) (Liu et al., 2016). The obvious advantage of these methods is not a Bonferroni correction, they can reduce the amount of calculation and improve the accuracy.

Recently, a novel model was presented, named 3V multi-locus random-SNP-effect mixed linear model (3VmrMLM) (Li et al., 2022a). It is a multi-marker genome-wide scan model, this model not only provides high QTL detection power and sensitivity, at the same time, but it can also detect the QTL-by-environment interaction (QEI) and the QTL-by-QTL interaction (QQI). In this study, based on 23,149 SNPs and 175 soybean germplasms, we used six models (including 3VmrMLM, BLINK, FarmCPU, GLM, MLM, and CMLM) and conducted GWAS of individual and total-Toc content across three environments. The aim of this study is to reveal novel QTLs and QEIs of soybean Toc content and screen candidate genes.

## Materials and methods

### Plant materials, field trials, and phenotypic evaluation

The material used in this study included 175 diverse soybean accessions (**Table S1**), which encompassed most of the northeast regions of China and other countries. These materials were collected from the Chinese National Soybean GeneBank (CNSGB) and can represent the genetic diversity inside and outside of China. In this study, all experimental materials were planted at Harbin (117°17′E, 33°18′N), Liaoning (41°48′N, 123°25′E), and, Jilin (124°82′E, 43°50′N) in 2021. The field trials used a single-row plot (3 m-long rows and spaced 0.65 m) and were arranged in a randomized complete block design with three replicates per test environment. After full maturity, mature kernels of 10 randomly selected plants in each line were collected and used for evaluation of individual and total Toc content. The soybean seed Toc extraction and measurement were performed according to previous reports (Ujiie et al., 2005).

### DNA isolation and sequencing

The genomic DNA of each sample from 175 tested accessions was isolated from young leaf was isolated by the method of CTAB (Han et al., 2015), and simplified-sequenced *via* specific locus amplified fragment sequencing (SLAF-seq) (Sun et al., 2013). The digest enzyme group of *Mse*I (EC: 3.1.21.4) and *Hae*III (EC: 3.1.21.4) (Thermo Fisher Scientific Inc, Waltham, MA, USA.) were used to obtain more than 50,000 sequencing tags, each 300-500 bp in length. The obtained markers were evenly distributed in unique genomic regions of the 20 soybean chromosomes. The short oligonucleotide alignment program 2 software (SOAP2) was used to align the raw paired-end reads to the soybean reference genome. Based on over 58,000 high-quality SLAF labels from each test sample, raw reads from the same genomic location were used to define SLAF groups. Genotypes were considered heterozygous if the minor allele depth or total allele depth of the sample was greater than 1/3 (Han et al., 2016).

### Population structure evaluation and linkage disequilibrium analysis

The principle component analysis (PCA) was performed using the genome association and prediction integrated tool (GAPIT) R package to analyze the population structure of the natural panel (Lipka et al., 2012). The linkage disequilibrium (LD) parameter (r^2^) for estimating the degree of LD between pair-wise SNPs (MAF ≥ 0.05 and missing data ≤ 10%) was calculated by TASSEL 5.0 (Bradbury et al., 2007). Unlike GWAS, missing SNP genotypes were not classified as major alleles prior to LD analysis. Parameters in the program included MAF (≥ 0.05) and completeness (> 80%) for each SNP.

### Genome-wide association studies

In total, 23,149 polymorphic SNP markers and 175 tested accessions were used to perform GWAS, it was performed using six models, including three single-locus model: MLM, GLM, CMLM, and three multi-locus models: FarmCPU, BLINK, 3VmrMLM. Among these, the GLM, MLM, CMLM, FarmCPU, and BLINK models were implemented with the R package “GAPIT” and visualization used scripts from the R package “qqman” (https://cran.r-project.org/package=qqman) and “CMplot “ (https://github.com/YinLiLin/R-CMplot).

The significant threshold value for the association between SNP and traits were determined by -log10 (P) ≥ 4, which is equivalent to P ≤ 0.0001, for MLM, GLM, CMLM, FarmCPU, and BLINK. The R software IIIVmrMLM (Li et al., 2022b) of the 3VmrMLM method (Li et al., 2022a) was downloaded from GitHub website (https://github.com/YuanmingZhang65/IIIVmrMLM). In this study, we used the single environment and multiple-environment methods to identify QTLs and QEIs. The significant threshold value was determined by LOD score ≥ 4.

### Prediction of candidate genes

Candidate genes located in the 200-kb genomic region (100 kb upstream and 100 kb downstream) of each significant or suggested QTL then identified and annotated the candidate genes with the soybean reference genome (Wm82.a2.v1, http://www.soybase.org) (Cheng et al., 2017). The gene ontology (GO) enrichment analysis of candidate genes using the online tool (https://www.soybase.org/goslimgraphic_v2/dashboard.php). In addition, the whole genome and QEIs candidate genes among soybean relatives were compared using OrthoVenn2 (https://orthovenn2.bioinfotoolkits.net/task/create) (Xu et al., 2019).

### Association analysis of candidate genes

Genome resequencing data were used to select the SNP variations within candidate genes. These SNP were located in exonic, intronic regions, upstream and downstream regions. Then, we combined the phenotype values of 56 soybean germplasms in three environments, these soybean germplasms were selected from the 175 diverse soybean accessions (**Table S1**) (including 9 high and low individual and total Toc germplasms), using the general linear model (GLM) in TASSEL 5.0 to identify SNPs of candidate genes that related to individual or total Toc content (Bradbury et al., 2007). Significant SNPs associated with the target trait were claimed when the test statistic was *P* < 0.01.

### Haplotype analysis

The haplotypes were classified based on all of the SNPs with an MAF >0.05 in each candidate gene. Best linear unbiased predictors (BLUP) value were calculated using the “Phenotype” (https://cran.r-project.org/package=Phenotype) in R package. For each Toc component, haplotypes containing 18 soybean germplasms accessions were used for comparative analysis. One-way ANOVA and Two-tailed unpaired t -test were used to compare the differences in TC-BLUP value among the haplotypes. Finally, we compared the individual or total Toc content among these different haplotypes.

### RNA-Seq data analysis of candidate genes

For candidate genes expression pattern analysis, first, we performed a differential expression pattern analysis at different tissues by downloading the RNA expression data from the plant public RNA seq database (PPRD) (http://ipf.sustech.edu.cn/pub/soybean/), which integrated all publicly available RNA-Seq soybean libraries (4,085) (Yu et al., 2022). Then, we also analyzed the expression of candidate genes in the development stage (R6) at different germplasms using the transcriptome data (unpublished data) from our laboratory. Additionally, we constructed a heat-map plot, and it was performed using the R package pheatmap (Kolde, 2012).

### Quantitative real−time PCR (qRT−PCR)

Total RNA was isolated using the RNAprep pure Plant Kit (DP432, Tiangen). First-strand cDNA was synthesized from total RNA using TIANScript RT kits (KR104, Tiangen). And qRT-PCRs were performed using SYBR Green (FP205, Tiangen) reagents on an ABI 7500 fast real-time PCR platform. All qRT-PCRs were performed in three independent repeats, and the relative levels of transcript abundance were calculated using the 2^−ΔΔCT^ method (Livak and Schmittgen 2001). The GmActin4 (*Glyma.12G063400*) was used as an internal control for data normalization. Primer sequences for candidate genes were obtained from the qPrimerDB database (**Table S2**) (Lu et al., 2018).

### Statistical analysis

Descriptive statistical analysis of phenotypic data including mean, minimum, maximum, coefficient of variation (CV), heritability, skewness, and kurtosis was performed using IBM SPSS statistics 25.0 (SPSS, Chicago, USA). One-way ANOVA with Dunnett’s multiple comparisons test and unpaired two-tailed t-test were performed using GraphPad Prism 9.4.1.

## Results

### Statistical and variation analysis of Toc content

**Figure 1:**
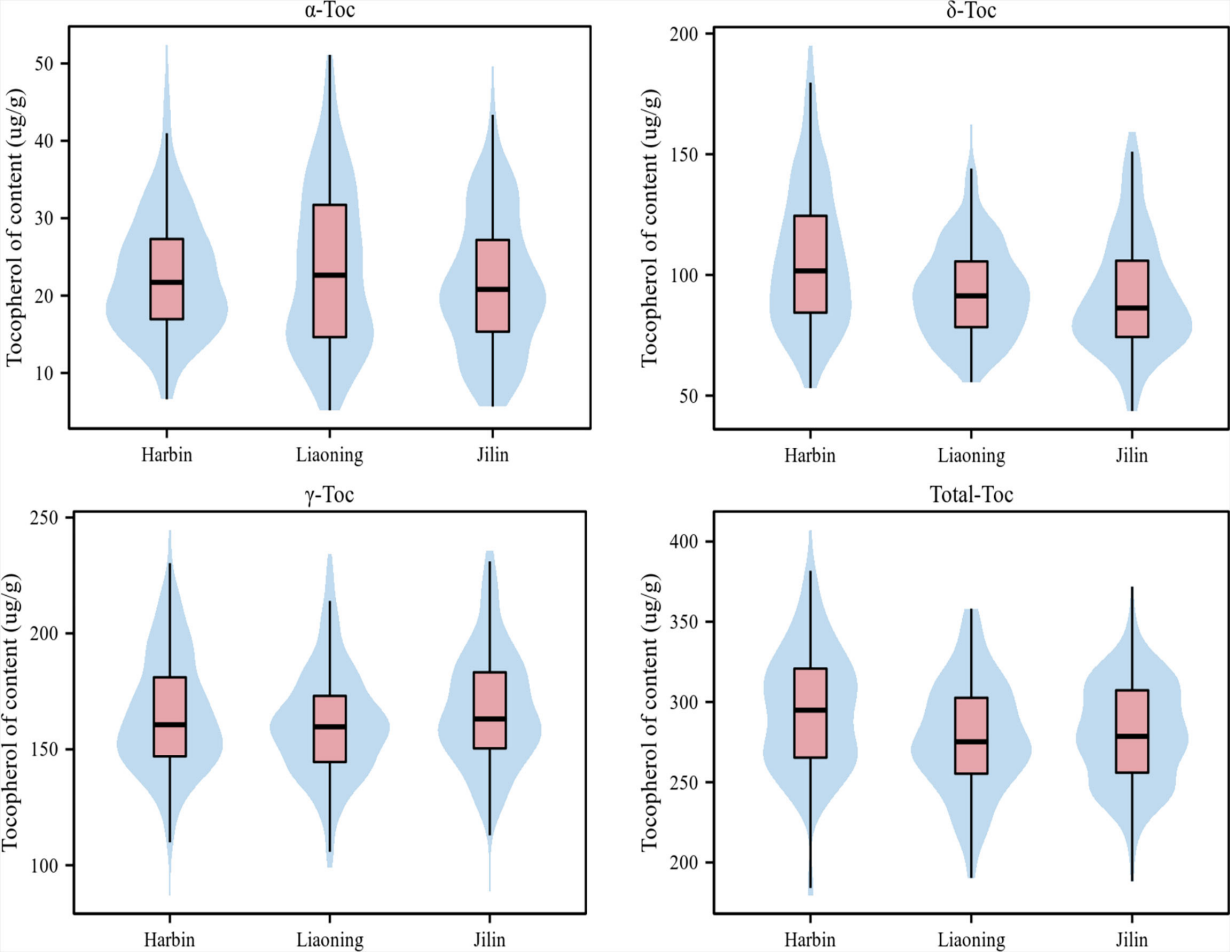
Phenotypic variation of Toc content in soybean seeds of the tested accessions at three environments. (‘Harbin’, ‘Liaoning’, and ‘Jilin’). Variation of Toc content of soybean in the association panel. The black horizontal line represents the median, the black box represents the range from the lower quartile to the upper quartile, and the black vertical line represents the dispersion of phenotypic data.

Statistical analysis showed a wide range of phenotypic variations in the levels of the individual and total Toc content of the 175 soybean accessions from Harbin, Liaoning, and Jilin in 2021 (**Table 1**). The coefficient of variation (CV%), skewness, and kurtosis of Toc content of the association panel are also presented in **Table 1**. The CV varied a lot among different Toc content, especially the α-Toc content under three locations were observed from 35.21% to 44.9%, but all Toc content was no significant skewness or kurtosis (**Figure 1**). These results showed that Toc content was mainly influenced by genetic factors with less effect by environmental factors. Therefore, the tocopherol content of soybean in this study was appropriate for GWAS.

**Table 1:** Statistical and variation analysis of tocopherol content in the tested soybean population (n = 175).

Traits	Location	Min(μg/g)	Max(μg/g)	Mean(μg/g)	CV	Skewness	Kurtosis	Heritability
α-Toc content	Harbin	6.59	52.43	22.69	35.21%	0.74	0.80	0.51
	Liaoning	5.17	51.12	23.42	44.90%	0.42	-0.64	
	Jilin	5.65	49.62	21.48	41.68%	0.41	-0.33	
γ-Toc content	Harbin	86.97	244.7	164.97	15.65%	0.35	0.17	0.59
	Liaoning	99.01	234.15	161.01	14.63%	0.38	0.55	
	Jilin	88.78	235.8	167.24	15.22%	0.29	0.26	
δ-Toc content	Harbin	53.1	195.1	107.17	27.71%	0.63	-0.12	0.72
	Liaoning	55.6	162.29	93.23	21.04%	0.54	0.21	
	Jilin	43.64	159.24	91.73	25.92%	0.70	0.12	
Total- content	Harbin	179.49	407.31	294.83	13.09%	0.03	0.24	0.64
	Liaoning	190.37	358.14	277.66	12.45%	0.01	-0.21	
	Jilin	188.34	371.91	280.44	11.41%	-0.04	-0.33	

Min, minimum; Max, maximum; CV, coefficient of variation.

### SNP genotyping, linkage disequilibrium estimating, and population structure for the GWAS panel

**Figure 2:**
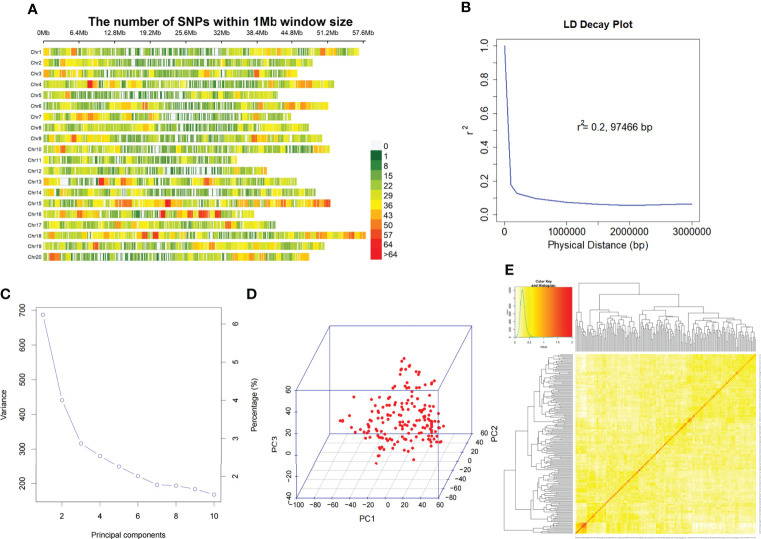
SNP density, distribution and mapping genetic data of populations. **(A)**. SNP density and distribution across 20 soybean chromosomes. **(B)**. LD decay of the genome-wide association study (GWAS) population. **(C)**. Population structure of soybean germplasm collection reflected by principal components. **(D)**. The first 3 principal components of the 23,149 SNPs used in GWAS. **(E)**. A heatmap of the kinship matrix of the 175 soybean accessions.

The genotyped samples included 175 soybean germplasms (including landraces and elite cultivars). The genomic DNA of these 175 accessions was sequenced using SLAF-seq. A total of 23,149 high-quality markers (MAF ≥ 0.05, missing data ≤ 10%) were identified from 153 million paired-end reads with 45 bp-read lengths and the sequencing depth was about 6.5 fold. The number of SNPs varied across the 20 soybean chromosomes. The highest number of SNPs was observed in Chr.18 (1732) and the lowest was detected in Chr.11 (685) (**Figure 2A**).

We assessed the mapping power of GWAS by the average distance of LD decay. The mean LD decay of the population was estimated at 97466 bp, when r^2^ dropped to 0.2 (**Figure 2B**). Then, all 23,149 SNPs were used for scanning the population stratification of association panels through the principal component (PC), and evaluation of the variation of the first 10 PCs analysis revealed an inflection point at PC3, which demonstrated that the first 3 PCs dominated the population structure on the association mapping (**Figures 2C, D**). Additionally, a lower level of genetic relatedness among the 175 tested accessions based on pairwise relative kinship coefficients was observed (**Figure 2E**).

### Quantitative trait locuss associated with Toc content by GWAS

**Figure 3:**
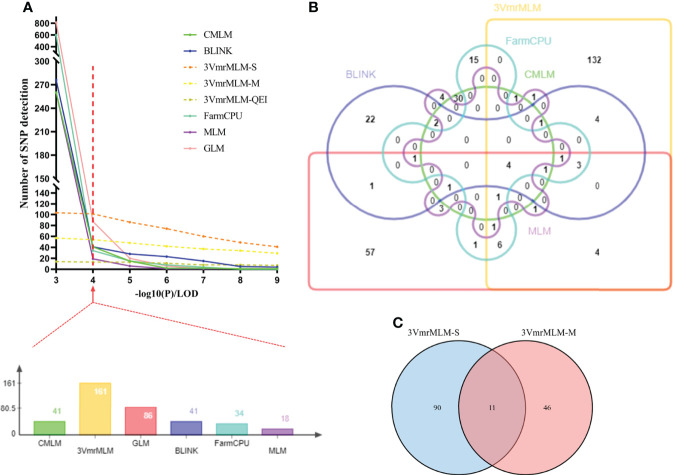
Statistics of QTLs in GWAS results under three models. **(A)** Statistics on the number of QTLs detected at different significance thresholds by different models or methods. **(B)**Venn diagram representing the number of unique and shared QTLs with six models. **(C)** Venn diagram representing the number of unique and shared QTLs with 3VmrMLM single-environment method and 3VmrMLM multiple-environment method. Finally determine the red line **(A)** represents the GWAS significance threshold of this study, both **(B, C)** are counted at this significance threshold. 3VmrMLM-S represents 3VmrMLM single-environment method, 3VmrMLM-M represents QTL detection of 3VmrMLM multiple-environment method, 3VmrMLM-QEI represents QEI detection of 3VmrMLM multiple-environment method.

**Figure 4:**
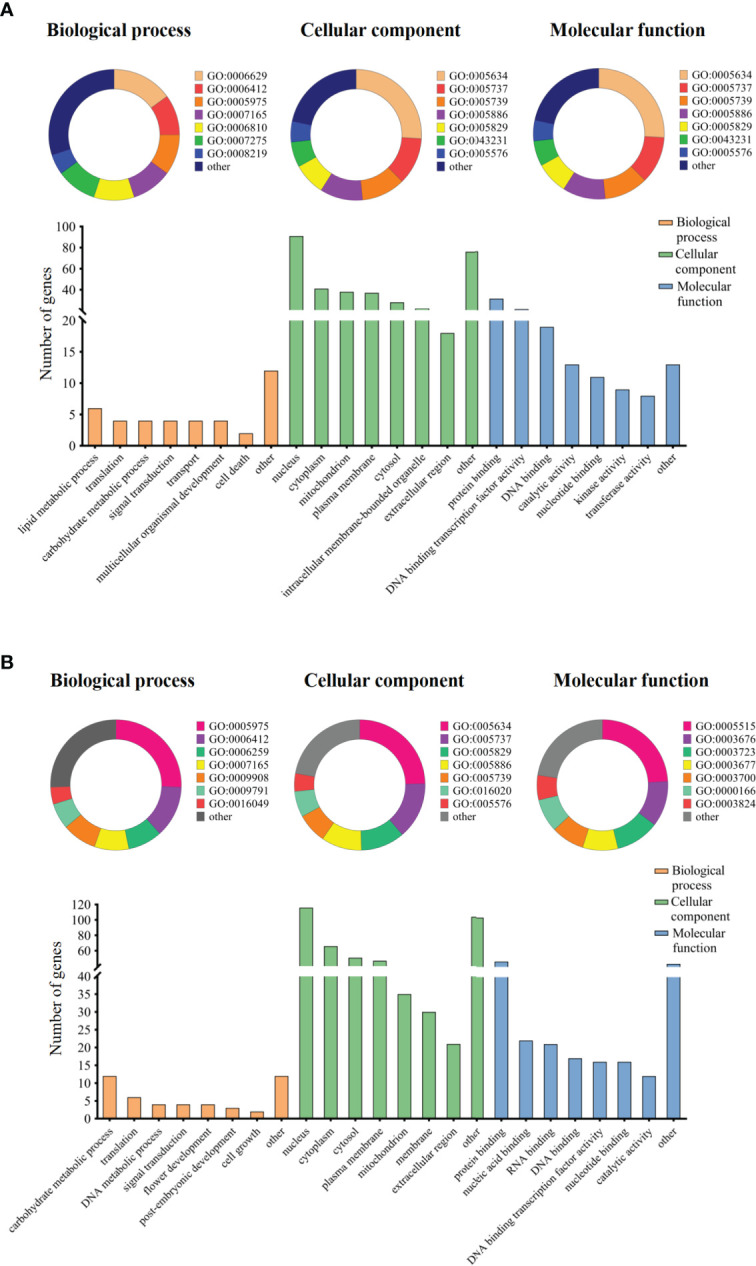
Gene ontology term enrichment analysis of candidate genes. Note: The categorized percentage and the quantity statistics of gene ontology term enrichment analysis of candidate genes, **(A)** represents group I candidate genes and **(B)** represents group II candidate genes.

GWAS was conducted using GLM, MLM, CMLM, FarmCPU, BLINK, and 3VmrMLM models. All of which accounted for kinship and population structure. First of all, we used different thresholds of significance (by -log10 (P) or LOD score= 3, 4, 5, 6, 7, 8, and 9) for testing six GWAS models and counted the number of QTLs detected (**Figure 3A**).Then, when -log10(P) ≥ 4 as significant thresholds, a total of 86 QTLs significantly associated with individual and total Toc concent in soybean seeds were detected *via* GLM, 18 QTLs were detected by MLM, 41 QTLs by CMLM, 41 QTLs by BLINK, and 34 QTLs by FarmCPU (**Figure 4A**, **Figures S1**–**S5** and **Tables S3**–**S7**). Among them, only 4 QTLs were co-detected by all six models (**Figure 3B**). Furthermore, the largest number of QTLs were detected with the 3VmrMLM model. Among them, the single-environment method detected 101 QTLs (**Figure S6**, **Table S8**), the multiple-environments method detected 57 QTLs (**Figure S7**, **Table S9**), and 13 QEIs (**Figure S8**, **Table S10**). Among them, 11 QTLs were co-detected by single-environment and multiple-environment method (**Figure 3C**). The results showed that the number of QTLs detected by 3VmrMLM are more abundant and stable under different significance thresholds.

Finally, the QTLs, which were repeatedly detected in multiple GWAS models, were selected as reliable QTLs—group I. As shown in **Figure 3B**, **Table 2**, 19 QTLs were co-detected by at least three times or at least two models, which were distributed among 24 genomic regions in 14 chromosomes. Among these, 9 QTLs (rs9337368, rs1834346, rs17125409, rs330000, rs9782629, rs19530677, rs5680781, rs17266245, and rs53062844) were located in genomic regions or QTLs reported by previous studies, confirming the accuracy of QTL detection. We regard the remaining 15 QTLs as the novel QTLs (rs39895210, rs2960931, rs19310064, rs31044180, rs7543892, rs4992837, rs14593163, rs24979561, rs588498, rs19962490, rs6204830, rs8720462, rs37558520, rs34774232, and rs35815938). Moreover, a total of 161 QTLs were identified by 3VmrMLM (**Figure 3A**), in order to test the reliability of the 3VmrMLM model, we selected the QTLs only detected in 3VmrMLM. 9 QTLs (detected by at least two times) were repeatedly detected as specific QTLs—group II (**Table 3**), which were distributed among 9 genomic regions in 8 chromosomes. rs41784197 was located in genomic regions or QTLs reported by previous studies. Again, we regard the remaining 8 QTLs as the novel QTLs (rs7167202, rs9140707, rs18105573, rs2669053, rs40595691, rs43000771, rs5779917, and rs46814888).

**Table 2:** SNPs associated with Toc content of soybean seeds and known QTLs overlapped with peak SNPs of group Ⅰ.

SNP	Chr.	Position	Allele	Traits	Model/Method	Significance	Environment	-log10(P)	Known QTL	References
rs9337368	2	9337368	A/T	δ-Toc content	BLINK		Harbin	6.48	SSR02_0458-SSR02_0520	Sui et al., 2020
				δ-Toc content	FarmCPU		Harbin	6.15		
				δ-Toc content	MLM		Harbin	4.03		
				δ-Toc content	GLM		Harbin	4.49		
				δ-Toc content	3V-M	SIG	–	6.33		
rs39895210	3	39895210	G/A	Total-Toc content	BLINK		Liaoning	4.15		
				Total-Toc content	FarmCPU		Liaoning	4.52		
				Total-Toc content	GLM		Liaoning	4.53		
				Total-Toc content	3V-S	SIG	Liaoning	19.38		
rs2960931	6	2960931	G/A	δ-Toc content	FarmCPU		Liaoning	4.45		
				δ-Toc content	GLM		Liaoning	4.47		
				δ-Toc content	3V-S	SIG	Liaoning	10.47		
				δ-Toc content	3V-M	SIG	–	10.45		
rs1834346	8	1834346	A/T	α-Toc content	MLM		Harbin	4.16	Sat_383-BARC-037229-06749	Li et al., 2016
				α-Toc content	GLM		Harbin	4.06		
				Total-Toc content	3V-M	SIG	–	11.02		
rs19310064	8	19310064	A/C	α-Toc content	CMLM		Harbin	9.02		
				α-Toc content	BLINK		Harbin	11.84		
				α-Toc content	MLM		Harbin	9.02		
				α-Toc content	GLM		Harbin	9.43		
rs31044180	9	31044180	G/T	α-Toc content	FarmCPU		Jilin	4.43		
				γ-Toc content	FarmCPU		Jilin	5.15		
				Total-Toc content	FarmCPU		Jilin	4.22		
				γ-Toc content	MLM		Jilin	4.30		
				α-Toc content	GLM		Jilin	4.43		
				γ-Toc content	GLM		Jilin	5.15		
				Total-Toc content	GLM		Jilin	4.22		
				δ-Toc content	3V-S	SIG	Jilin	18.07		
rs7543892	10	7543892	T/G	δ-Toc content	BLINK		Jilin	7.07		
				δ-Toc content	FarmCPU		Jilin	4.55		
				δ-Toc content	GLM		Jilin	4.25		
				δ-Toc content	3V-M	SIG	–	11.13		
rs49928375	10	49928375	G/T	α-Toc content	CMLM		Harbin	5.04		
				α-Toc content	FarmCPU		Harbin	4.67		
				α-Toc content	MLM		Harbin	4.91		
				α-Toc content	GLM		Harbin	5.74		
				α-Toc content	3V-S	SIG	Harbin	17.93		
rs17125409	12	17125409	C/A	α-Toc content	CMLM		Jilin	5.21	–	Zhan et al., 2020
				α-Toc content	BLINK		Harbin	6.09		
				α-Toc content	BLINK		Jilin	10.27		
				α-Toc content	FarmCPU		Harbin	7.63		
				α-Toc content	GLM		Harbin	4.56		
				α-Toc content	3V-M	SIG	–	46.05		
rs330000	13	330000	G/A	δ-Toc content	FarmCPU		Liaoning	4.76	–	Zhan et al., 2020
				δ-Toc content	GLM		Harbin	4.76		
				δ-Toc content	GLM		Liaoning	4.96		
				δ-Toc content	3V-S	SIG	Liaoning	9.65		
				δ-Toc content	3V-M	SUG	–	4.35		
rs9782629	14	9782629	G/T	γ-Toc content	CMLM		Harbin	5.63	BARC-059251-15691-Sct_034	Shaw et al., 2017
				γ-Toc content	BLINK		Harbin	7.27		
				γ-Toc content	FarmCPU		Harbin	4.59		
				γ-Toc content	MLM		Harbin	4.71		
				γ-Toc content	GLM		Harbin	4.89		
				γ-Toc content	3V-QEI	SIG	–	15.98		
				Total-Toc content	3V-QEI	SIG	–	18.12		
rs19530677	16	19530677	T/A	γ-Toc content	CMLM		Harbin	7.33	Sat_259-Sat_370	Li et al., 2010/ Li et al., 2016
				Total-Toc content	CMLM		Harbin	6.67		
				γ-Toc content	BLINK		Harbin	9.16		
				Total-Toc content	BLINK		Harbin	4.23		
				γ-Toc content	FarmCPU		Harbin	6.10		
				γ-Toc content	MLM		Harbin	5.28		
				γ-Toc content	GLM		Harbin	6.20		
				γ-Toc content	3V-QEI	SIG	–	32.05		
rs14593163	17	14593163	T/G	δ-Toc content	BLINK		Harbin	6.42		
				Total-Toc content	BLINK		Harbin	4.56		
				Total-Toc content	FarmCPU		Harbin	7.49		
				Total-Toc content	MLM		Harbin	5.35		
				δ-Toc content	GLM		Harbin	4.73		
				Total-Toc content	GLM		Harbin	6.12		
rs24979561	17	24979561	G/A	α-Toc content	CMLM		Harbin	5.87		
				α-Toc content	BLINK		Harbin	7.86		
				α-Toc content	FarmCPU		Harbin	7.56		
				α-Toc content	MLM		Harbin	5.87		
				α-Toc content	GLM		Harbin	6.53		
				α-Toc content	3V-S	SIG	Harbin	18.72		
rs588498	18	588498	G/A	α-Toc content	FarmCPU		Liaoning	4.26		
				α-Toc content	GLM		Liaoning	4.26		
				α-Toc content	3V-S	SUG	Liaoning	4.53		
rs5680781	18	5680781	G/T	Total-Toc content	CMLM		Jilin	5.04	–	Zhan et al., 2020
				γ-Toc content	BLINK		Jilin	4.62		
				Total-Toc content	BLINK		Jilin	5.61		
				γ-Toc content	3V-S	SIG	Jilin	9.07		
rs17266245	18	17266245	T/G	γ-Toc content	BLINK		Jilin	4.31	Satt038–Sat_164	Sui et al., 2020/ Zhan et al., 2020
				γ-Toc content	3V-S	SIG	Jilin	16.18		
				γ-Toc content	3V-M	SIG	–	11.10		
rs19962490	18	19962490	T/C	δ-Toc content	MLM		Harbin	5.42		
				Total-Toc content	MLM		Harbin	4.39		
				δ-Toc content	GLM		Harbin	4.84		
				Total-Toc content	GLM		Harbin	4.37		
rs53062844	18	53062844	G/T	α-Toc content	CMLM		Liaoning	4.91	Satt472–Satt038	Sui et al., 2020
				α-Toc content	BLINK		Liaoning	12.82		
				α-Toc content	FarmCPU		Liaoning	5.73		
				α-Toc content	MLM		Liaoning	4.87		
				α-Toc content	GLM		Liaoning	5.73		
				α-Toc content	3V-M	SIG	–	23.60		
rs6204830	19	6204830	T/G	α-Toc content	MLM		Liaoning	4.10		
				α-Toc content	3V-S	SIG	Liaoning	15.42		
				α-Toc content	3V-S	SIG	Jilin	7.40		
rs8720462	19	8720462	G/A	δ-Toc content	BLINK		Harbin	7.38		
				δ-Toc content	FarmCPU		Harbin	4.37		
				δ-Toc content	FarmCPU		Liaoning	4.48		
				δ-Toc content	GLM		Harbin	6.82		
				δ-Toc content	GLM		Liaoning	4.48		
				δ-Toc content	3V-S	SUG	Harbin	5.61		
				δ-Toc content	3V-M	SUG	–	4.01		
rs37558520	19	37558520	T/C	Total-Toc content	FarmCPU		Liaoning	4.06		
				Total-Toc content	GLM		Liaoning	4.09		
				α-Toc content	3V-S	SIG	Liaoning	9.34		
rs34774232	20	34774232	A/G	δ-Toc content	FarmCPU		Harbin	4.72		
				δ-Toc content	GLM		Harbin	4.82		
				δ-Toc content	3V-M	SIG	–	13.07		
rs35815938	20	35815938	T/C	δ-Toc content	FarmCPU		Liaoning	4.14		
				δ-Toc content	GLM		Liaoning	4.12		
				δ-Toc content	3V-M	SIG	–	10.32		

3V-S represents 3VmrMLM single-environment method, 3V-M represents QTL detection of 3VmrMLM multiple-environment method, 3V-QEI represents QEI detection of 3VmrMLM multiple-environment method, SIG represents significant QTLs, and SUG represents suggested QTLs.

**Table 3:** SNPs associated with Toc content of soybean seeds and known QTLs overlapped with peak SNPs of group Ⅱ.

SNP	Chr.	Position	Allele	Traits	Model/Method	Environment	$-\log_{10}^{\left(P\right)}$	Known QTL	References	Significance
rs7167202	1	7167202	G/T	γ-Toc content	3VmrMLM-S	Jilin	5.13			SUG
				Total-Toc content	3VmrMLM-S	Jilin	6.43			SIG
				Total-Toc content	3VmrMLM-M	–	16.28			SIG
rs41784197	1	41784197	T/C	γ-Toc content	3VmrMLM-S	Jilin	11.64	Satt179-Sat_201	Li et al., 2016	SIG
				γ-Toc content	3VmrMLM-M	–	12.32			SIG
rs9140707	7	9140707	G/T	α-Toc content	3VmrMLM-S	Liaoning	17.35			SIG
				α-Toc content	3VmrMLM-M	–	33.30			SIG
rs18105573	8	18105573	A/G	δ-Toc content	3VmrMLM-S	Jilin	6.44			SIG
				δ-Toc content	3VmrMLM-M	–	5.58			SUG
rs2669053	9	2669053	T/C	γ-Toc content	3VmrMLM-S	Harbin	16.30			SIG
				γ-Toc content	3VmrMLM-QEI	–	11.49			SIG
				Total-Toc content	3VmrMLM-S	Harbin	12.13			SIG
				Total-Toc content	3VmrMLM-QEI	–	5.23			SUG
rs40595691	10	40595691	C/T	γ-Toc content	3VmrMLM-M	–	4.35			SUG
				Total-Toc content	3VmrMLM-M	–	7.34			SIG
rs43000771	15	43000771	C/T	γ-Toc content	3VmrMLM-S	Liaoning	4.07			SUG
				Total-Toc content	3VmrMLM-M	–	4.57			SUG
rs5779917	19	5779917	G/T	α-Toc content	3VmrMLM-QEI	–	8.90			SIG
				γ-Toc content	3VmrMLM-S	Harbin	7.76			SIG
rs46814888	20	46814888	T/G	δ-Toc content	3VmrMLM-S	Harbin	8.64			SIG
				δ-Toc content	3VmrMLM-M	–	8.70			SIG

3V-S represents 3VmrMLM single-environment method, 3V-M represents QTL detection of 3VmrMLM multiple-environment method, 3V-QEI represents QEI detection of 3VmrMLM multiple-environment method, SIG represents significant QTLs, and SUG represents suggested QTLs.

### Prediction of candidate genes for Toc content in soybean seeds

Based on annotations for the soybean reference genome in SoyBase, we further predicted candidate genes within the 200-kb flanking regions of the novel QTLs. In two group novel QTLs, a total of 248 genes were obtained (**Table S11**). And a total of 134 genes were obtained in QEIs (**Table S12**). Then, we used GO annotation to perform enrichment analysis for group I and group II genes. The results categorized as molecular function, cellular component, and biological process, were shown in **Figure 4**. Both group I and group II candidate genes are involved in a variety of functions, such as carbohydrate metabolic process, translation, protein binding, cytoplasm component, DNA binding, and so on.

### Comparative genome analysis

**Figure 5:**
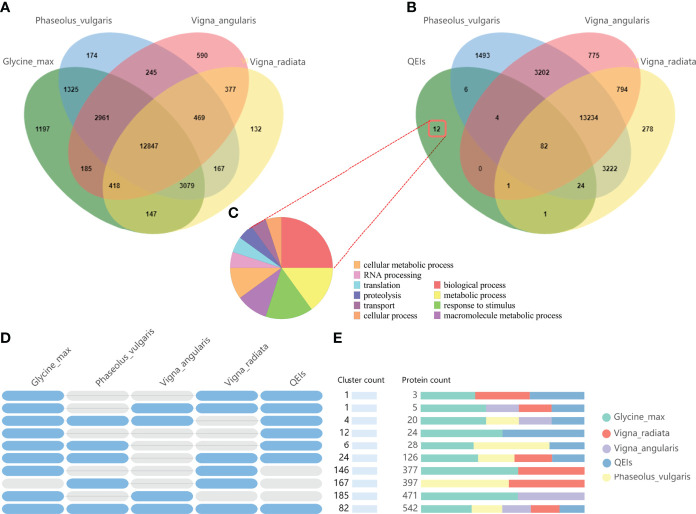
Comparative genome analysis candidate genes of QEIs. **(A)**. Venn diagram representing the core orthologs and specific genes cluster for *Glyma_max*, *Vigna_radiate*, *Vigna_augularis*, and *Phaseolus_vulgaris*. **(B)**. Venn diagram representing the core orthologs and specific genes cluster for candidate genes of QEIs, *Vigna_radiate*, *Vigna_augularis*, and *Phaseolus_vulgaris*. **(C)**. Gene ontology term enrichment analysis of unique candidate genes of QEIs. **(D)**. Shared gene clusters of orthologous groups categories. **(E)**. Protein families count shared between *Glyma_max*, *Vigna_radiate*, *Vigna_augularis*, *Phaseolus_vulgaris*, and candidate genes of QEIs.

**Figure 6:**
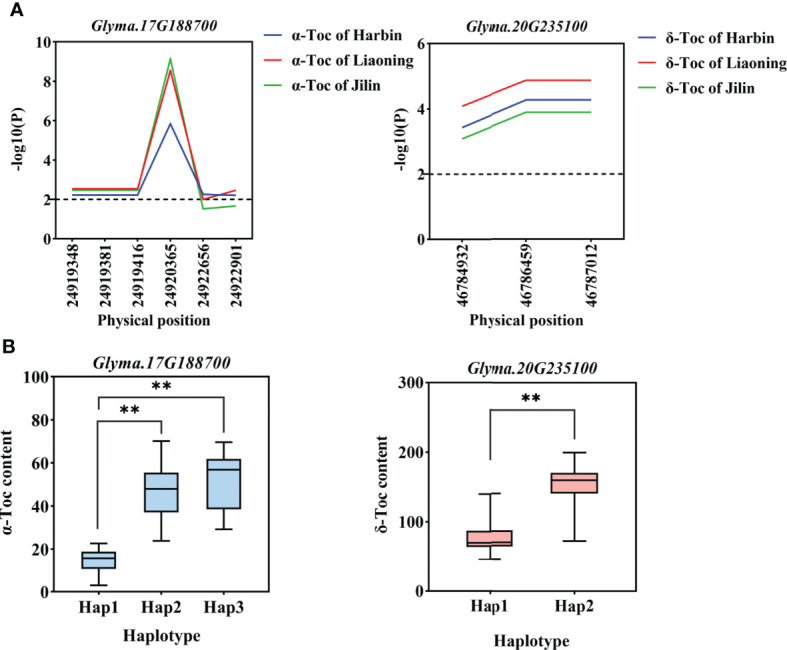
Gene-based association analysis and haplotypes analysis. **(A)**. Gene-based association analysis of candidate genes that related to Toc content. **(B)**. Haplotypes analysis of candidate genes that related to Toc content. Horizontal line indicates that the threshold is set to 2.0, the * and ** was significance at *P* < 0.05 and *P* < 0.01, respectively, *Glyma.17G188700* from group I, and *Glyma.20G235100* from group II.

In order to predict the authenticity of the QEIs, firstly, we selected four closely related species, *Glyma_max*, *Vigna_radiate*, *Vigna_augularis*, and *Phaseolus_vulgaris*,for comparative genomic analysis. A total of 12847 core gene clusters were found in the four species, and 1197 gene clusters were unique to *Glyma_max* (**Figure 5A**), specific genes clusters account for 5.4% (1197/22159). Then, we used candidate gene of QEIs for comparative genomic analysis, 12 gene clusters were unique to candidate gene of QEIs (**Figure 5B**), specific genes clusters account 9.23% (12/130), this result shown that these QEIs have more abundant specific genes. As shown in **Figure 5C**, these specific genes are involved in various biological processes, metabolic processes, response to stimulus, etc. More detailed statistics on the number of shared gene clusters are shown in **Figure 5D**. **Figure 6E** is count of proteins by type of cluster.

### Gene-based association analysis of candidate genes

Two groups of candidate gene association analysis were performed using the GLM model with the TASSEL, using the genome resequencing of 56 germplasms (including 9 high and low individual and total Toc germplasms). A total of 4537 SNPs with MAF ≥ 0.05 were identified among 248 candidate genes. Among them, a total of 50 SNPs from 11 candidate genes were found to reach the threshold with -log10(P) ≥ 2.0 (**Table S13**), of these, 4 SNPs are located in upstream regions, 10 SNPs are located in intronic regions, 26 SNPs are located in exonic ;regions, and 10 SNPs are located ;in downstream regions. Those SNPs are considered to be significantly associated with individual and total Toc concentrations in soybean seeds. Among these genes, 4 candidate genes from group I and 7 candidate genes from group II. These genes can be considered potential candidate genes for individual and total Toc-related. For example, as shown in **Figure 6A**, the significant SNPs correlated to α‐Toc and δ‐Toc on basis of association analysis for two candidate genes were respectively identified (*Glyma.17G188700* and *Glyma.20G235100* were shown in **Figure 6A**, others were shown in **Figure S9**).

### Haplotype analysis of candidate genes

For the haplotype analysis, first, all the SNP markers within each gene are used to construct haplotypes. Then, we performed one-way ANOVA with TC-BLUP values of each soybean accession. The results are shown in **Table 4**, each gene contains haplotypes that are significant differences from TC-BLUP values. In addition, 14 haplotypes of 11 candidate genes respectively conferred an increased individual and total Toc content in soybean seeds (*Glyma.17G188700* and *Glyma.20G235100* were shown in **Figure 6B**, others were shown in **Figure S10**). Therefore, these haplotypes are beneficial and can be adjusted for individual and total Toc content in soybean seeds.

**Table 4:** Haplotype analysis of candidate genes.

Gene ID	Traits	Hap	Total number	Mean TC-BLUP value	P value	Significance	Functional annotation	References
*Glyma.03G186200*	Total-Toc content	Hap1	9	241.55	–	–	RAB GTPase homolog C2A	
		Hap2	3	307.98	0.0007	***		
		Hap3	6	310.88	<0.0001	****		
*Glyma.03G186500*	Total-Toc content	Hap1	9	241.55	–	–	Transducin family protein/WD-40 repeat family protein	
		Hap2	7	305.21	<0.0001	****		
		Hap3	2	326.38	0.0002	***		
*Glyma.06G038000*	δ-Toc content	Hap1	9	79.08	–	–	Alpha/beta-Hydrolases superfamily protein	Albert et al., 2022
		Hap2	9	129.63	<0.0001	****		
*Glyma.17G188700*	α-Toc content	Hap1	7	12.19	–	–	hAT dimerisation domain-containing protein/transposase-related	
		Hap2	6	28.82	0.0053	**		
		Hap3	5	33.21	0.0013	**		
*Glyma.01G054800*	γ-Toc content	Hap1	4	107.76	–	–	Plant protein of unknown function (DUF863)	
		Hap2	5	107.66	>0.9999	ns		
		Hap3	3	206.32	0.0002	***		
		Hap4	6	201.99	<0.0001	****		
	Total-Toc content	Hap1	4	271.77	–	–		
		Hap2	5	298.58	0.6572	ns		
		Hap3	3	262.08	0.9795	ns		
		Hap4	6	266.15	0.9932	ns		
*Glyma.08G222300*	δ-Toc content	Hap1	4	93.7	–	–	O-fucosyltransferase family protein	
		Hap2	3	120.1	0.5826	ns		
		Hap3	3	156.71	0.0548	ns		
		Hap4	8	199.39	0.0003	***		
*Glyma.09G032100*	Total-Toc content	Hap1	5	247.64	–	–	MYB domain protein 78	
		Hap2	4	233.93	0.5327	ns		
		Hap3	9	300.91	0.0002	***		
	γ-Toc content	Hap1	5	102.56	–	–		
		Hap2	4	114.14	0.6506	ns		
		Hap3	9	203.43	<0.0001	****		
*Glyma.10G171600*	Total-Toc content	Hap1	5	306.77	–	–	RAB GTPase homolog A5A	
		Hap2	4	313.84	0.9366	ns		
		Hap3	4	238.98	0.0013	**		
		Hap4	5	243.6	0.0014	**		
	γ-Toc content	Hap1	5	105.48	–	–		
		Hap2	4	110.49	0.9775	ns		
		Hap3	4	200.26	<0.0001	****		
		Hap4	5	205.98	<0.0001	****		
*Glyma.20G235100*	δ-Toc content	Hap1	8	86.25	–	–	Indeterminate(ID)- domain 2	
		Hap2	10	118.84	0.0089	**		
*Glyma.20G235400*	δ-Toc content	Hap1	6	84	–	–	P-loop containing nucleoside triphosphate hydrolases superfamily protein	
		Hap2	4	91.82	0.7330	ns		
		Hap3	8	131.88	0.0004	***		
*Glyma.20G235800*	δ-Toc content	Hap1	6	91.24	–	–	Transducin/WD40 repeat-like superfamily protein	
		Hap2	5	78.23	0.2695	ns		
		Hap3	7	134.25	0.0002	***		

Hap represents Haplotype, TC represents individual and total Toc content. P < 0.05 was considered significant, * Significance was P < 0.05, ** Significance was P <0.01, *** Significance was P < 0.001, **** Significance was P < 0.0001 and ns stands for no significance.

### RNA-Seq data analysis of candidate genes for Toc content in soybean

**Figure 7:**
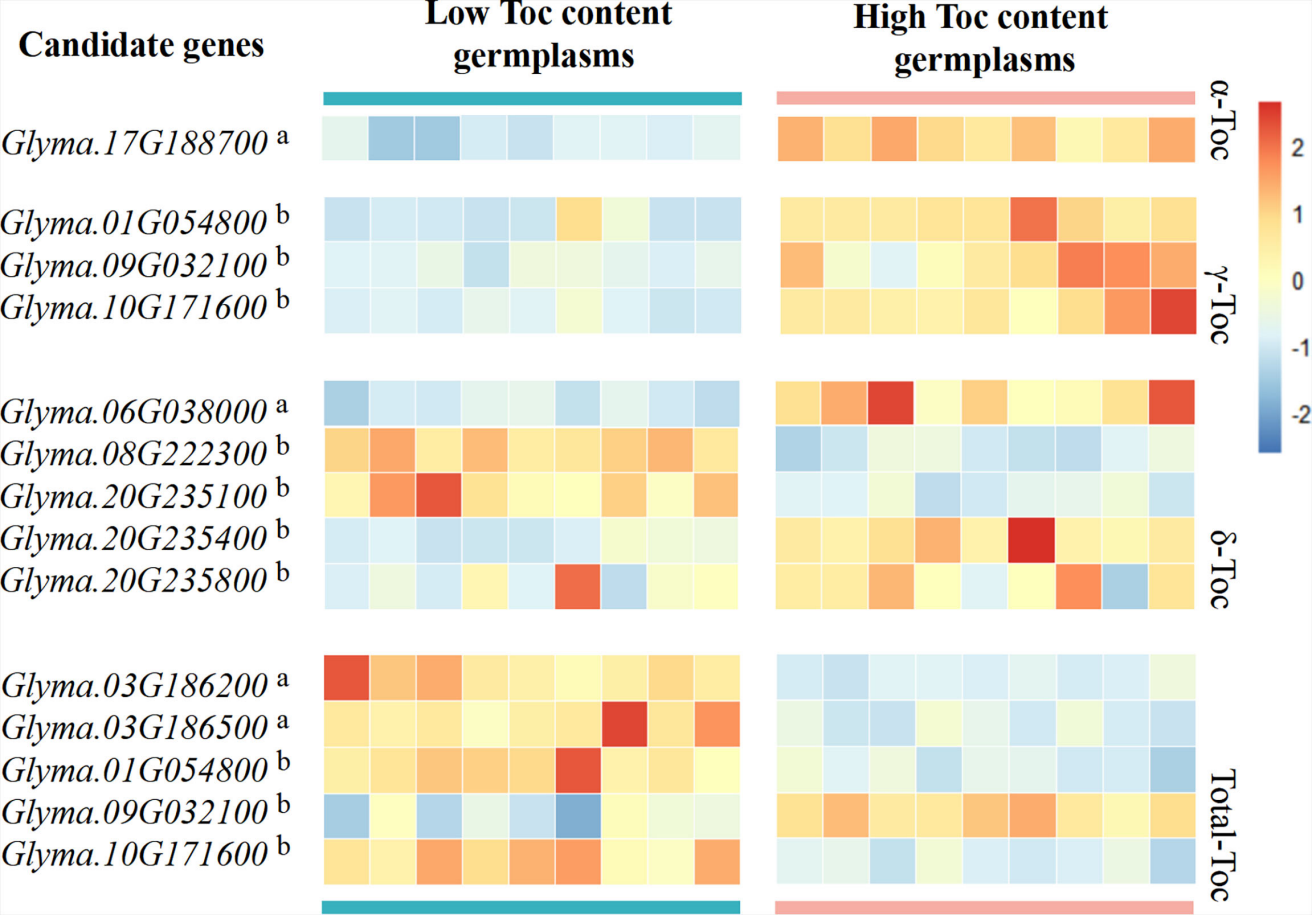
Heatmap of candidate gene expression analysis by RNA-Seq data. Candidate gene analysis was performed using different high and low germplasms for each Toc content, the red boxes indicate high transcript levels, and the blue boxes indicate low transcript levels. The letter in the upper right corner a indicates the gene from group I, and the letter in the upper right corner b indicates the gene from group II.

In order to confirm the possible effect of candidate genes in the regulation of Toc content, we firstly used PPRD to analyze the expression patterns of 11 candidate genes in different tissues. The result showed that all candidate genes were expressed in soybean seed (**Figure S11**), and *Glyma.10G171600* is most abundantly expressed in seed compared with other tissues. Then, for the 11 candidate genes of 56 soybean germplasms at the development stage (R6), RNA-Seq data analysis was done. The result showed that the expression levels of the 11 candidate genes in low and high Toc content germplasms were different. Among them, *Glyma.17G188700* can regulate α-Toc content in soybean seeds. The range of the expression levels of *Glyma.17G188700* in higher α-Toc germplasms was much higher than those of lower. Other genes regulate Toc content as shown in **Figure 7**. Interestingly, *Glyma.01G054800*, *Glyma.09G032100*, and *Glyma.10G171600* can regulate both the γ-Toc and Total-Toc content. *Glyma.09G032100* in higher γ-Toc and total-Toc germplasms were much higher than those expression levels of lower. However, *Glyma.01G054800*, and *Glyma.10G171600* in higher γ-Toc germplasms have higher expression levels, but in higher total-Toc germplasms have lower expression levels. Moreover, these candidate genes results of qRT-PCR are consistent with the RNA-seq data (**Figure S12**).

## Discussion

As one of the vitamin E family members, Toc plays a crucial role for humans, plants, and animals (Bramley et al., 2000). For humans, daily Toc supplementation can decrease the risk for cancer and cardiovascular disease (Shaw et al., 2016). For plants, Toc can protection of chloroplasts from photooxidative damage (Munne-Bosch and Alegre, 2002). For animals, Toc must be added to animal feed to improve and maintain growth and health (Pinelli-Saavedra et al., 2008). Soybean is a major crop used worldwide as a source of food, oil, and animal feed. Soybean oil compared to other oil crops contains a higher total Toc content, but γ-Toc comprises 70% (Park et al., 2019). The physiological activity of γ-Toc was lower than that of α-Toc (Wan et al., 2008). Therefore, increasing the α-Toc and total Toc content in soybean seeds is important to improve the nutritional variety and feed quality of soybean. However, the genetic background of Toc content is complex quantitative inheritance. The reason why quantitative traits are complex is that they are controlled by unequal polygenes and are susceptible to environmental influences. In this study, individual and total Toc content of 175 soybean accessions were evaluated. The results showed that the Toc content of tested germplasms was relatively stable to the environment, and Toc content had a wide range of variation among the different germplasms.

GWAS has been widely used in the mining of QTLs in most crops including soybean. It is a method to identify the genetic variation among the natural populations to establish genetic markers based on linkage disequilibrium (LD) (Yano et al., 2019; Xiao et al., 2022). How improve the power of GWAS has been a major challenge for the last decade. In recent years, a variety of new methods have been proposed, with the rapid development of computing technology and sequencing technology (Wang et al., 2016; Huang et al., 2018; Xiao et al., 2021; Li et al., 2022a). Although this propelled much of the practicability of GWAS, it is particularly important to select the appropriate sequencing method and suitable model for improving the positioning efficiency according to the research needs (Liu et al., 2017; Kim et al., 2021). For this study, we adopted six models (GLM, MLM, CMLM, BLINK, FarmCPU, and 3VmrMLM), to conduct GWAS of Toc content in soybean seeds. And the results were divided into two groups, revealed a total of 23 novel QTLs, other QTLs were located in the regions of QTLs in previous studies or overlapped our previous GWAS studies, and these known QTLs are all covered by 3VmrMLM.

3VmrMLM is a new algorithm, different from other algorithm, the 3VmrMLM use single-marker genome-wide scanning to select potentially associated markers and uses empirical Bayes and the likelihood ratio test in a multi-locus model to identify significant QTLs, this undoubtedly improves its detection capability (Li et al., 2022a). Additionally, it can be simultaneously estimated in a vector manner that QEI and QQI effects. Although the QQI detection in this study did not achieve good results, the 3VmrMLM still showed better detection ability than the GLM, MLM, CMLM, BLINK, and FarmCPU, indicating a more reliable tool for complex trait dissection.

In soybean and other plants, only a few definite genes have been characterized, associated with an individual or total Toc. Among them also includes most of the key enzyme genes (Dwiyanti et al., 2011; Zhang et al., 2013). To accurately screen candidate genes, we selected a total of 248 genes within the 200-kb flanking regions of the 23 novel QTLs and using a gene-based association by the GLM method, a total of 11 genes were finally determined to be significantly related to individual or total Toc in soybean seeds. Moreover, almost all these genes have beneficial haplotypes. *Glyma.06G038000* encoded alpha/beta-Hydrolases superfamily protein. *Glyma.01G054800* encoded plant proteins of unknown function, *Glyma.03G186500* encoded a WD-40 repeat family protein, *Glyma.20G235800* encoded a WD40 repeat-like superfamily protein, Glyma.03G186200 is a RAB GTPase homolog C2A, *Glyma.10G171600* encoded a RAB GTPase homolog A5A, *Glyma.17G188700* encoded transposas, *Glyma.09G032100* encoded a myb domain protein, *Glyma.20G235100* encoded an indeterminate domain protein, *Glyma.20G235400* encoded a P-loop containing nucleoside triphosphate hydrolases superfamily protein. Of these genes, *Glyma.01G054800* and *Glyma.10G171600* are the most special, and these two genes are higher expressed in higher γ-Toc content germplasms, but lower expressed in higher total-Toc content germplasms. The soybean oil contains a higher proportion of γ-Toc, this is very different from the other oil crops (Cahoon et al., 2003). Therefore, we conclude that the *Glyma.01G054800* and *Glyma.10G171600* inhibited the transformation of α-Toc and δ-Toc, resulting in the excessive accumulation of γ-Toc, while the total-Toc content decreased. This requires further experiments to prove. The precise functions and mechanisms of 11 candidate genes will be planned in future studies.

In general, the 3VmrMLM algorithm achieved good results in the GWAS. In this study, Toc content in soybean seed in group I QTLs, 10 known QTLs are all covered by 3VmrMLM. The results of GO enrichment analysis showed that group I; and group II candidate genes had similar GO biological process terms. for the 11 candidate genes finally identified in this study, 7 genes were alone identified by the 3VmrMLM. All candidate genes were able to detected by the 3VmrMLM. In addition, a higher percentage of the *Glyma_max* specific genes have also been found in candidate genes near QEIs by comparative genomic analysis. These results have preliminarily determined the detection efficiency of the 3VmrMLM algorithm. Thus, we hope that using 3VmrMLM could be used to dissect more important complex quantitative traits in the future, and this algorithm is advantageous to promoting the development of soybean breeding.

## Data availability statement

The data presented in the study are deposited in the EBI repository, accession number PRJEB55008. Any queries should be directed to the corresponding author.

## Author contributions

KWY, and XZ conceived the study and contributed to population development. KWY, HRM, and HLL contributed to phenotypic evaluation. JHZ, and MNS analyzed the data. YHZ, and NX contributed to genotyping. KWY, XZ, and YPH contributed to experimental design and writing the paper. All authors contributed to the article and approved the submitted version.

## Funding

This study was financially supported by National Key Research and Development Project of China (2021YFF1001204), the Chinese National Natural Science Foundation (31971967, 31871650), National Key Research and Development Program of China (2021YFD1201604, 2019YFD1002601), the Youth and Middle-aged Scientific and Technological Innovation Leading Talents Program of the Crops (2015RA228), the National Ten Thousand Talent Program (W03020275), Postdoctoral Scientific Research Development Fund of Heilongjiang Province (LBH-Z15017, LBH-Q20004), Program on Industrial Technology System of National Soybean (CARS-04-PS06).

## Acknowledgments

This study was conducted in the Key Laboratory of Soybean Biology of the Chinese Education Ministry, Soybean Research & Development Center (CARS) and the Key Laboratory of Northeastern Soybean Biology and Breeding/Genetics of the Chinese Agriculture Ministry.

## Conflict of interest

The authors declare that the research was conducted in the absence of any commercial or financial relationships that could be construed as a potential conflict of interest.

## Publisher’s note

All claims expressed in this article are solely those of the authors and do not necessarily represent those of their affiliated organizations, or those of the publisher, the editors and the reviewers. Any product that may be evaluated in this article, or claim that may be made by its manufacturer, is not guaranteed or endorsed by the publisher.
