# Supplementary material for: Genome-wide association studies reveal novel QTLs, QTL-by-environment interactions and their candidate genes for tocopherol content in soybean seed
Source: Front Plant Sci. 2022 Oct 27;13:1026581. doi: 10.3389/fpls.2022.1026581 (PMC9647135; doi:10.3389/fpls.2022.1026581)
Supplement: Supplementary file 1 [file DataSheet_1.docx]

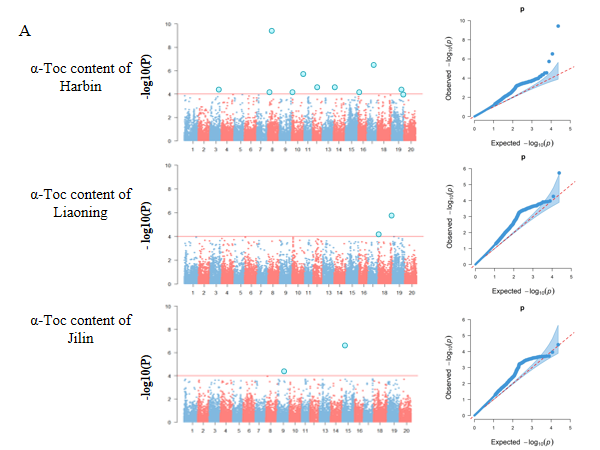

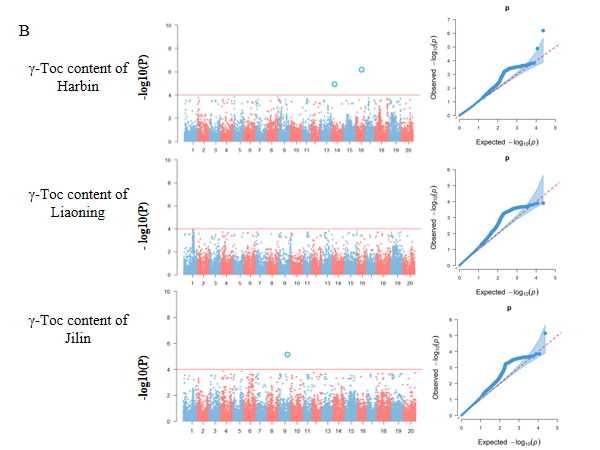

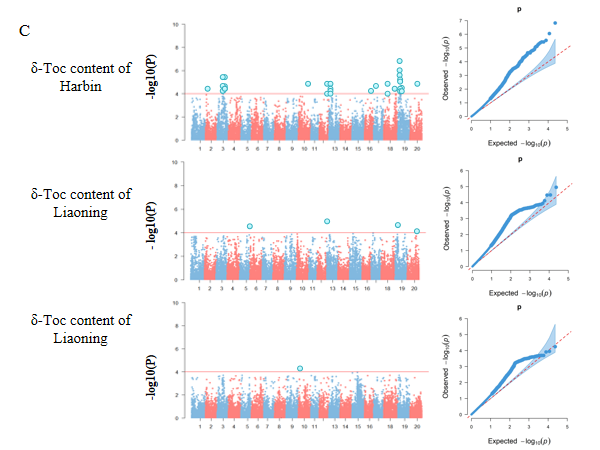

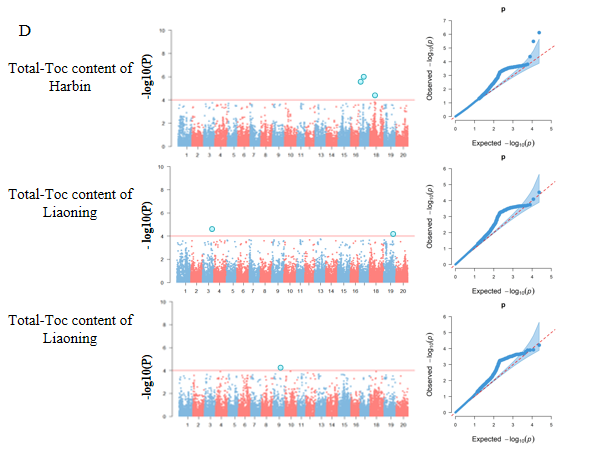


**Figure S1** Manhattan plot and Q-Q plot generated from GWAS for tocopherol content in soybean seeds among 175 soybean accessions using GLM model.

Note: A–D respectively represent α-Toc, δ-Toc, γ-Toc, and Total-Toc content in three environments.


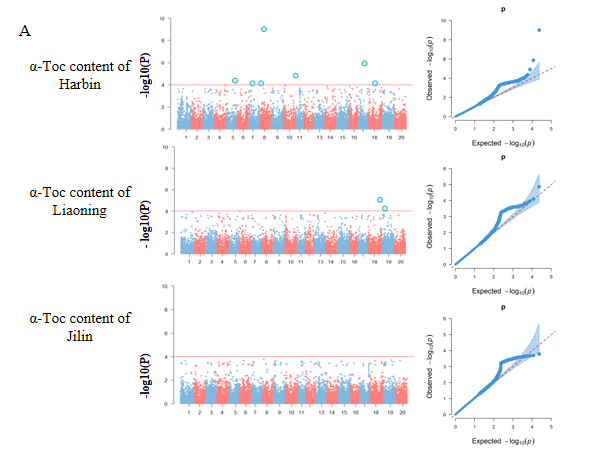

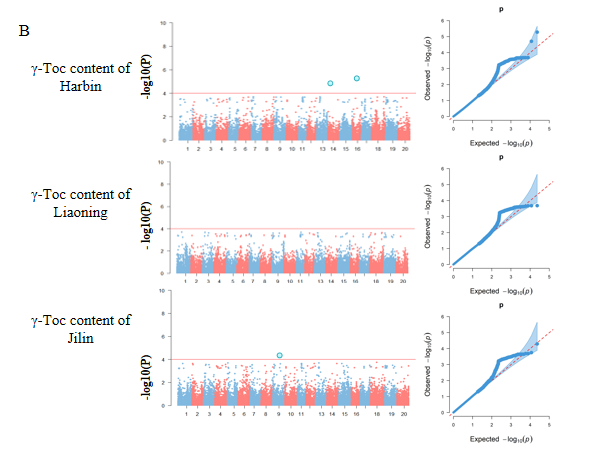

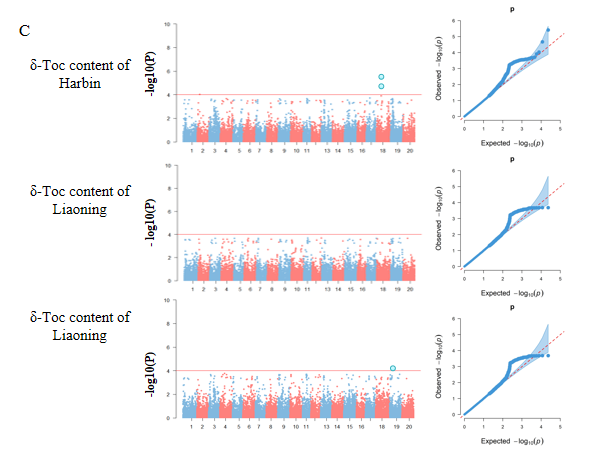

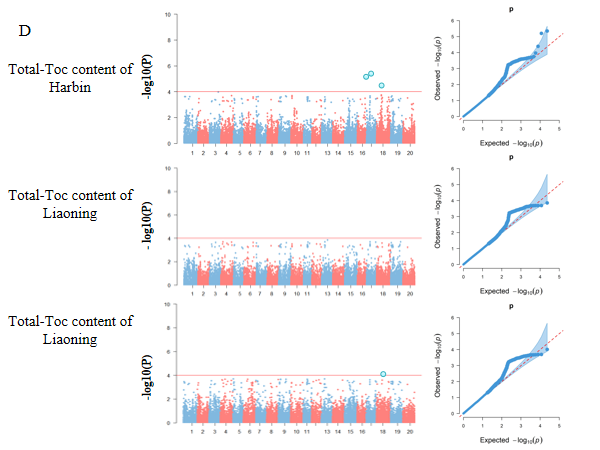


**Figure S2** Manhattan plot and Q-Q plot generated from GWAS for tocopherol content in soybean seeds among 175 soybean accessions using MLM model.

Note: A–D respectively represent α-Toc, δ-Toc, γ-Toc, and Total-Toc content in three environments.


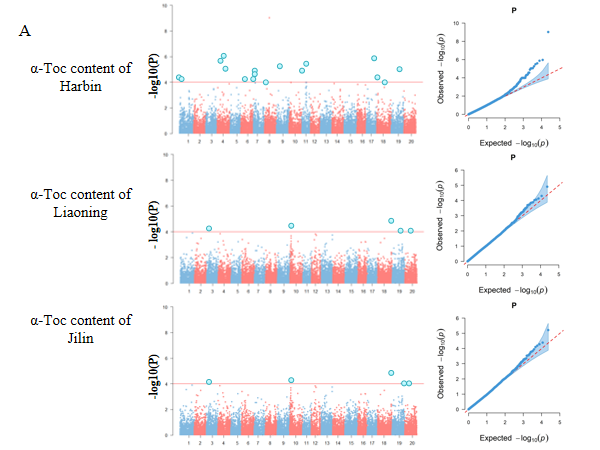


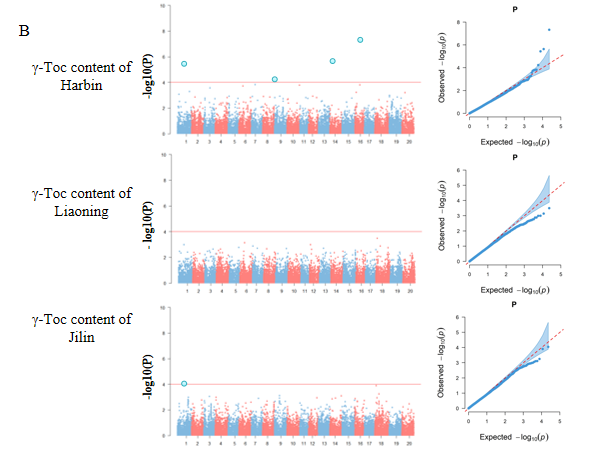

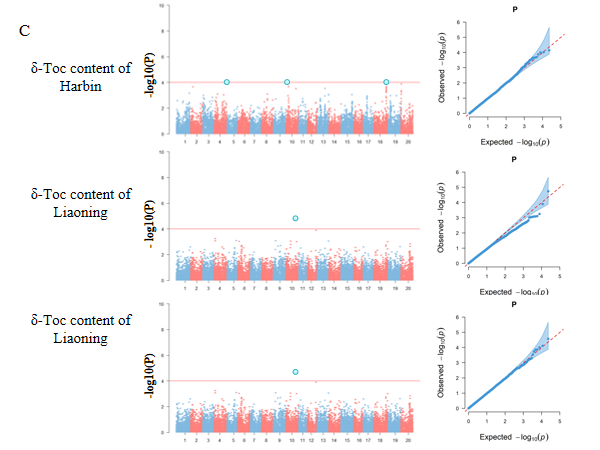


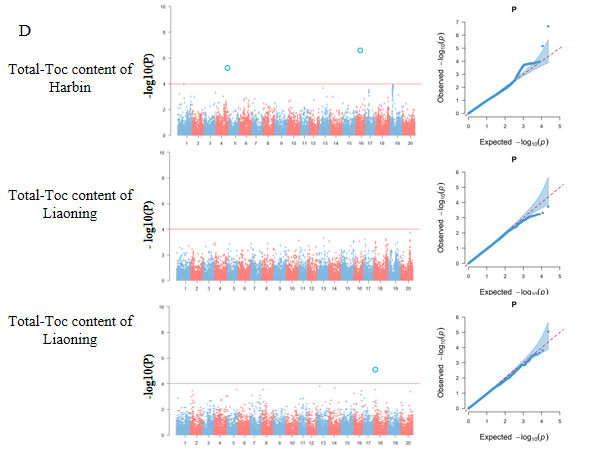


**Figure S3** Manhattan plot and Q-Q plot generated from GWAS for tocopherol content in soybean seeds among 175 soybean accessions using CMLM model.

Note: A–D respectively represent α-Toc, δ-Toc, γ-Toc, and Total-Toc content in three environments.


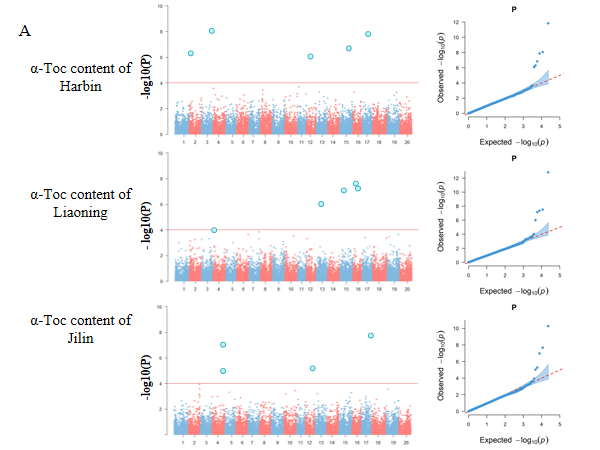

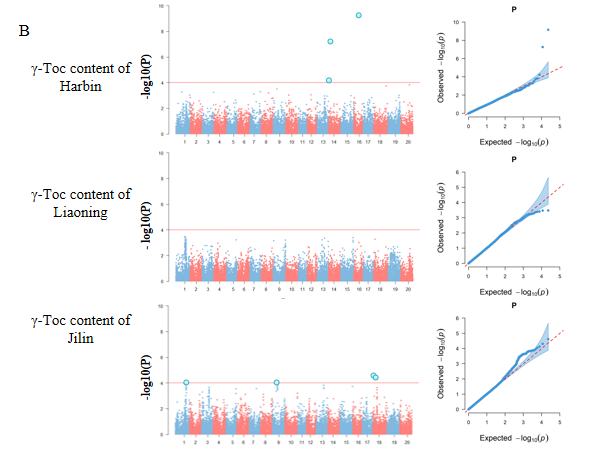


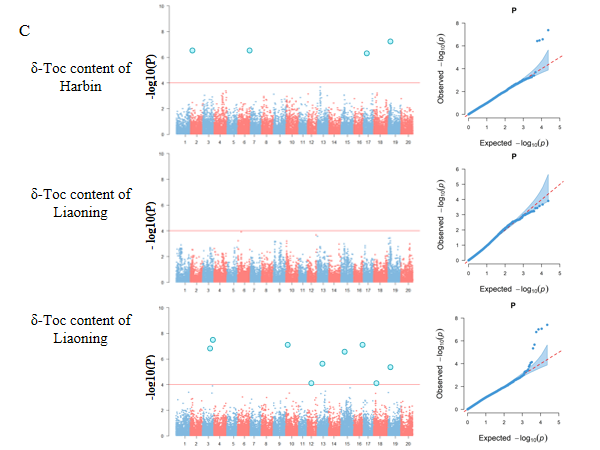

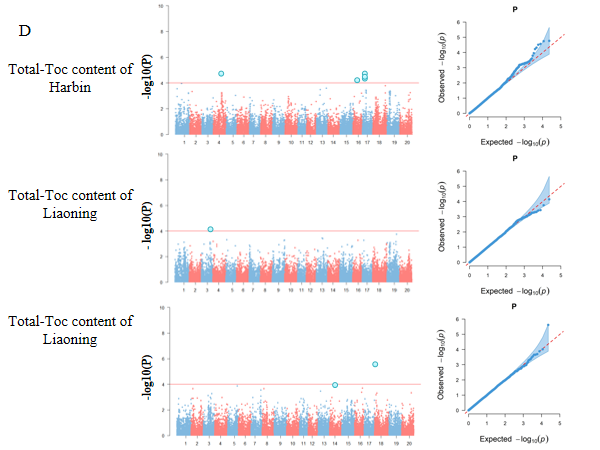


**Figure S4** Manhattan plot and Q-Q plot generated from GWAS for tocopherol content in soybean seeds among 175 soybean accessions using BLINK model.

Note: A–D respectively represent α-Toc, δ-Toc, γ-Toc, and Total-Toc content in three environments.


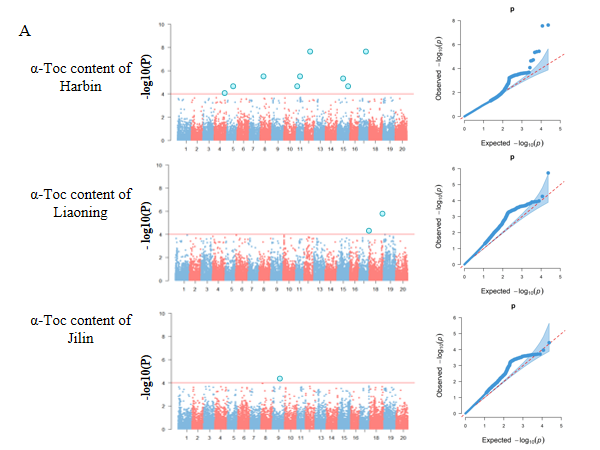

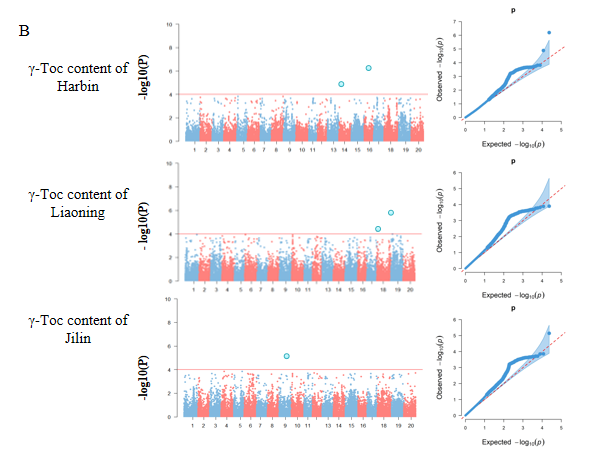

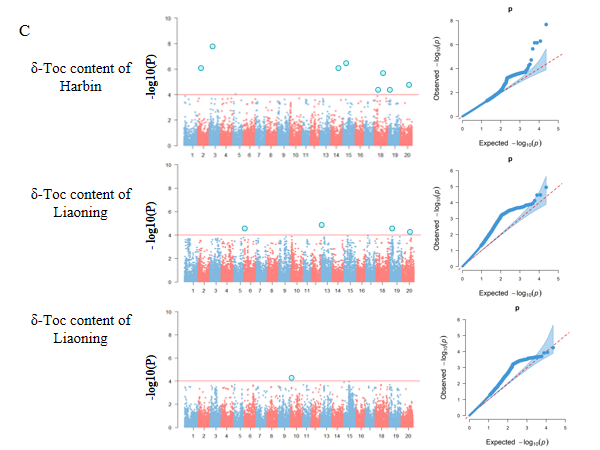

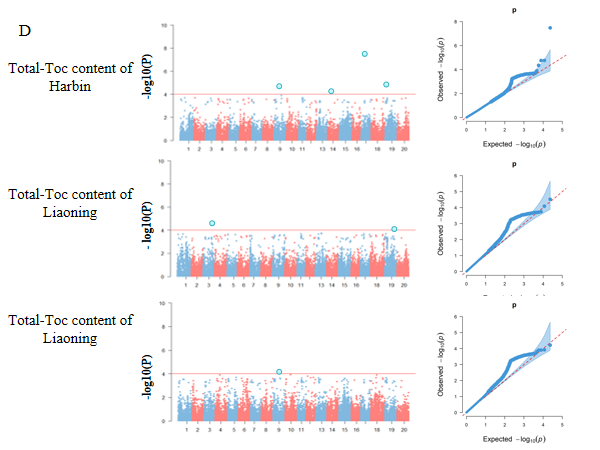


**Figure S5** Manhattan plot and Q-Q plot generated from GWAS for tocopherol content in soybean seeds among 175 soybean accessions using FarmCPU model.

Note: A–D respectively represent α-Toc, δ-Toc, γ-Toc, and Total-Toc content in three environments.


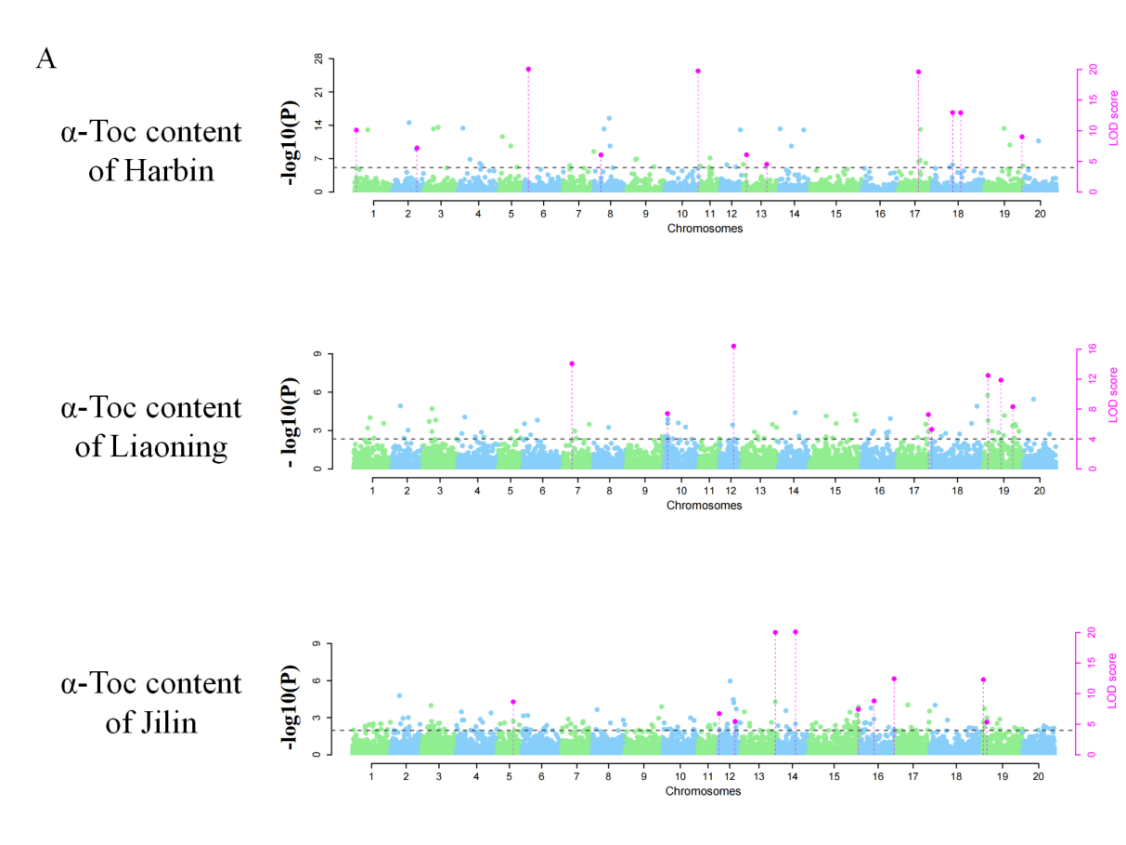

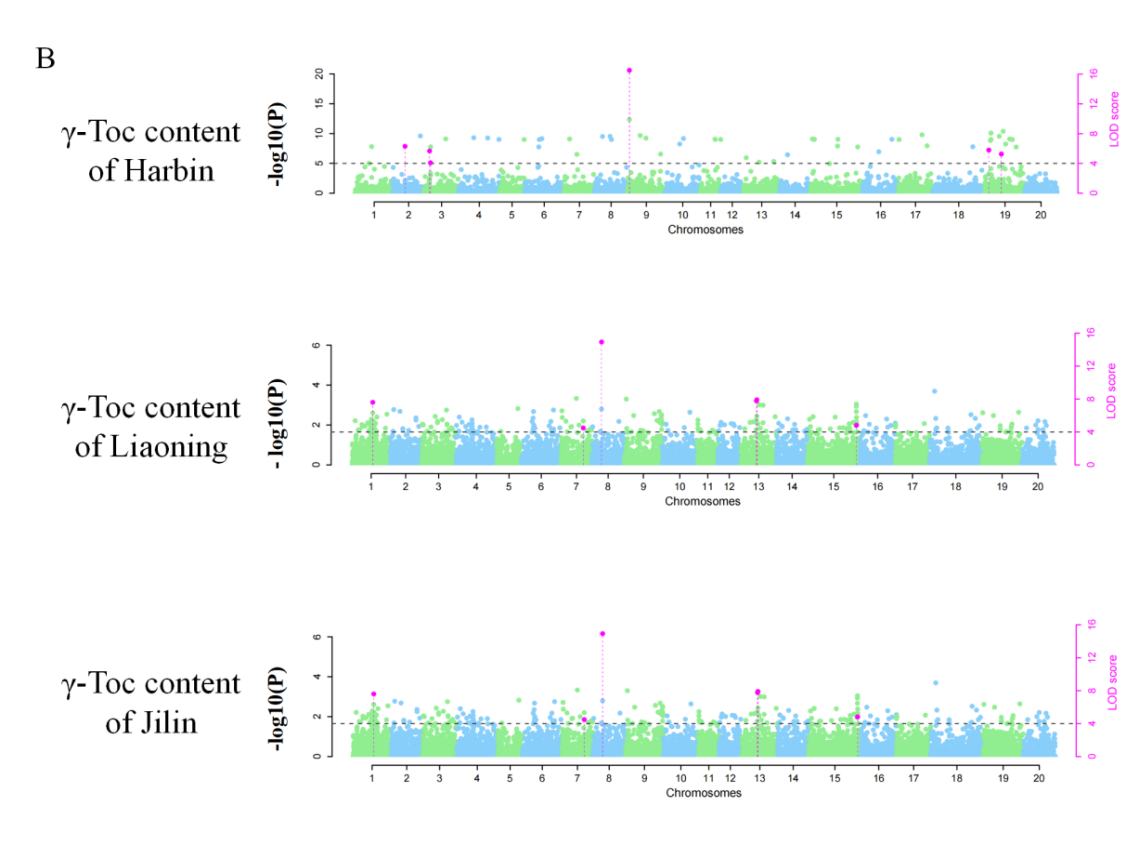


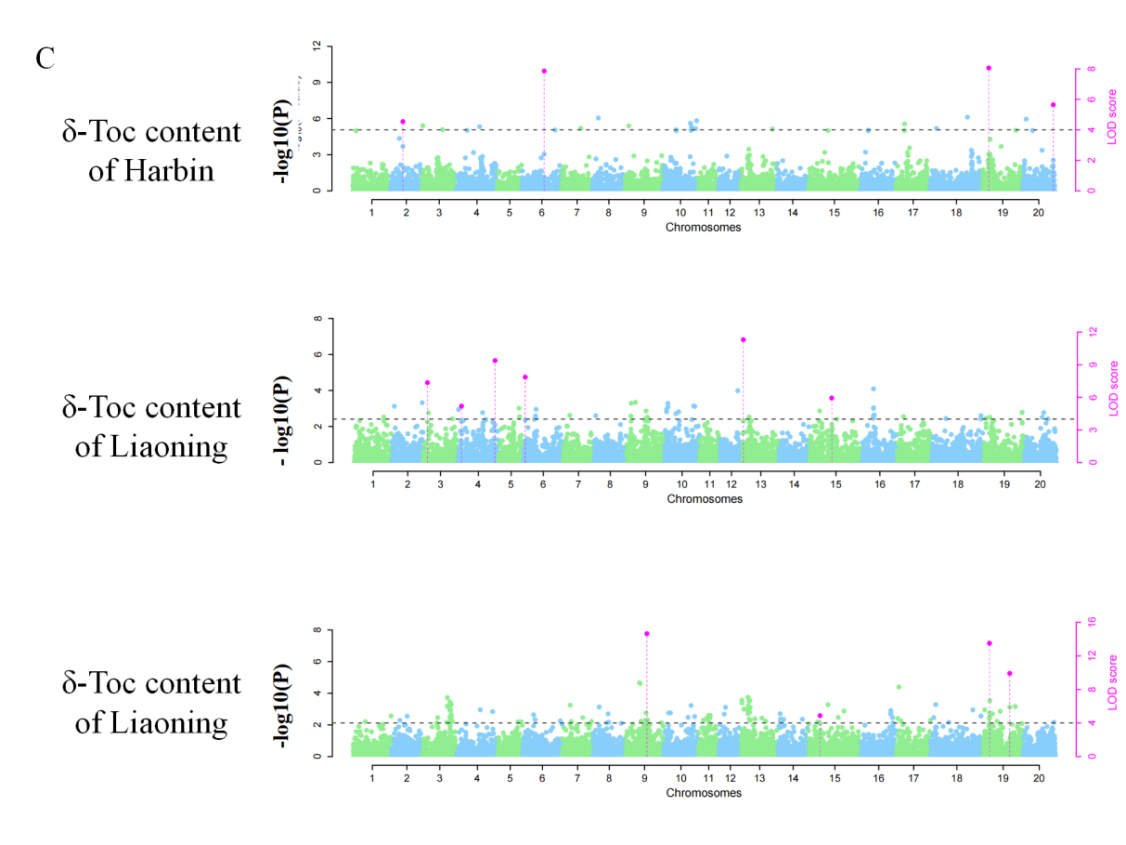


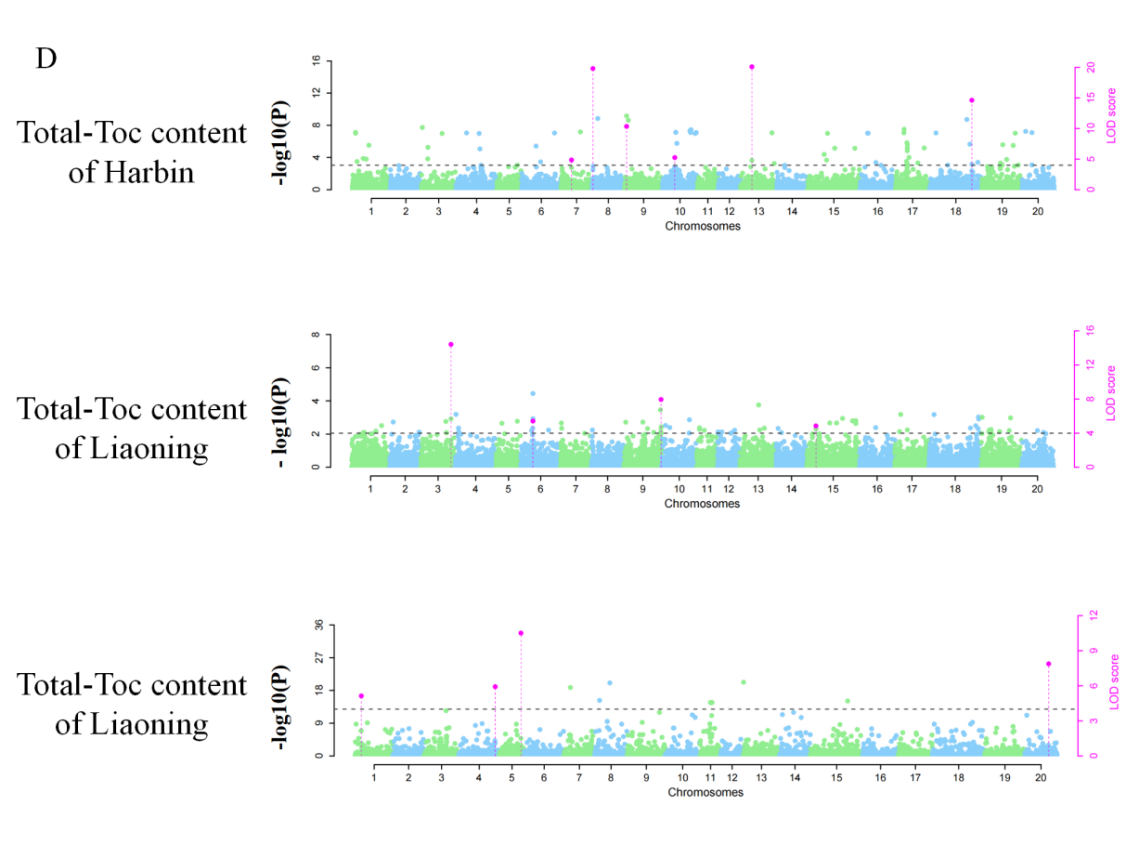


**Figure S6** Manhattan plot and Q-Q plot generated from GWAS for tocopherol content in soybean seeds among 175 soybean accessions using the 3VmrMLM model single-environment method.

Note: A–D respectively represent α-Toc, δ-Toc, γ-Toc, and Total-Toc content in three environments.


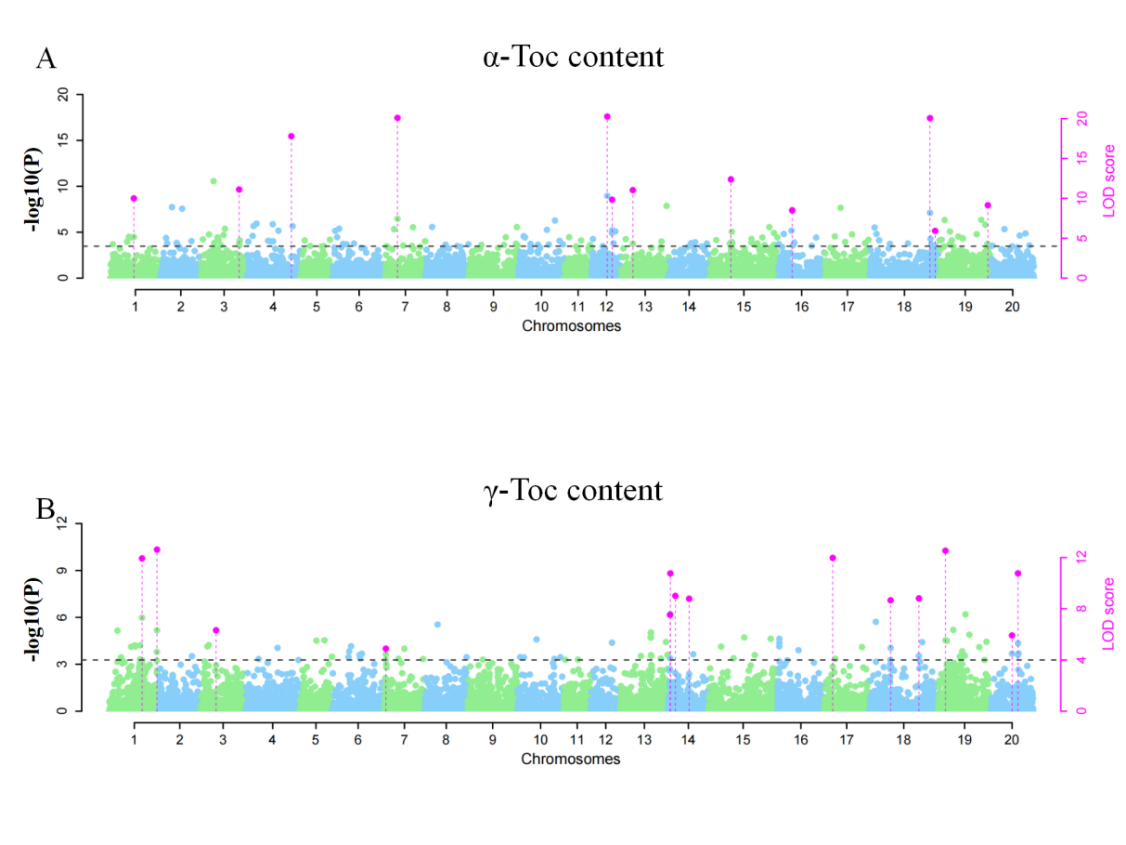

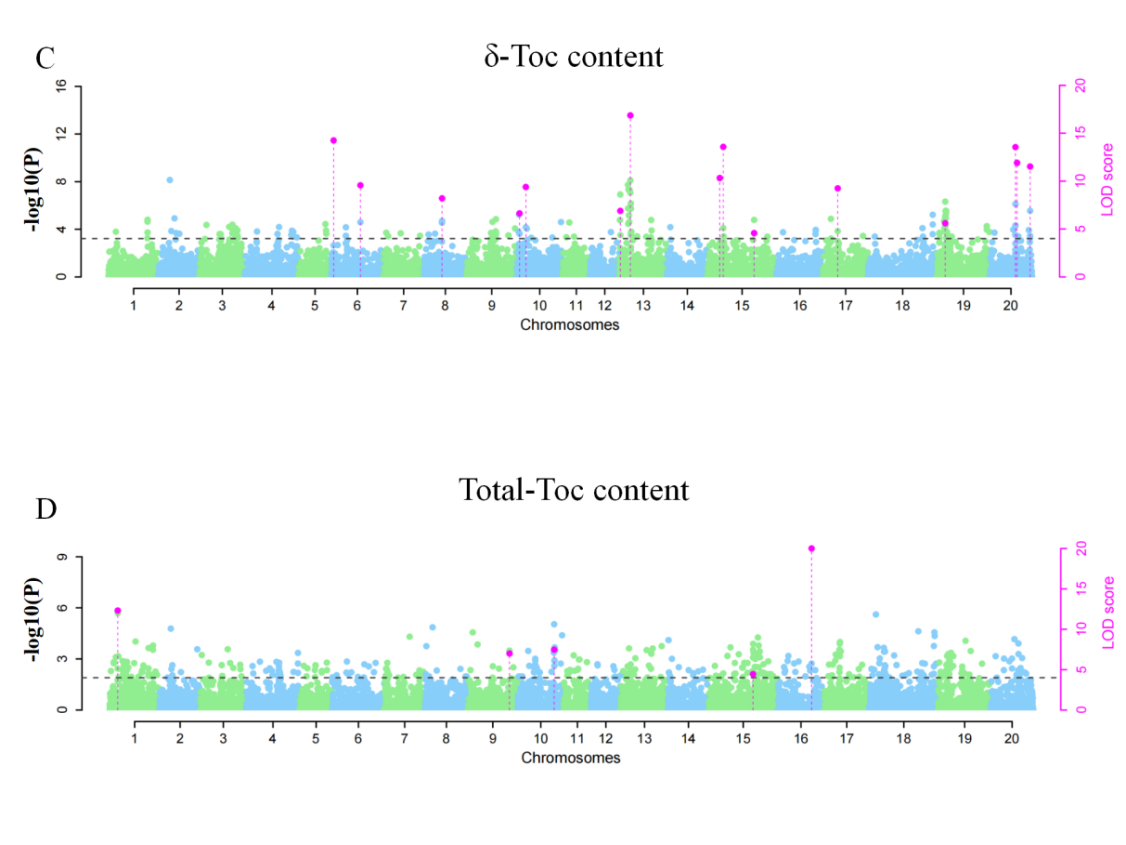


**Figure S7** Manhattan plot and Q-Q plot generated from GWAS for tocopherol content in soybean seeds among 175 soybean accessions using QTL detection of 3VmrMLM multiple-environment method.

Note: A–D respectively represent α-Toc, δ-Toc, γ-Toc, and Total-Toc content in three environments.


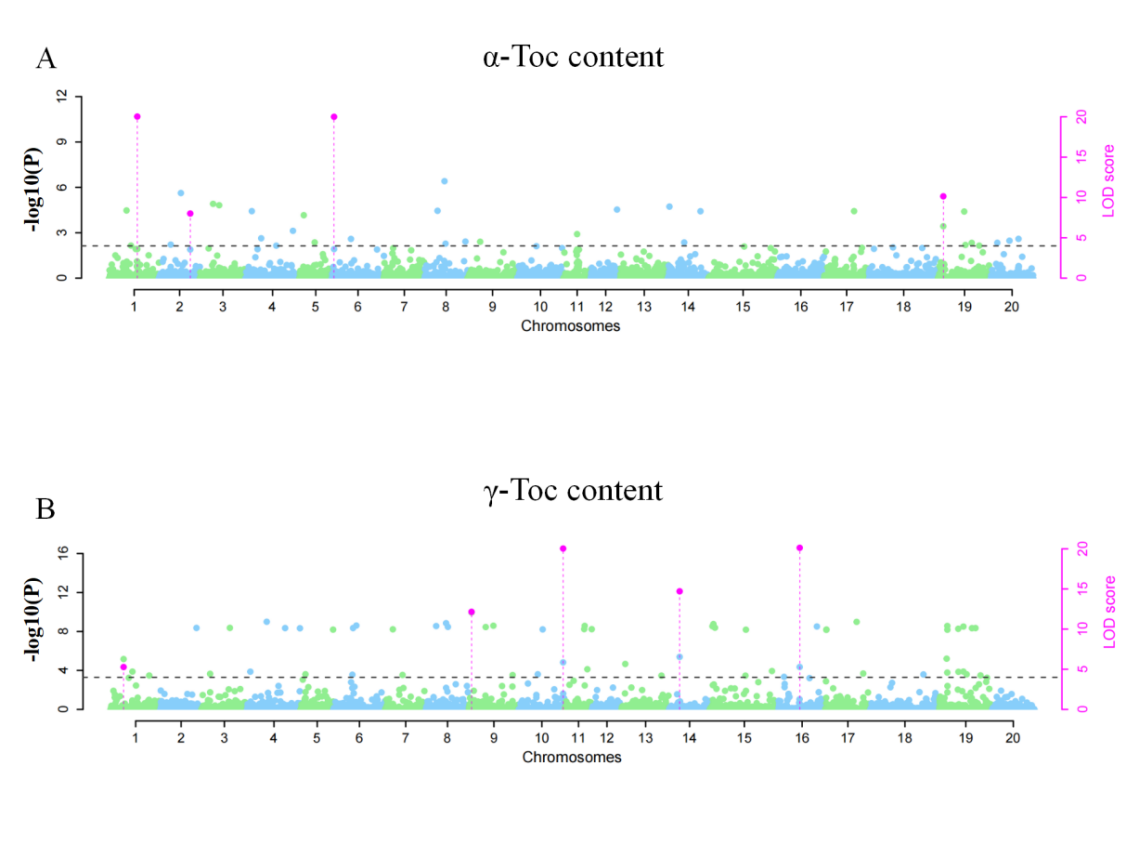

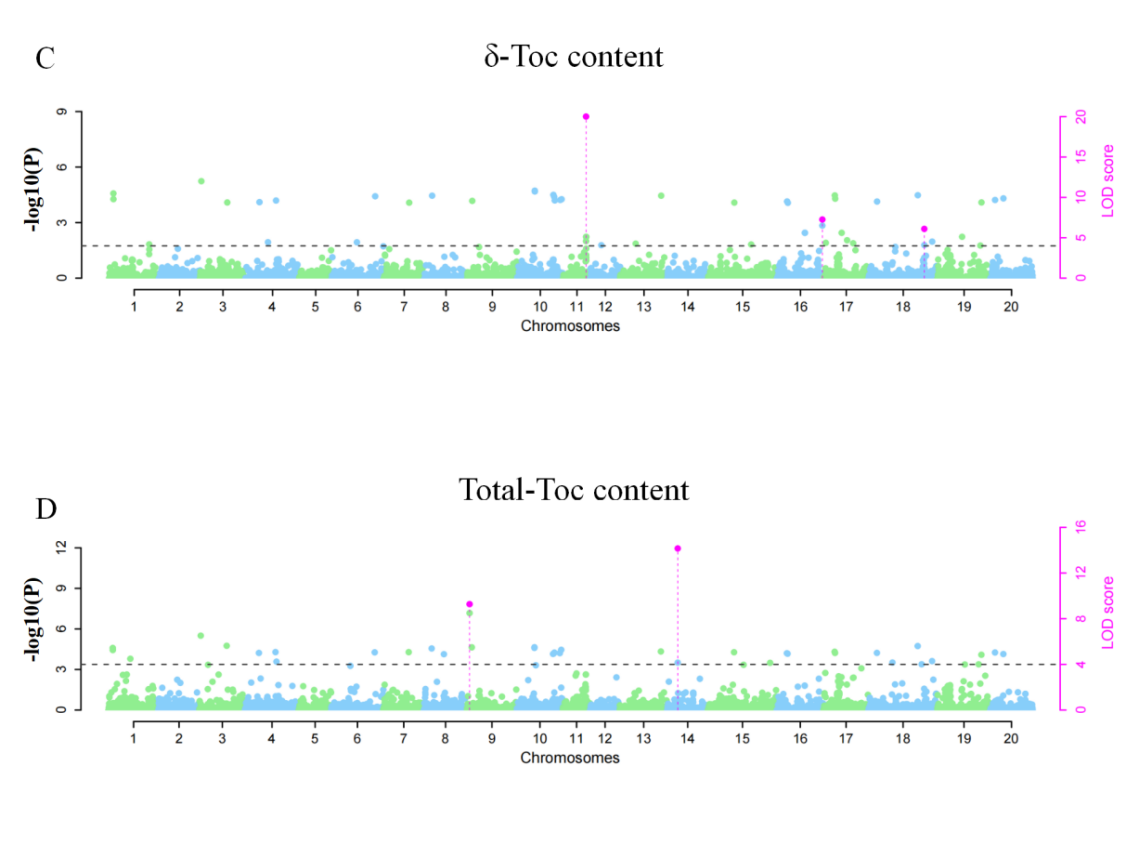


**Figure S8** Manhattan plot and Q-Q plot generated from GWAS for tocopherol content in soybean seeds among 175 soybean accessions using QEI detection of 3VmrMLM multiple-environment method.

Note: A–D respectively represent α-Toc, δ-Toc, γ-Toc, and Total-Toc content in three environments.


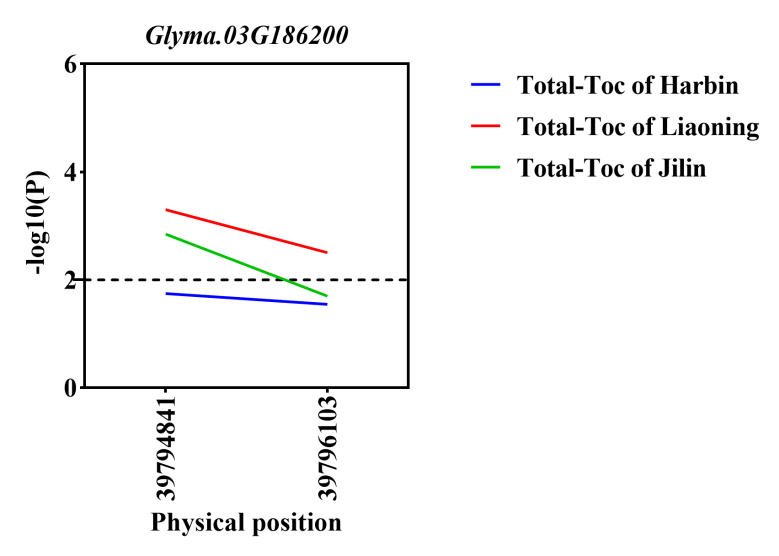


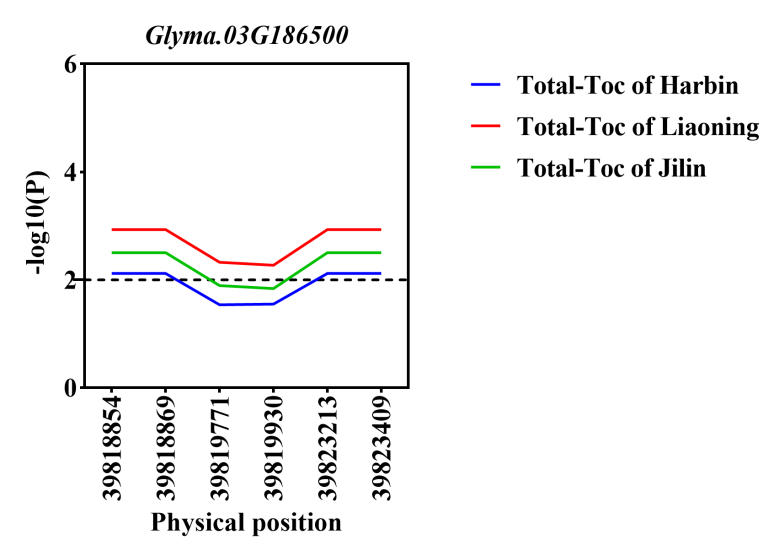


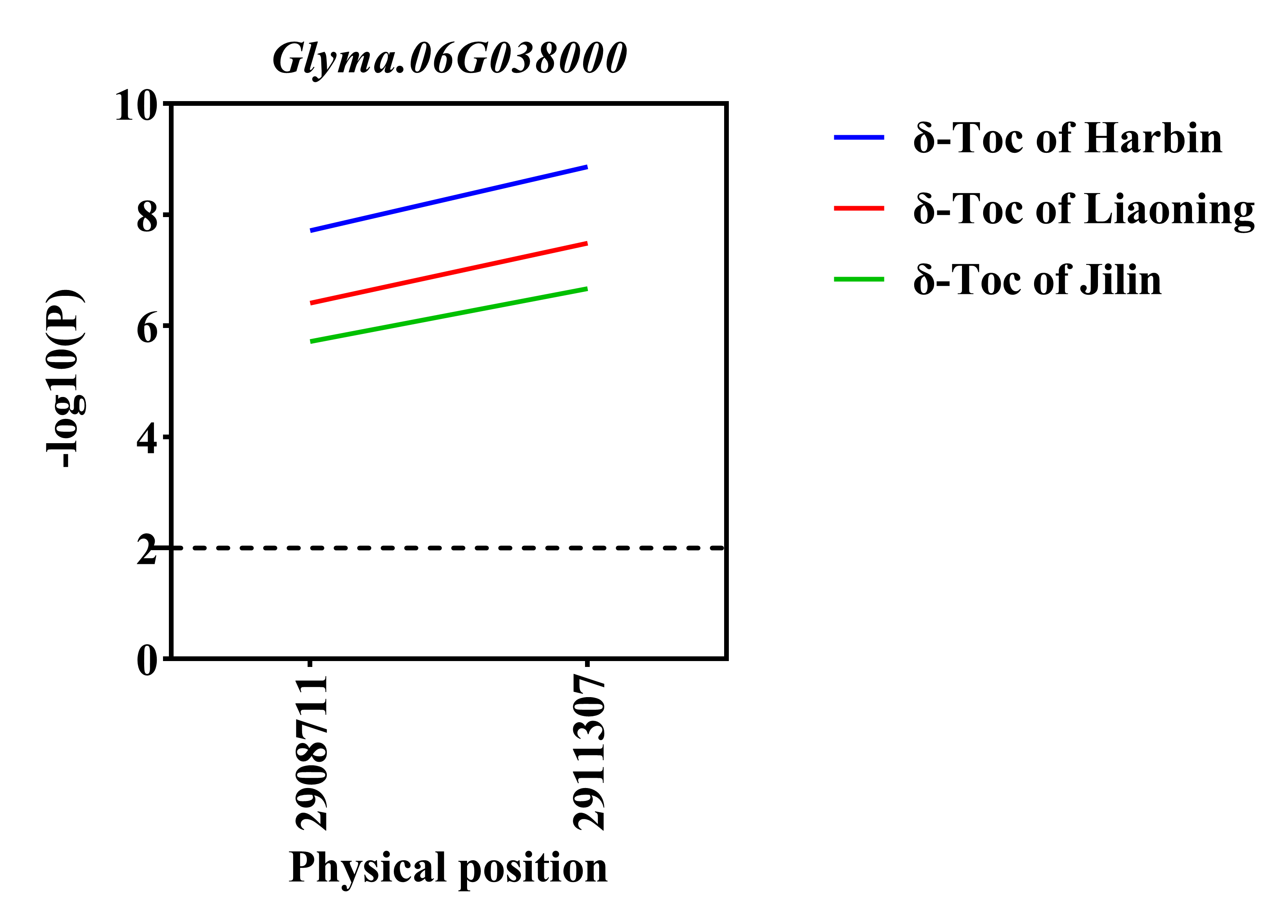


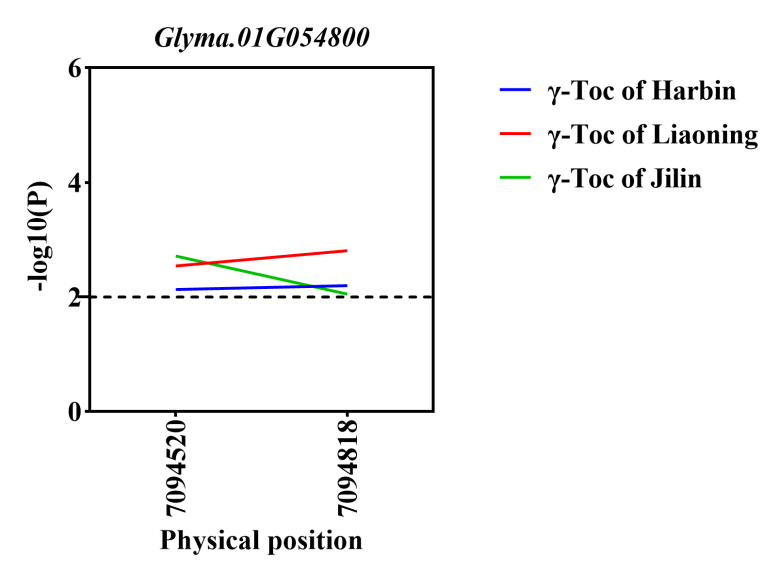


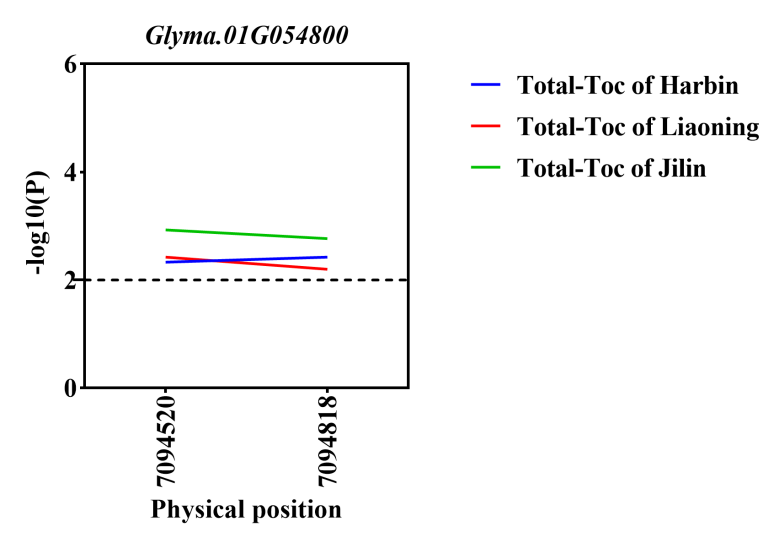


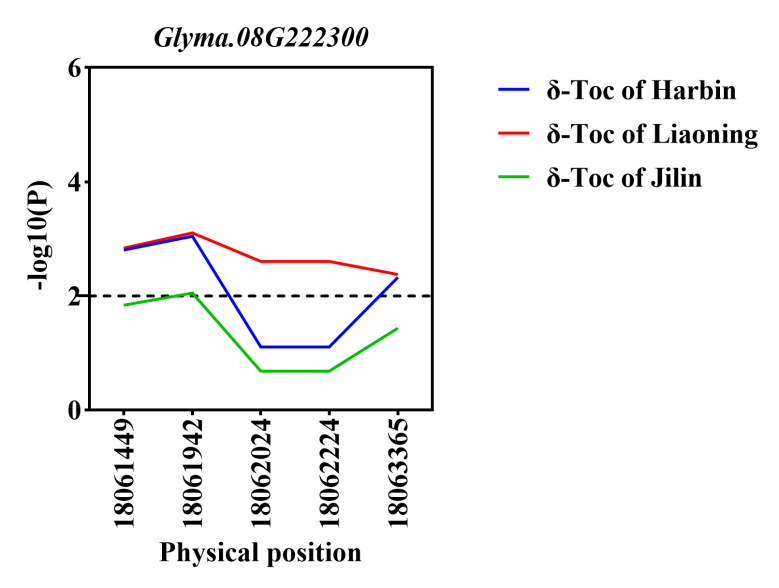


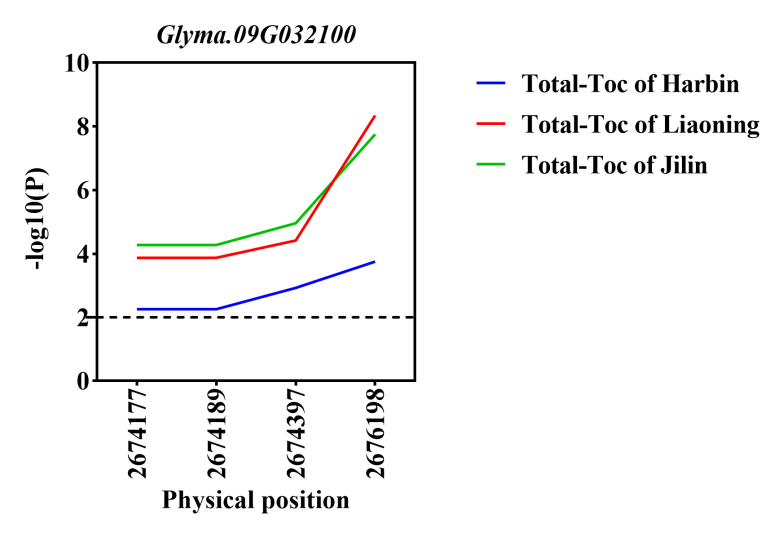


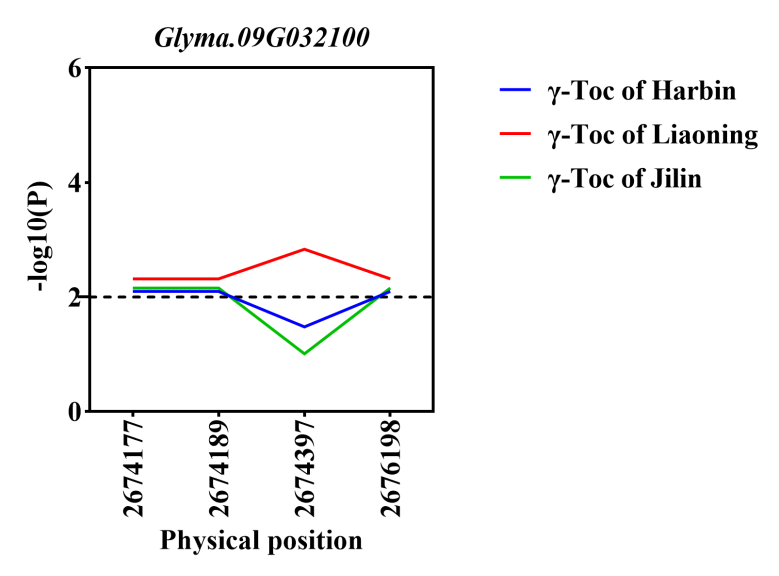


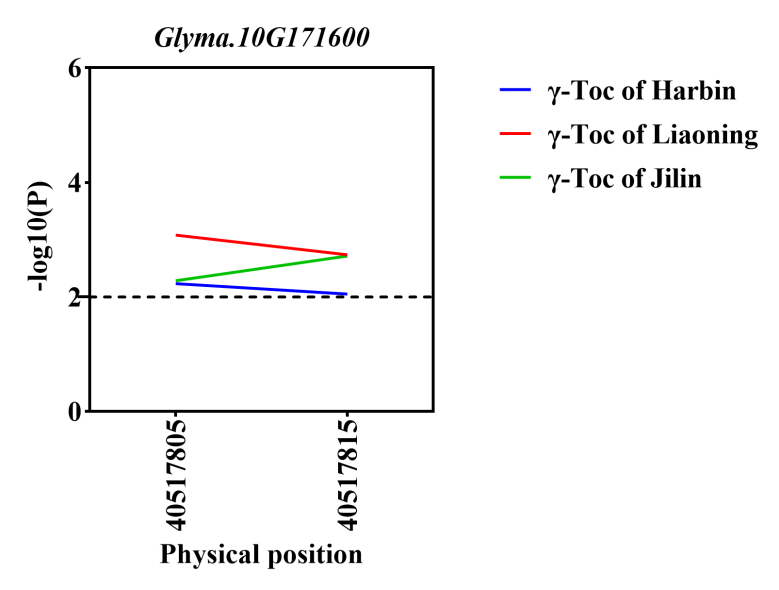


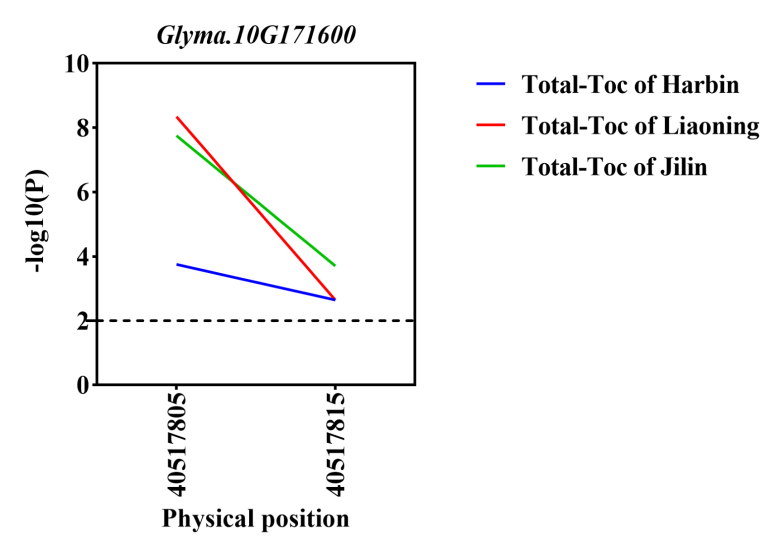


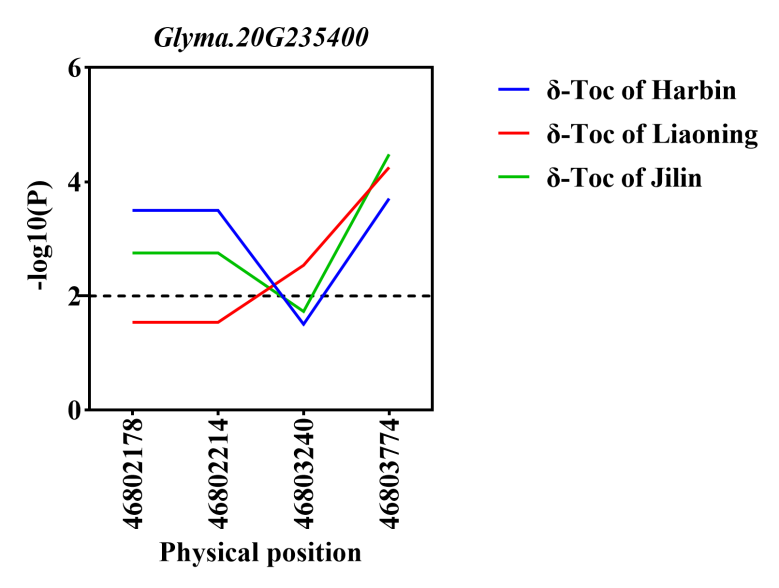


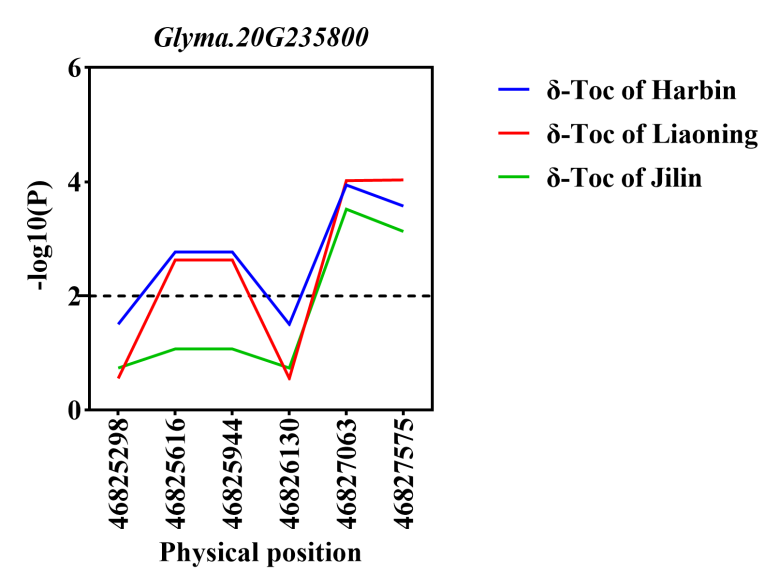


**Figure S9** Gene-based association analysis of candidate genes.

Note: The horizontal line indicates that the threshold was set to 2.0.


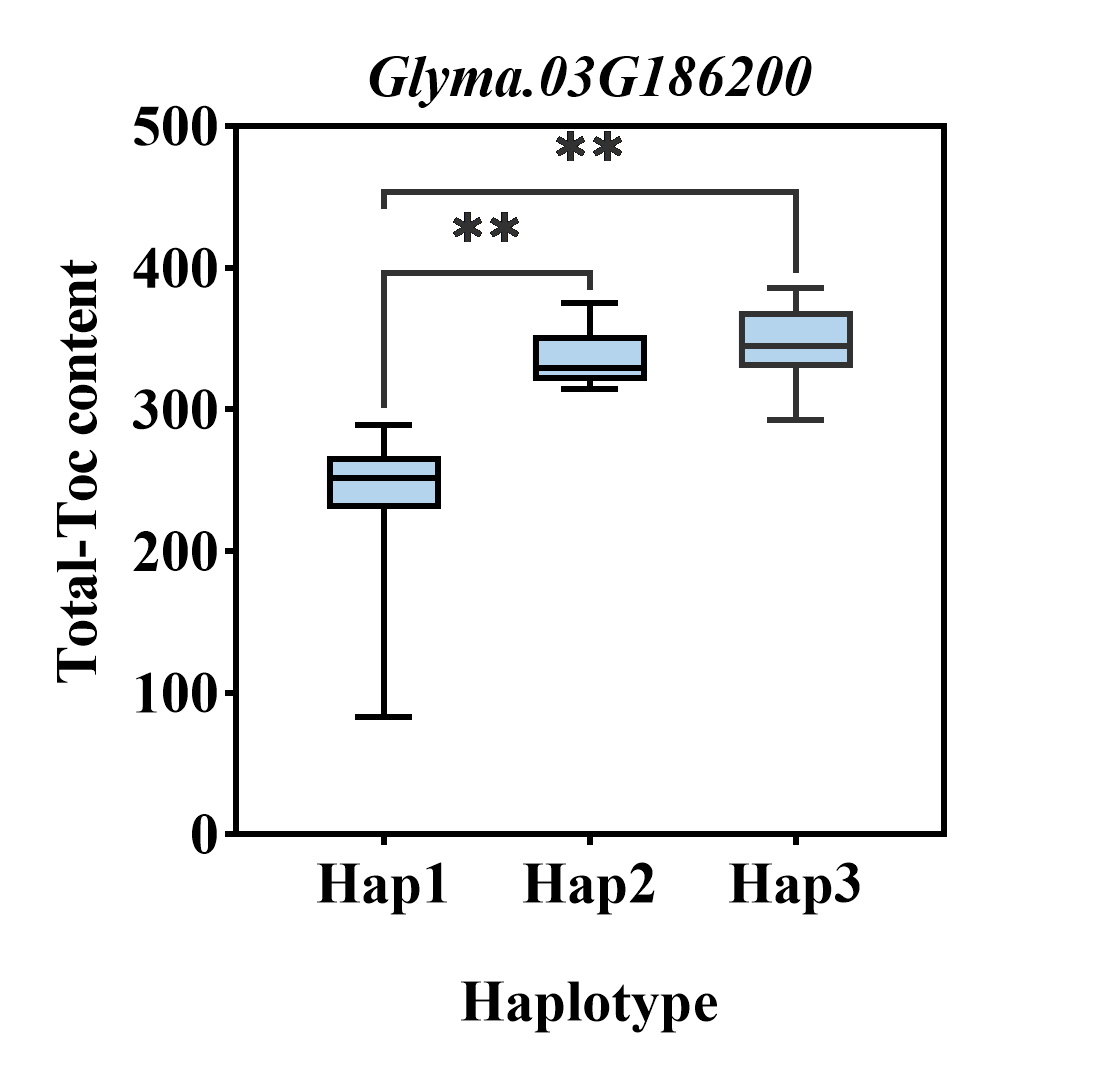


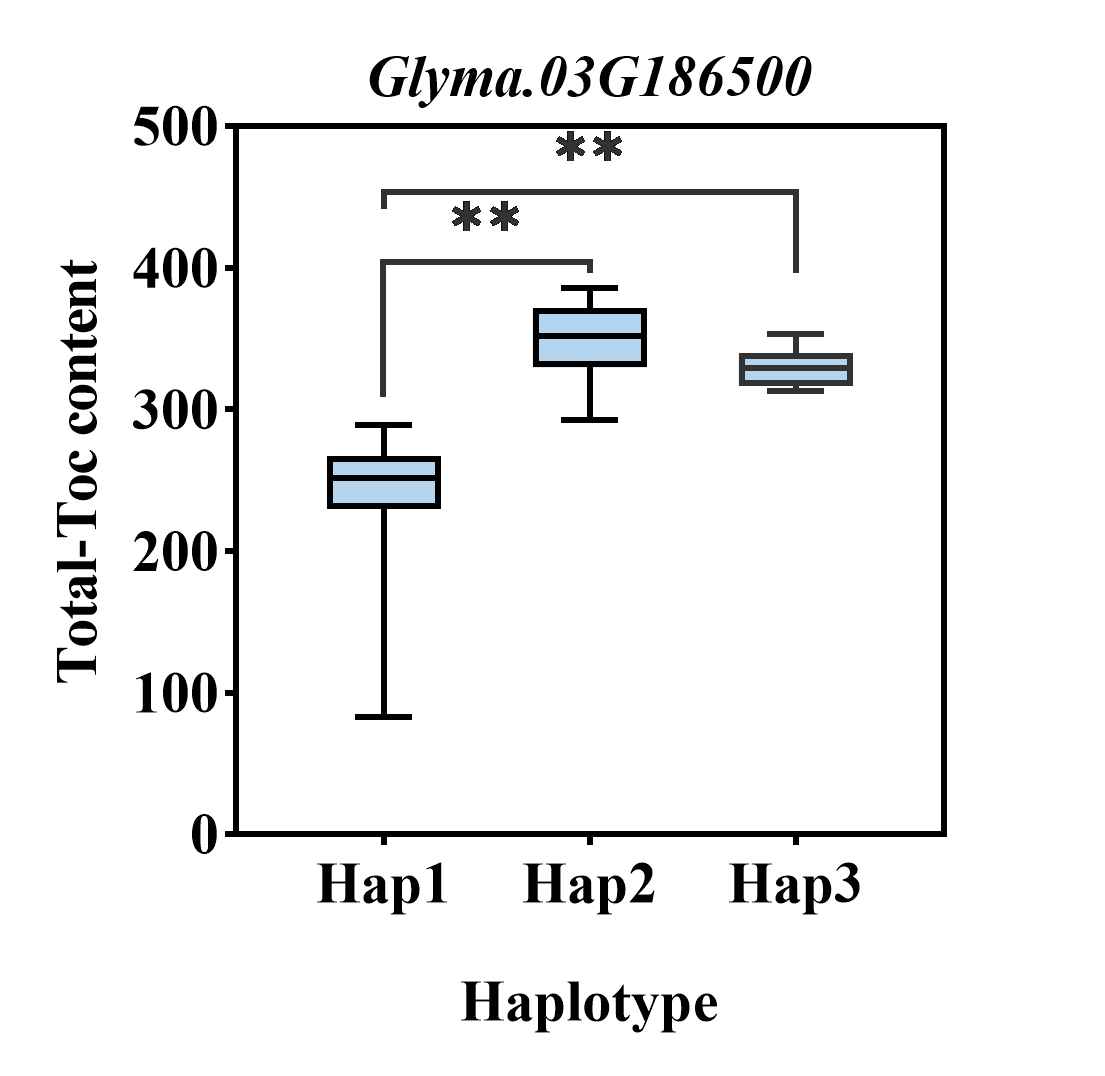


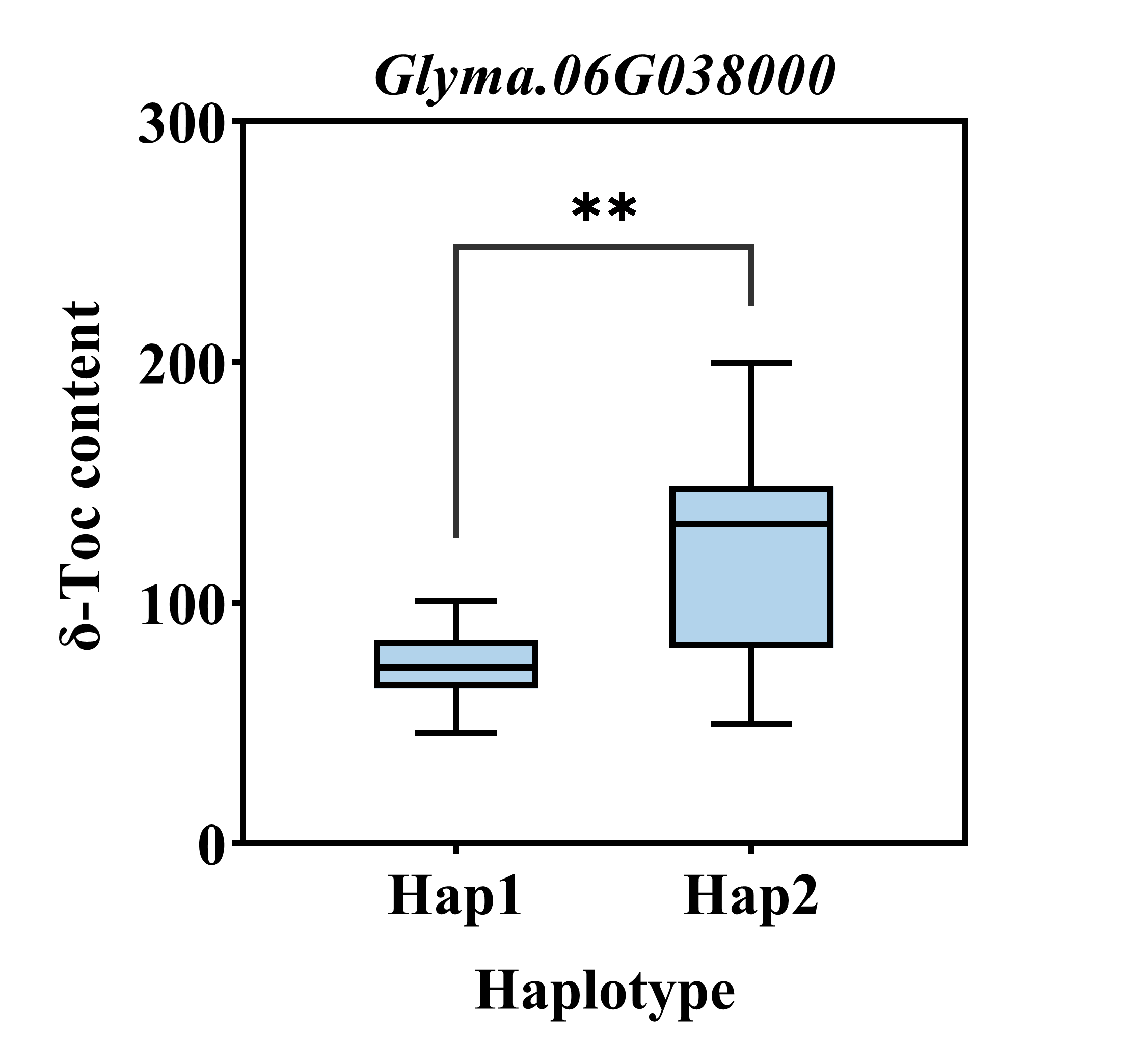


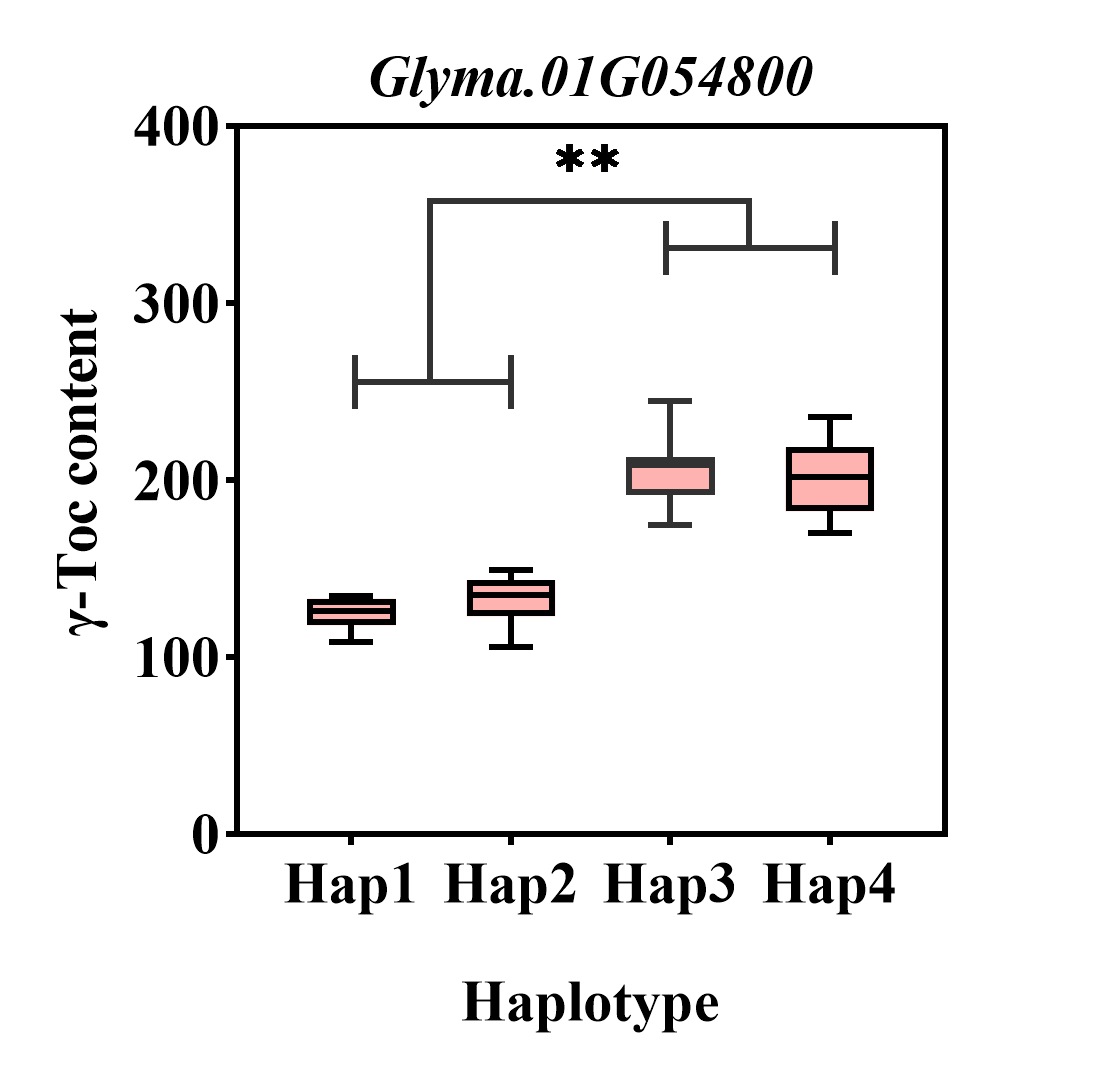


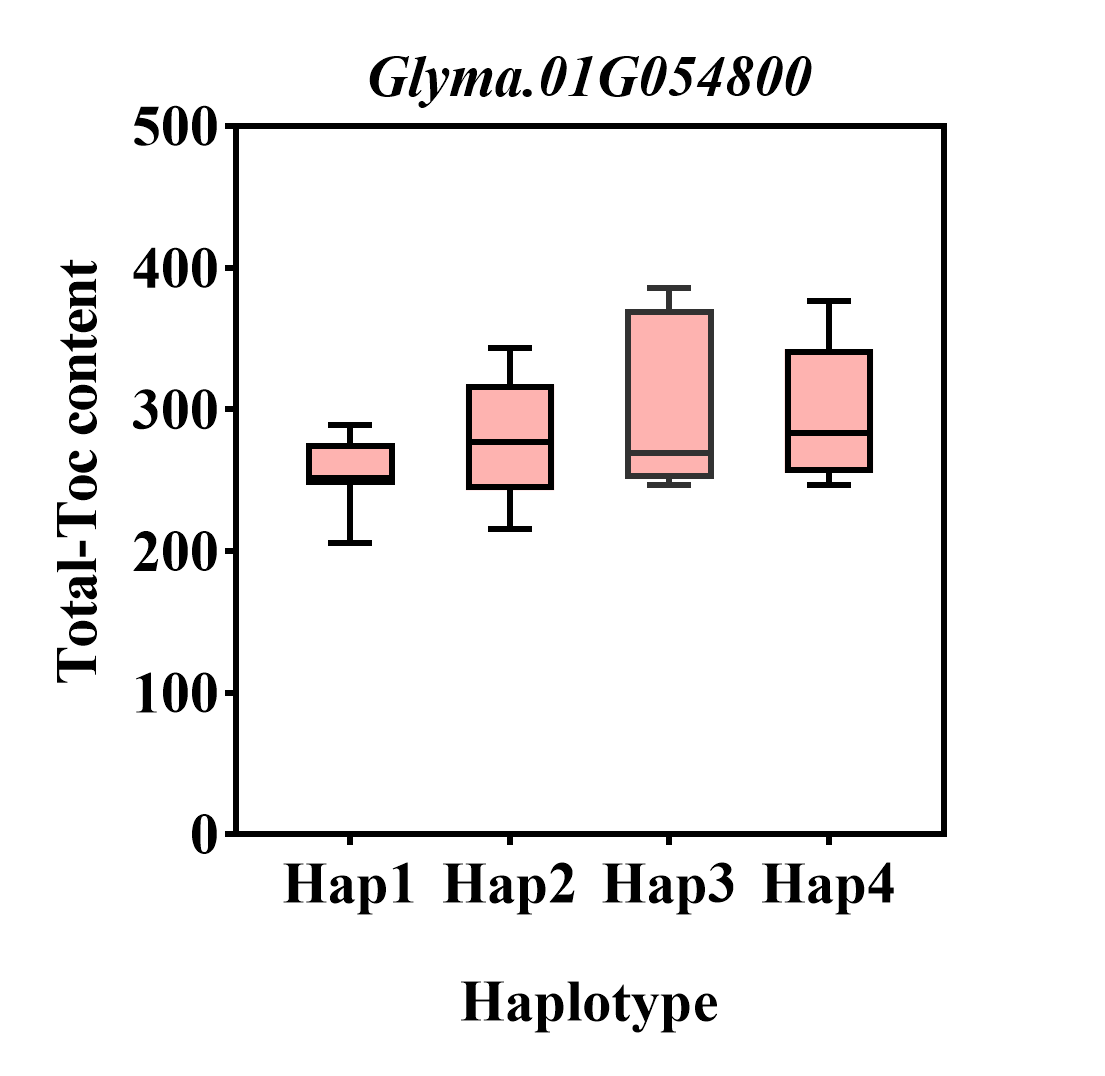


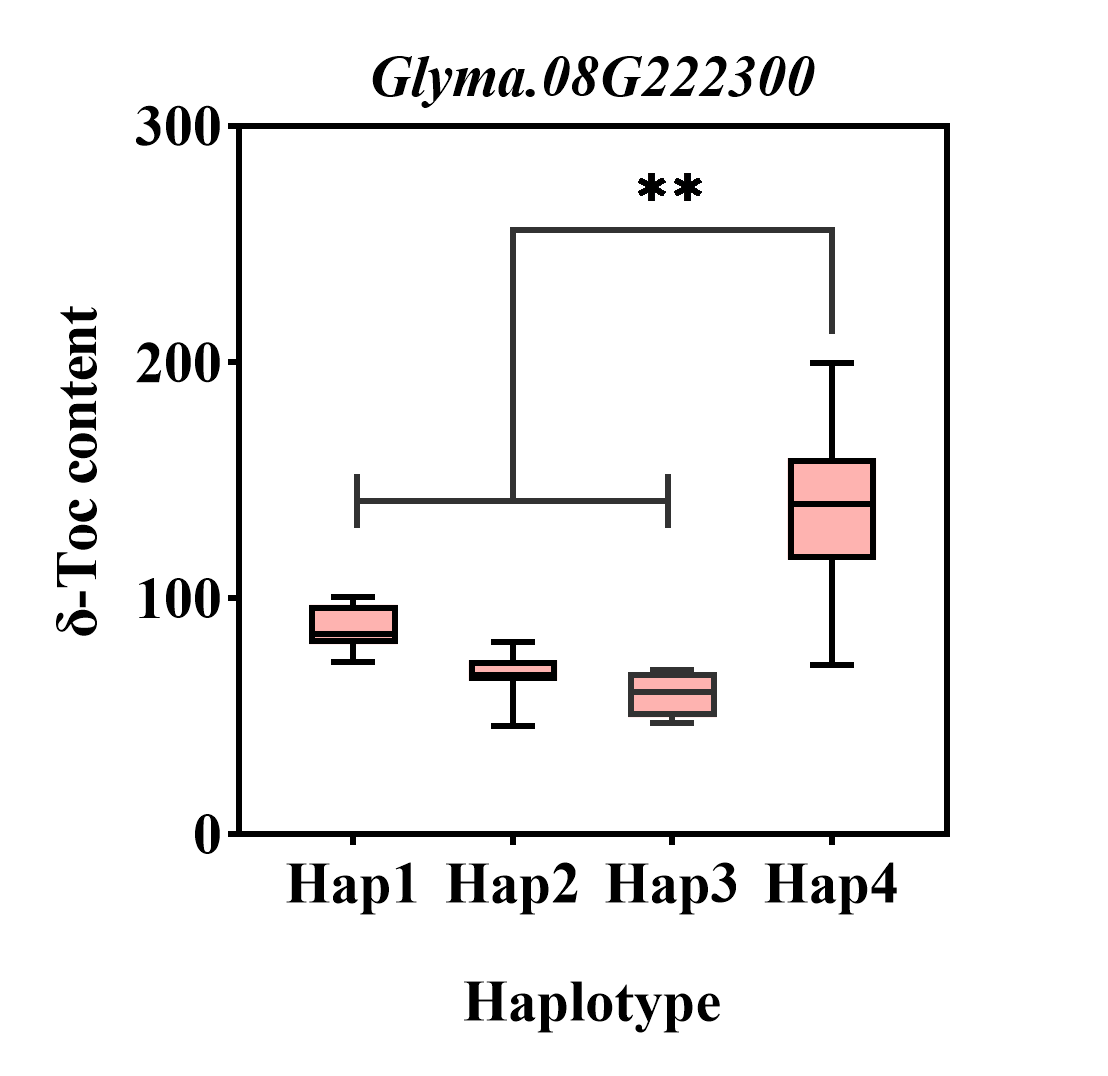


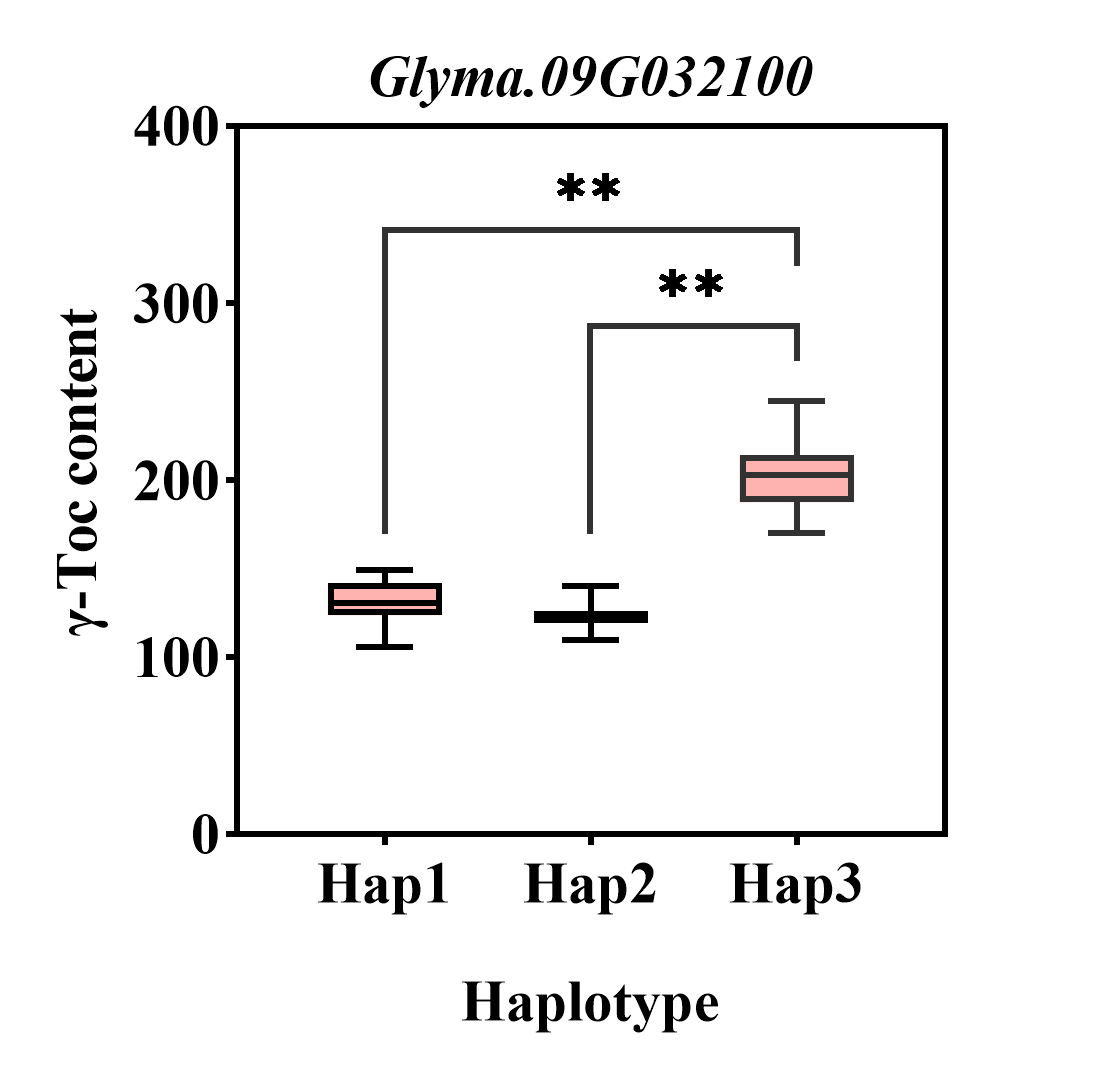


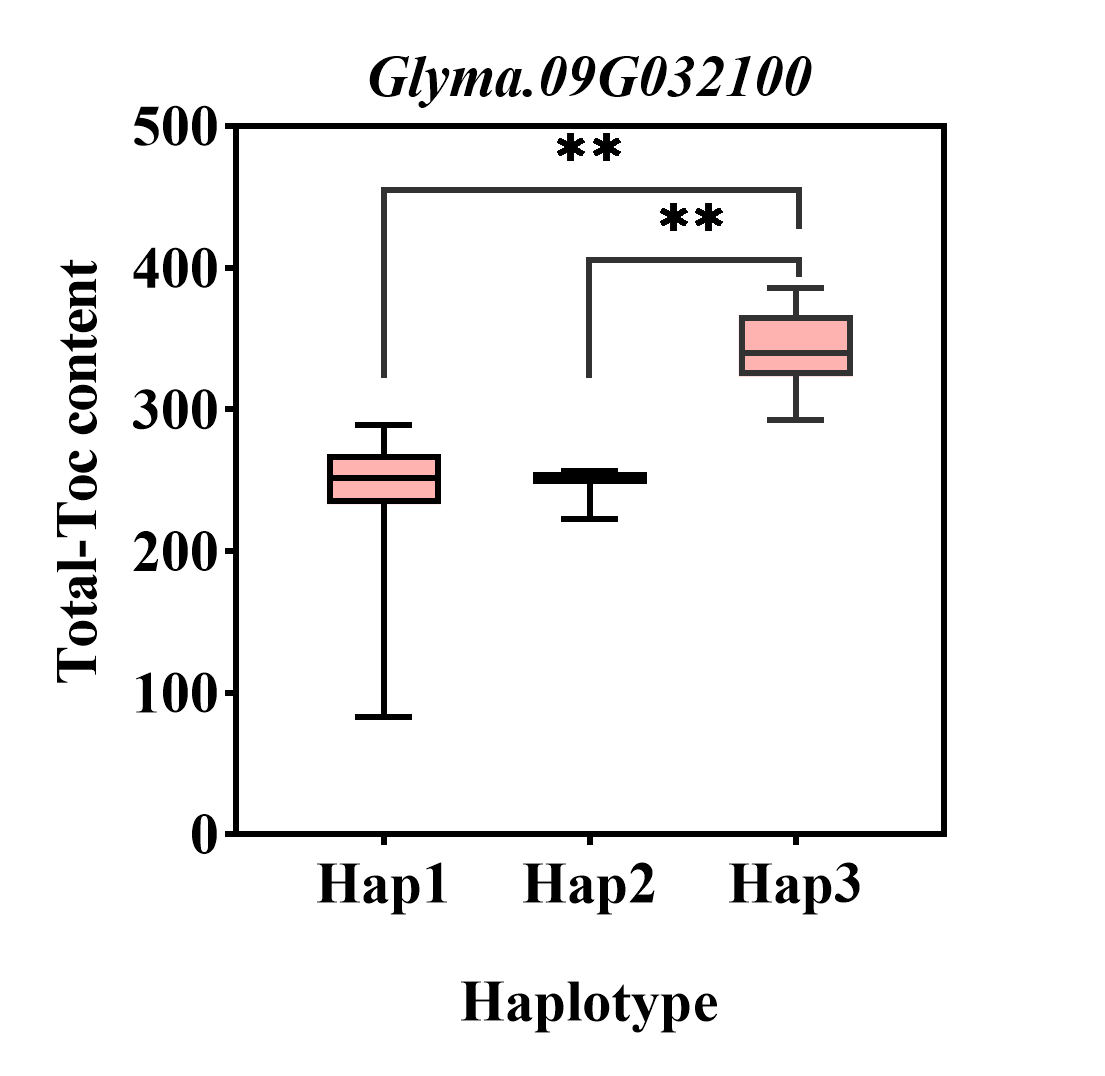


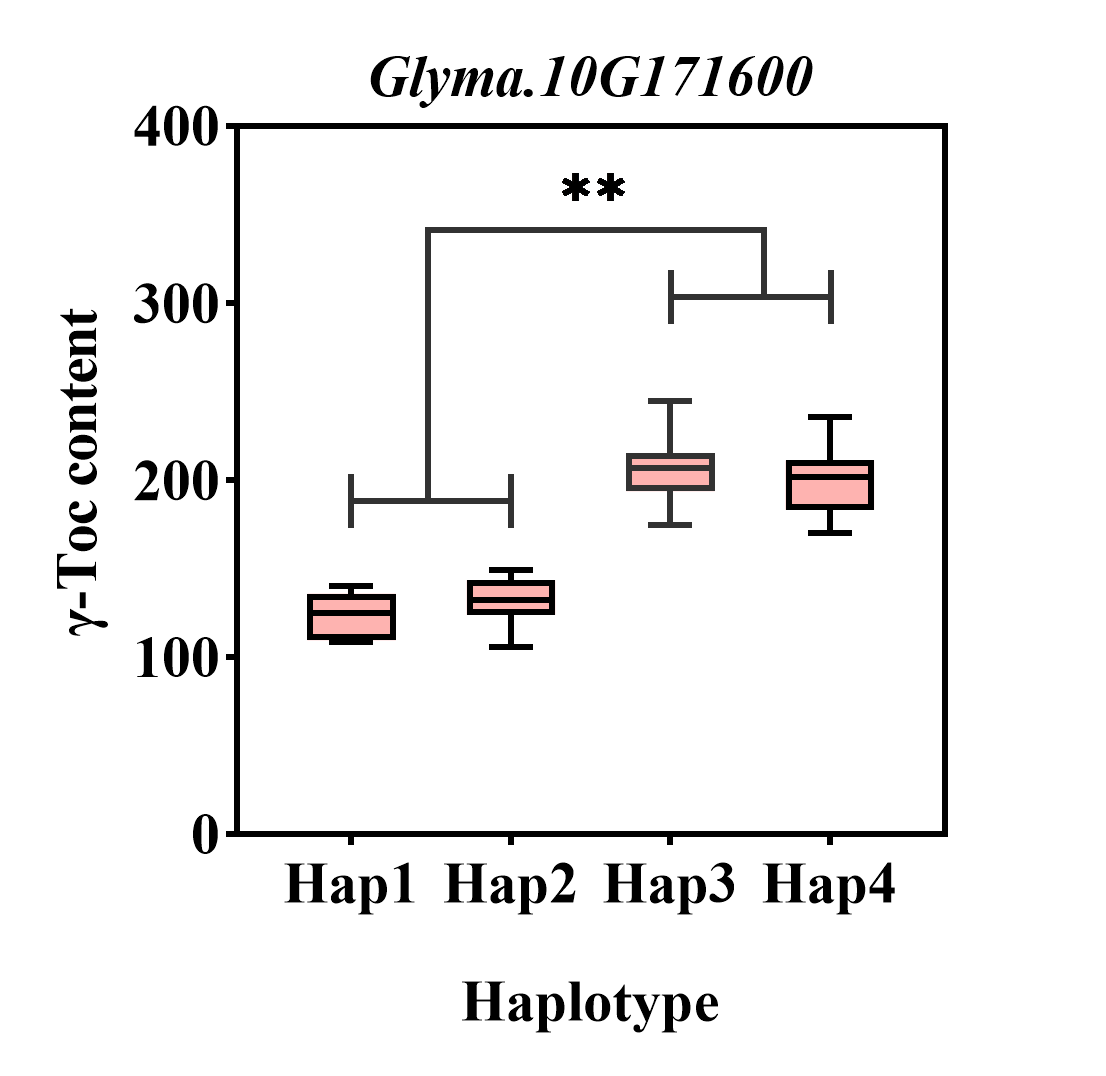


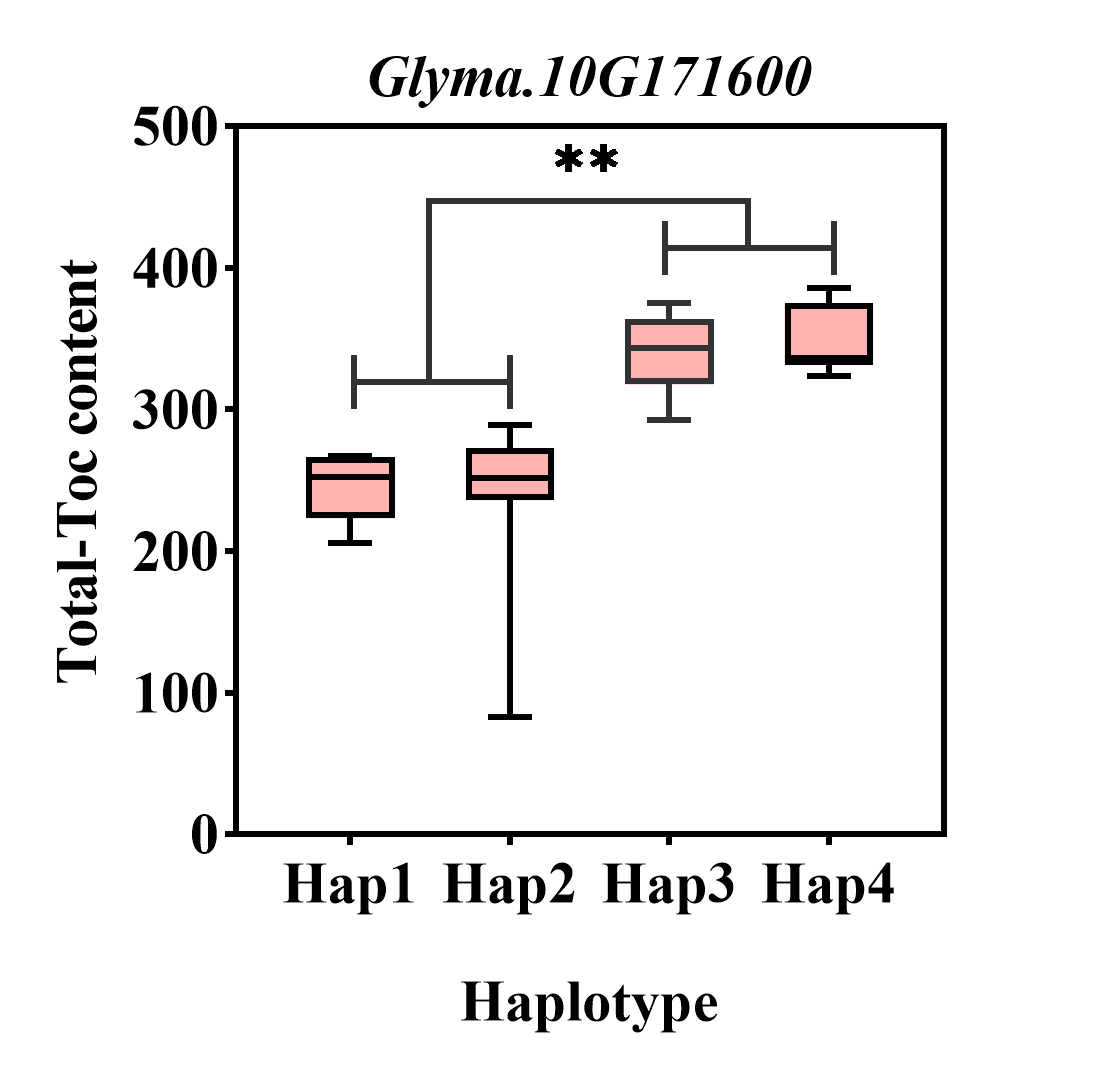


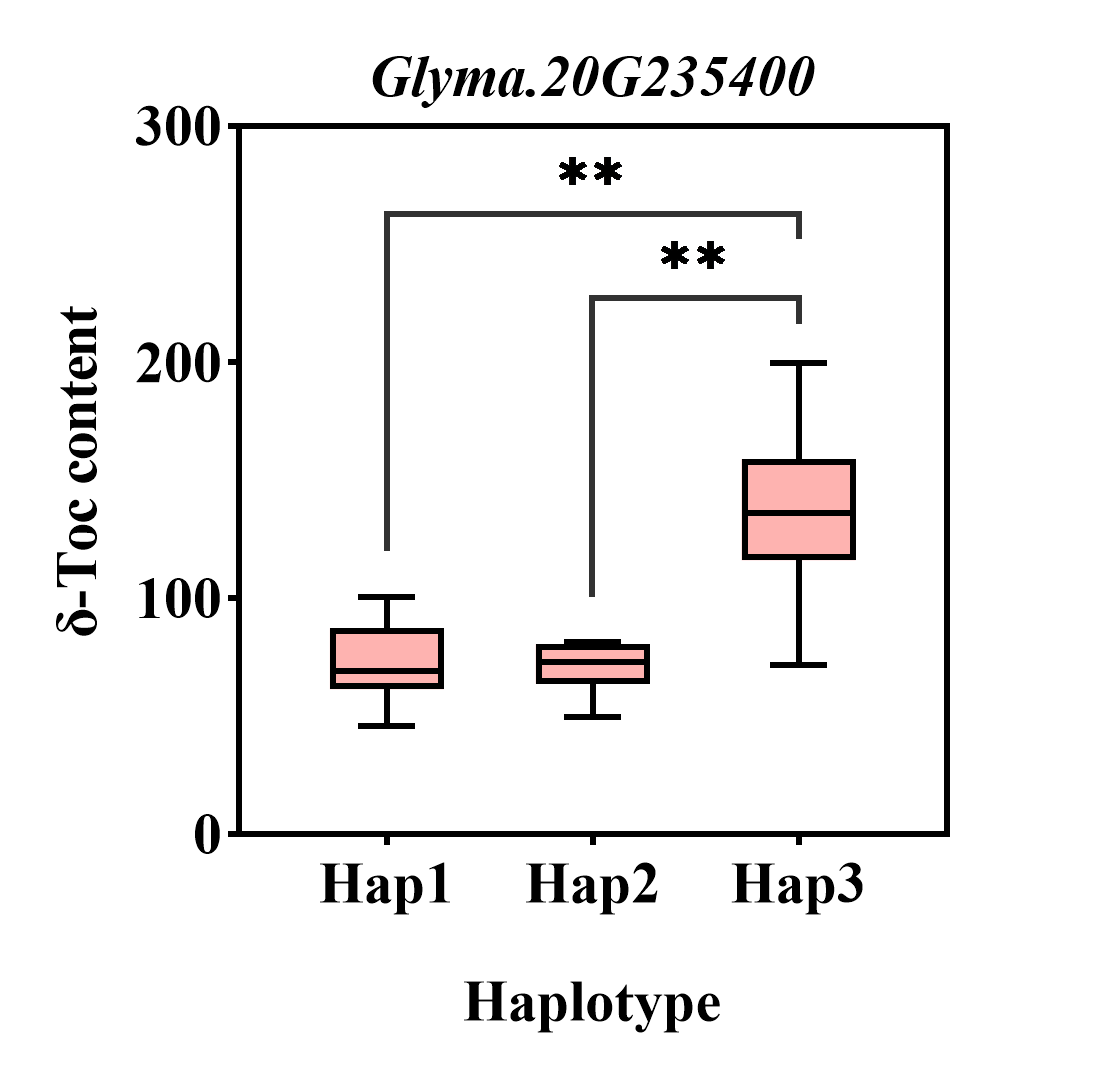


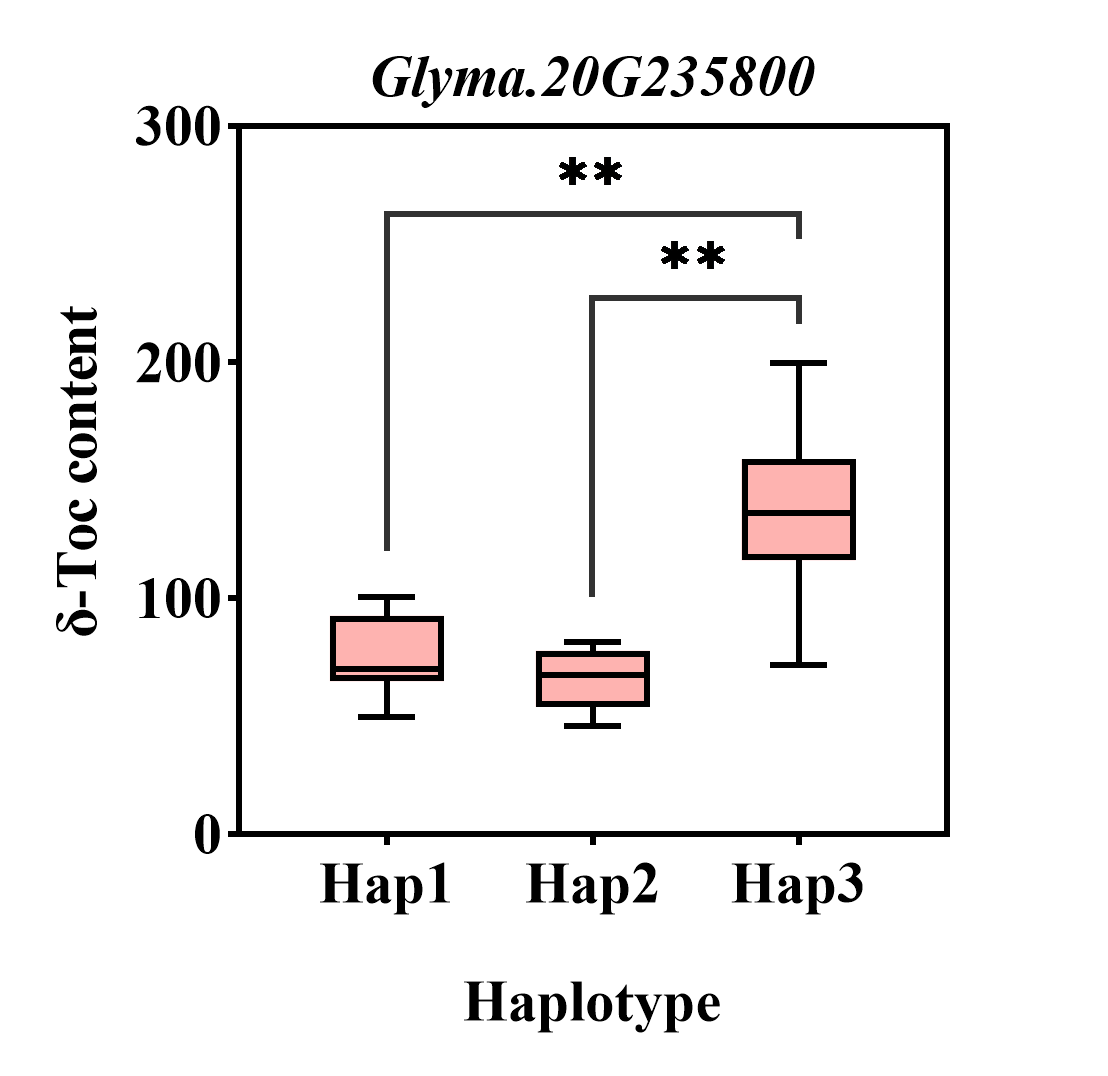


**Figure S10** Haplotypes analysis of candidate genes that related to Toc content.

Note: The * and ** was significance at *p* < 0.05 and *p* < 0.01, respectively.


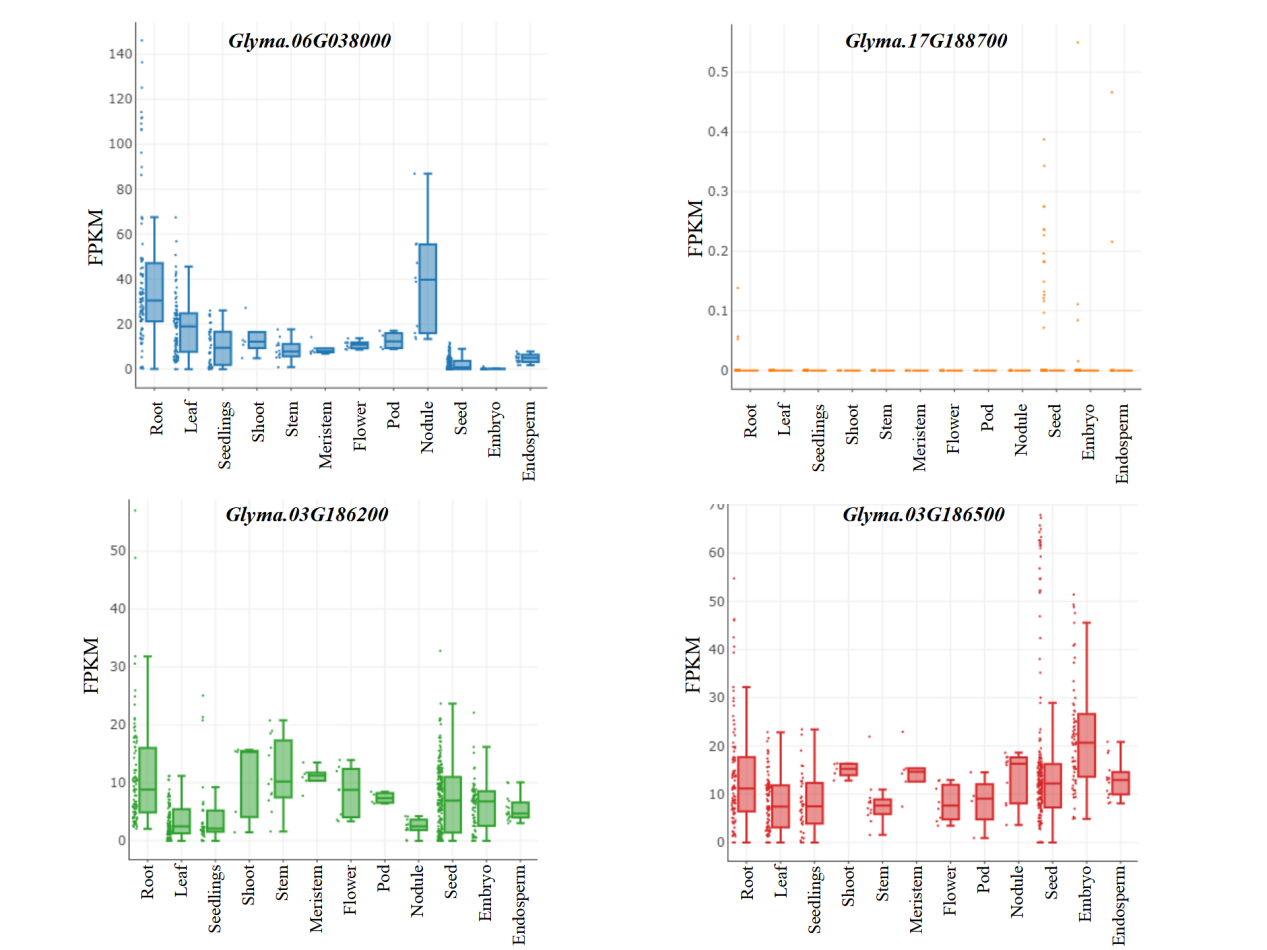


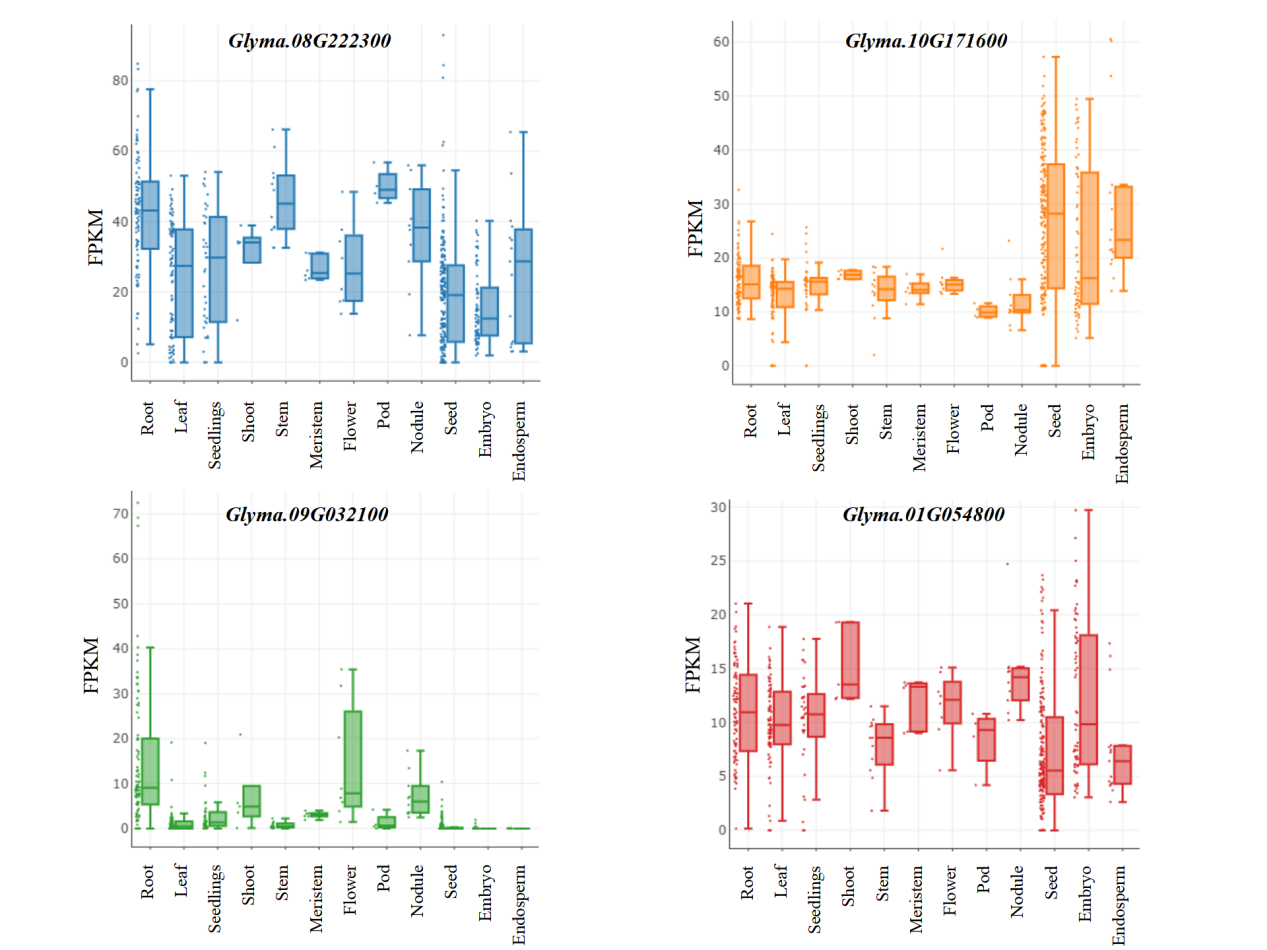


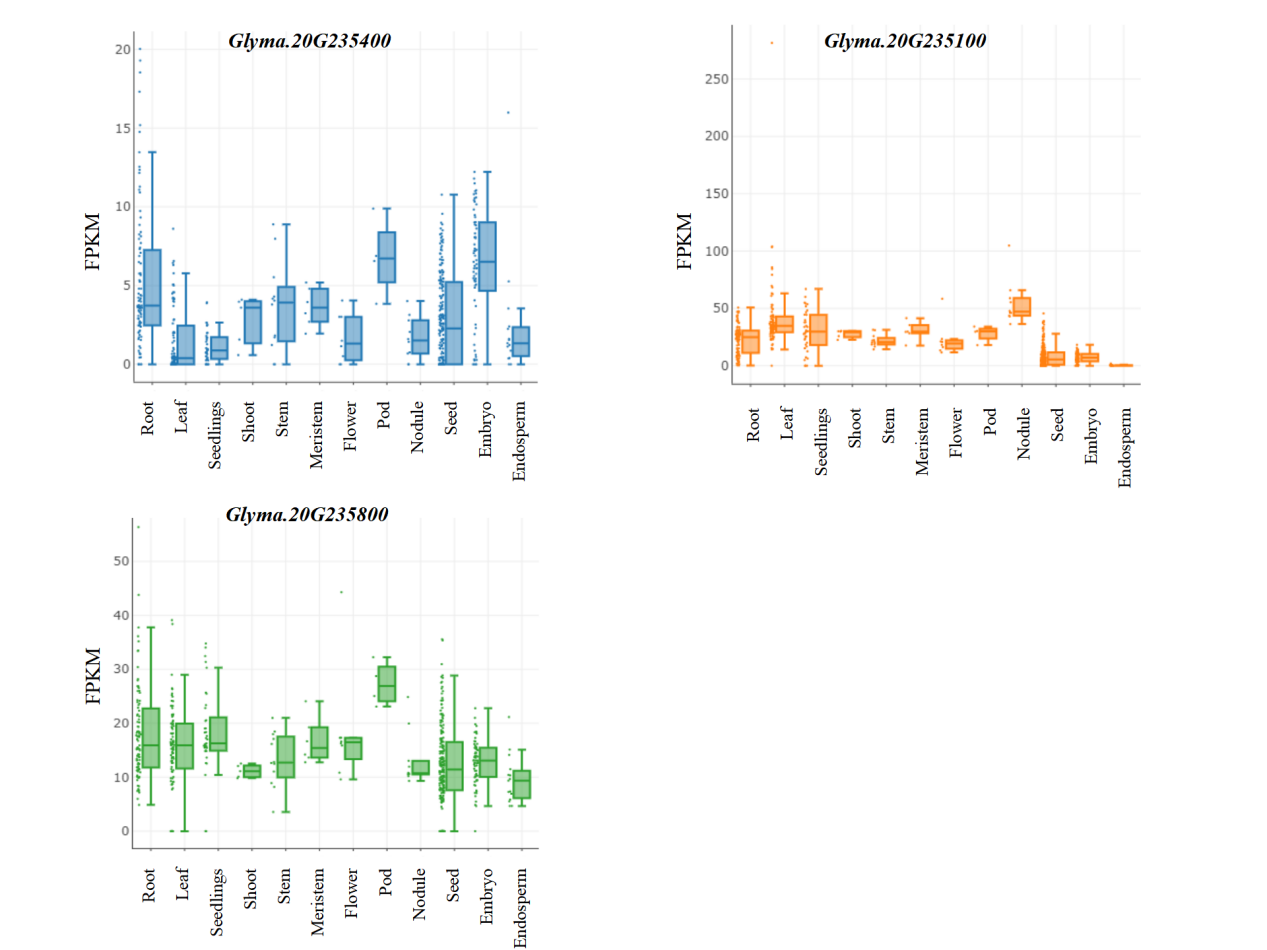


**Figure S11 C**andidate genes expression levels in each tissues.


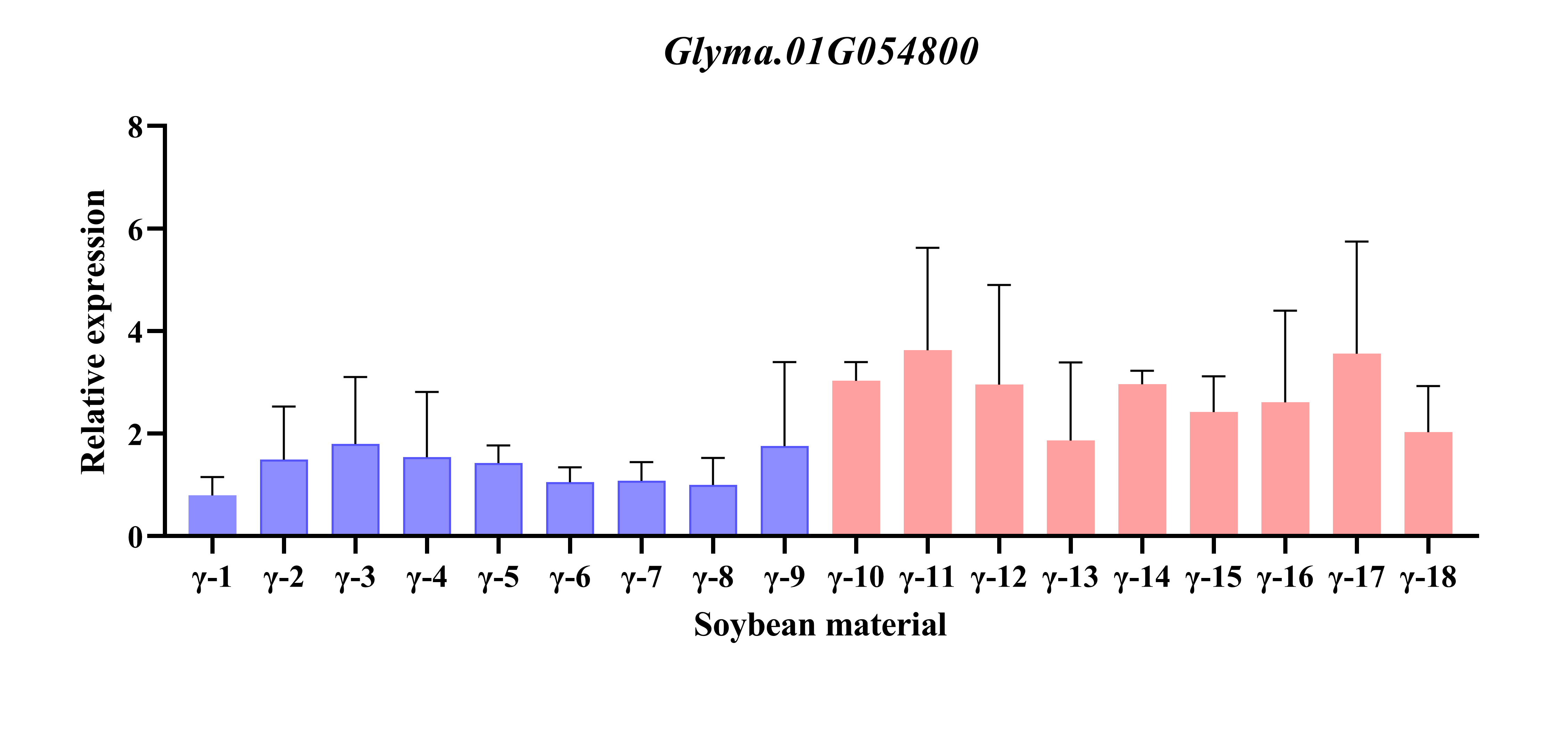

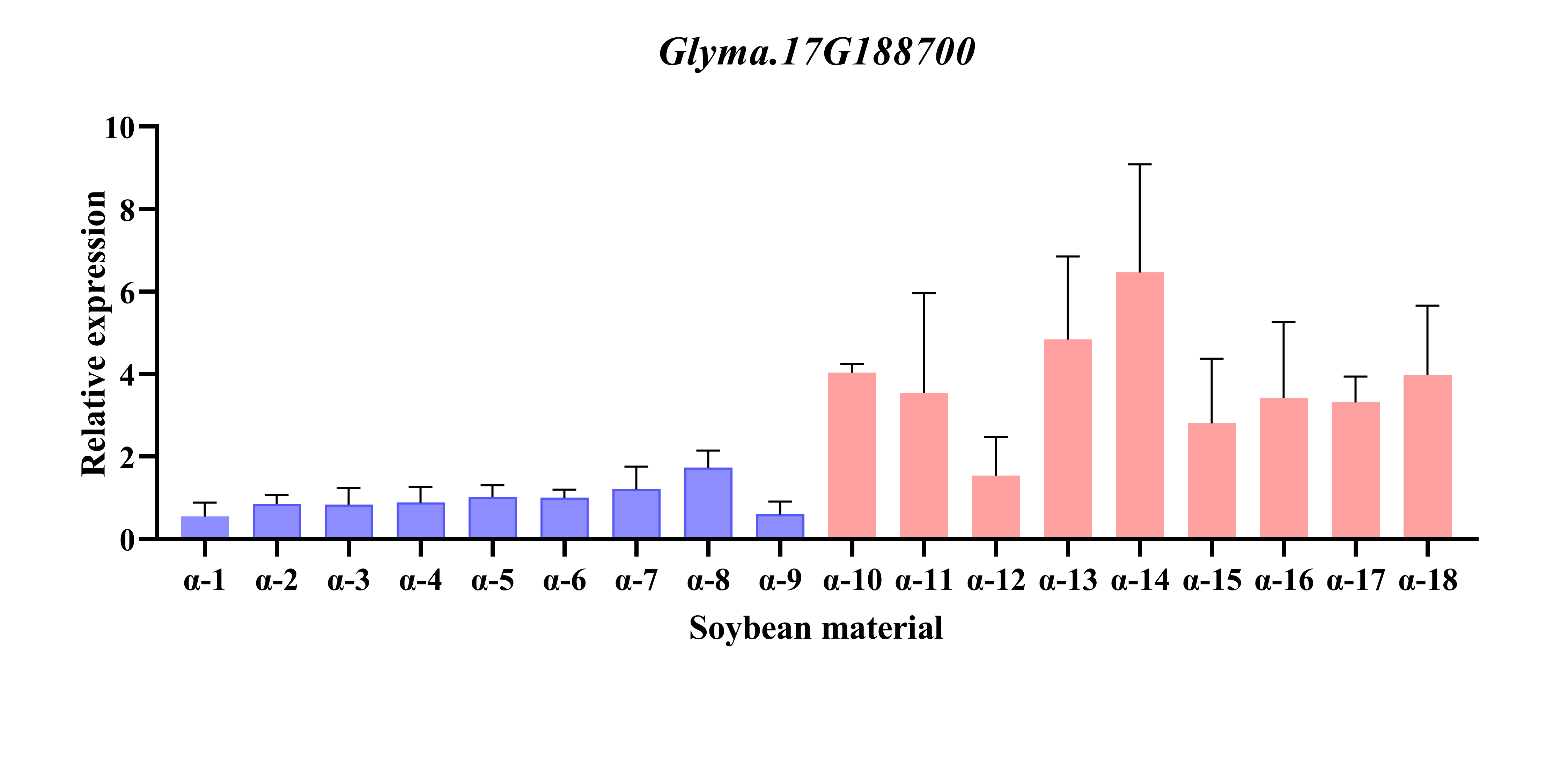

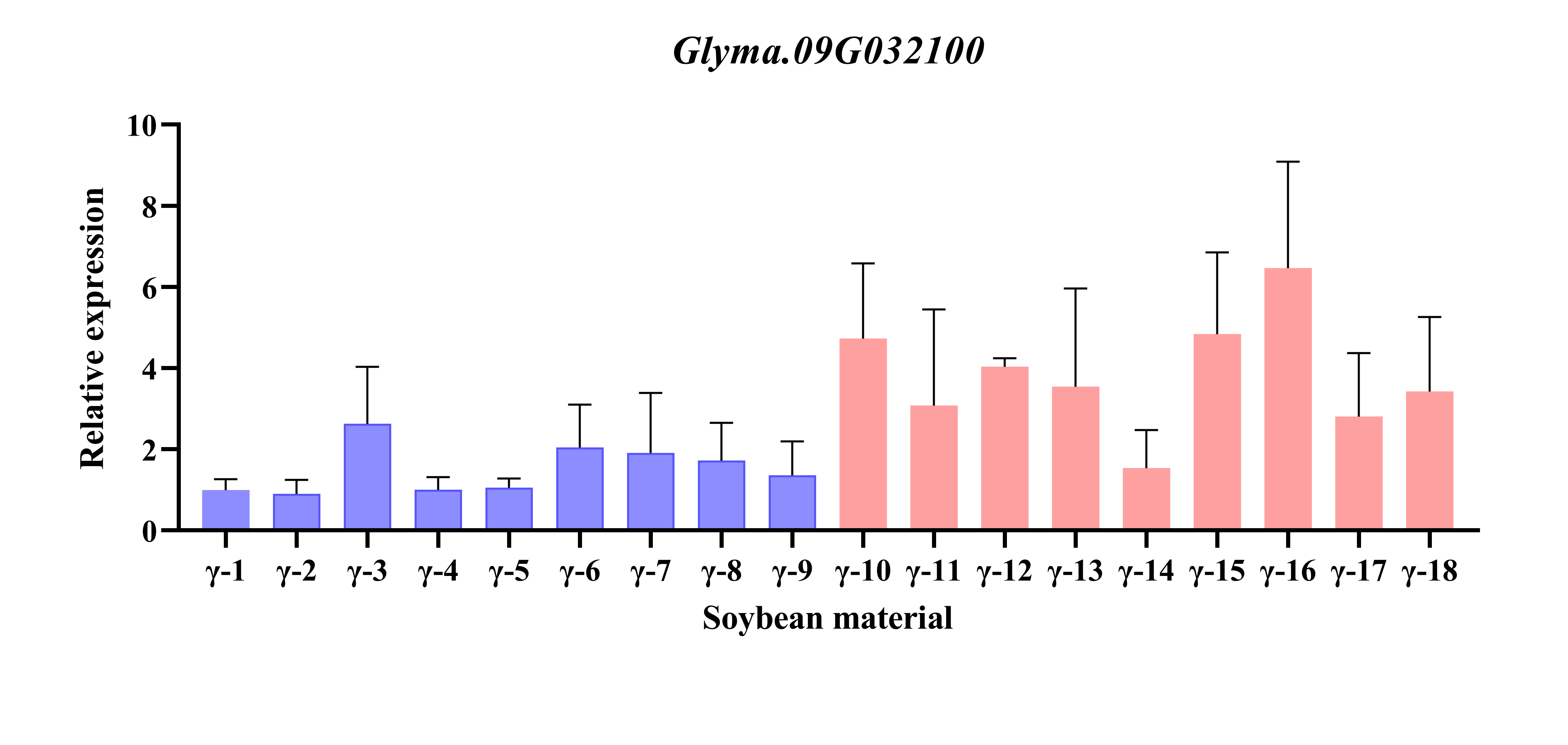

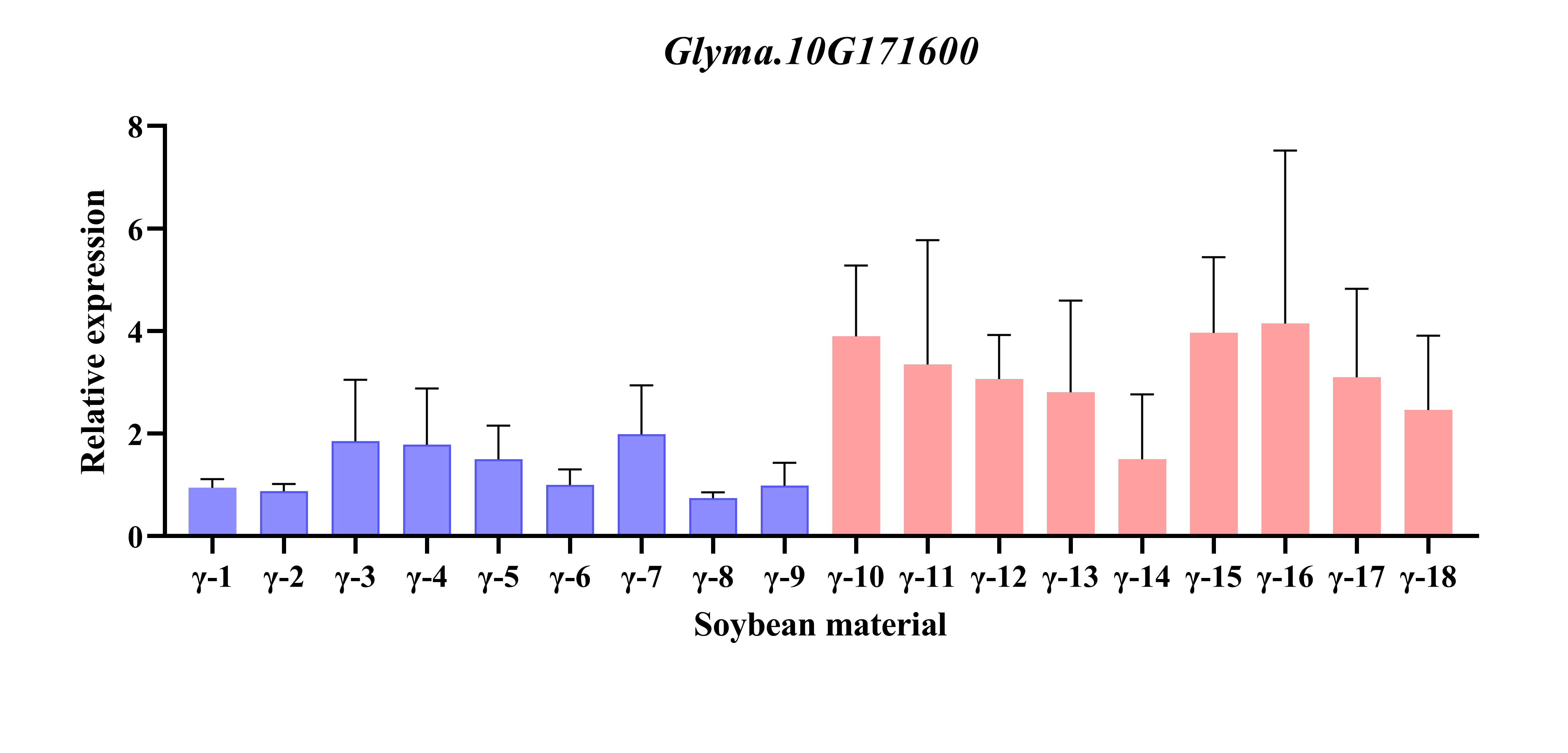

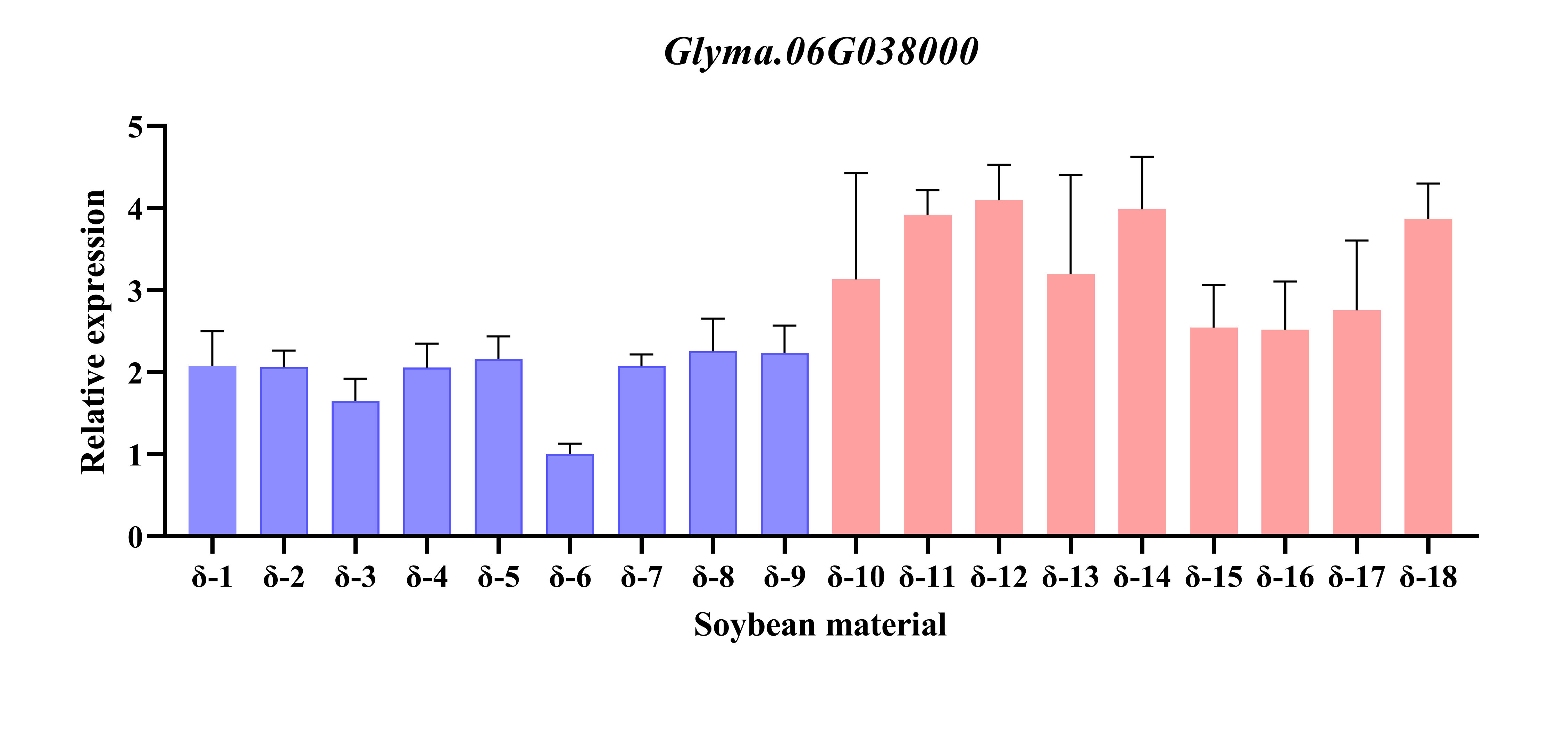

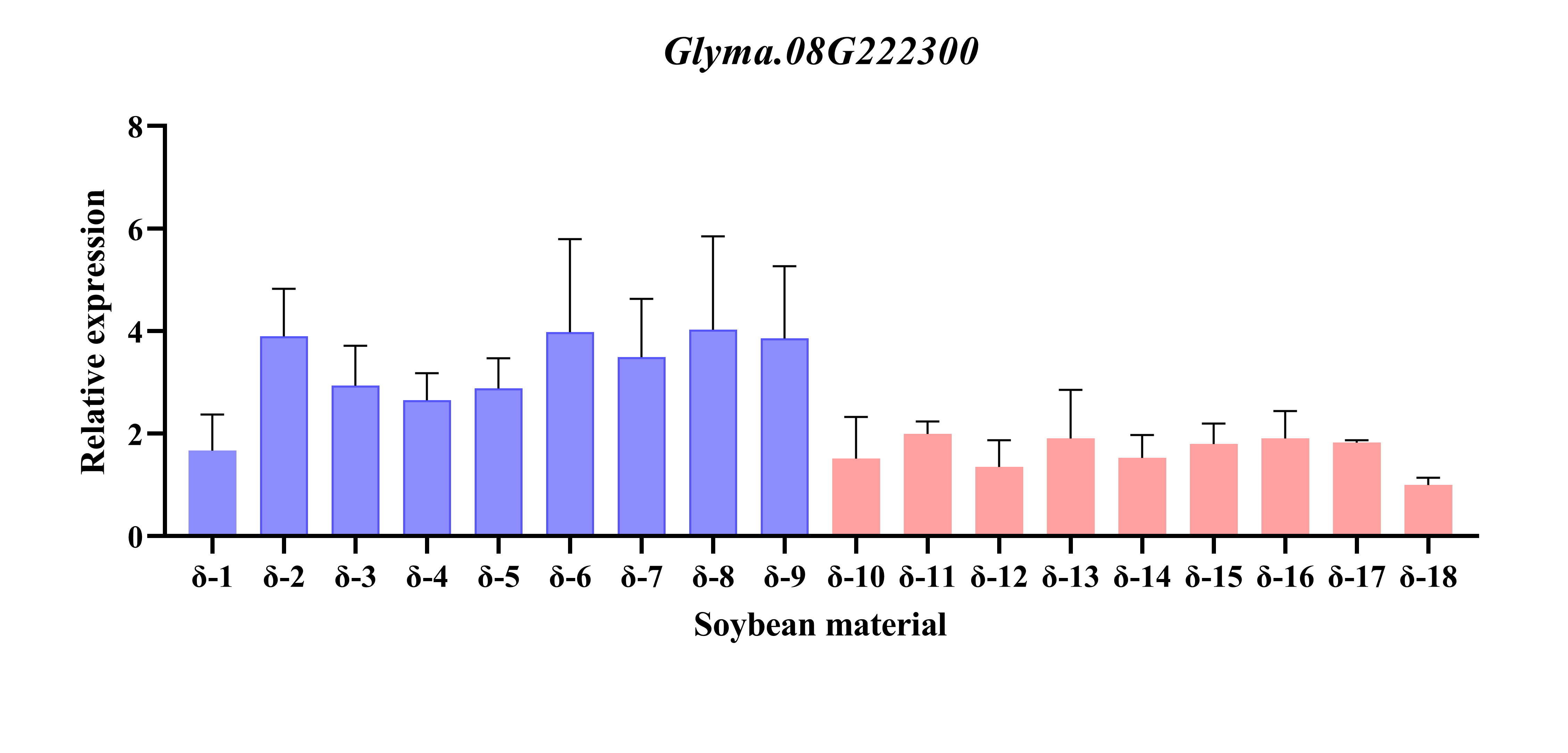

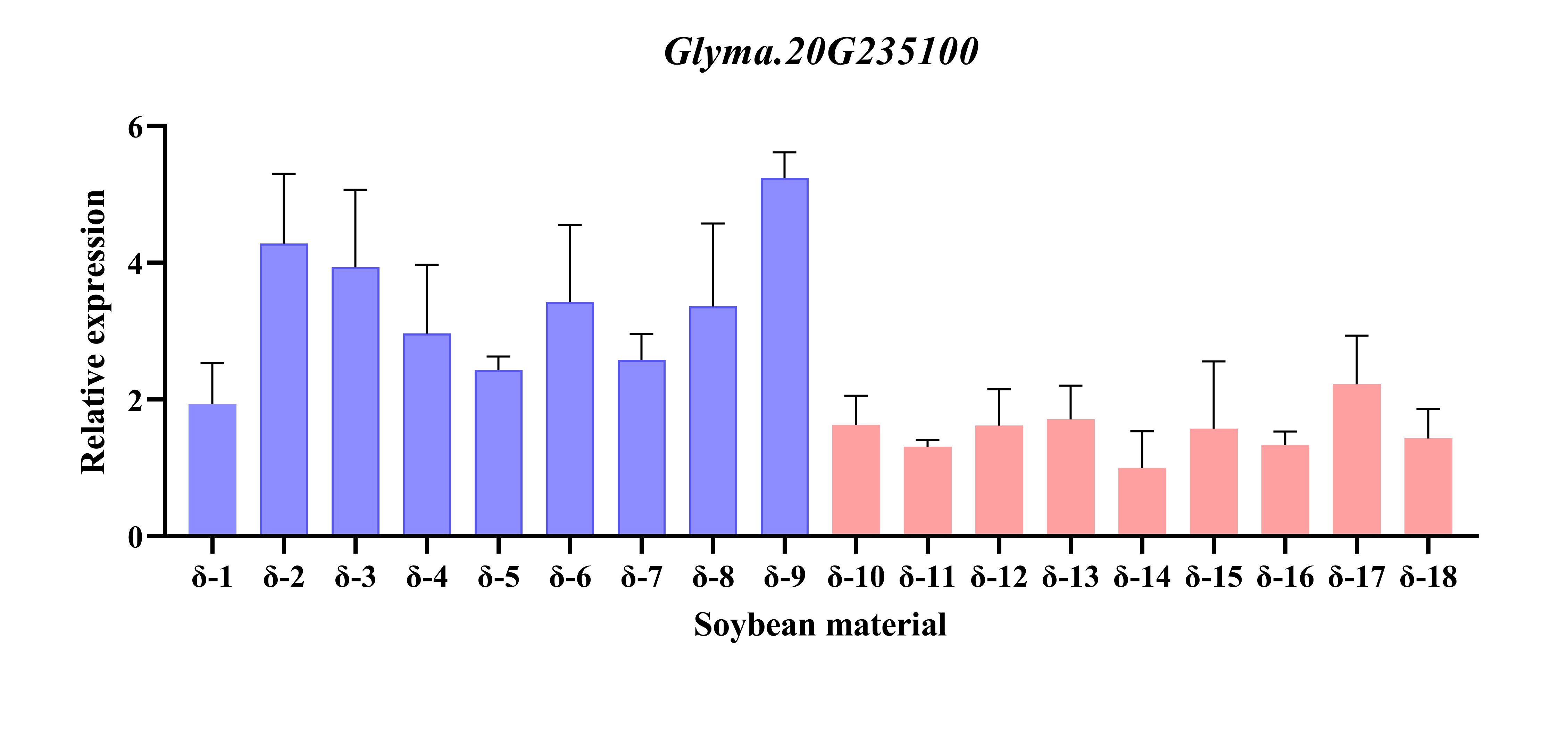

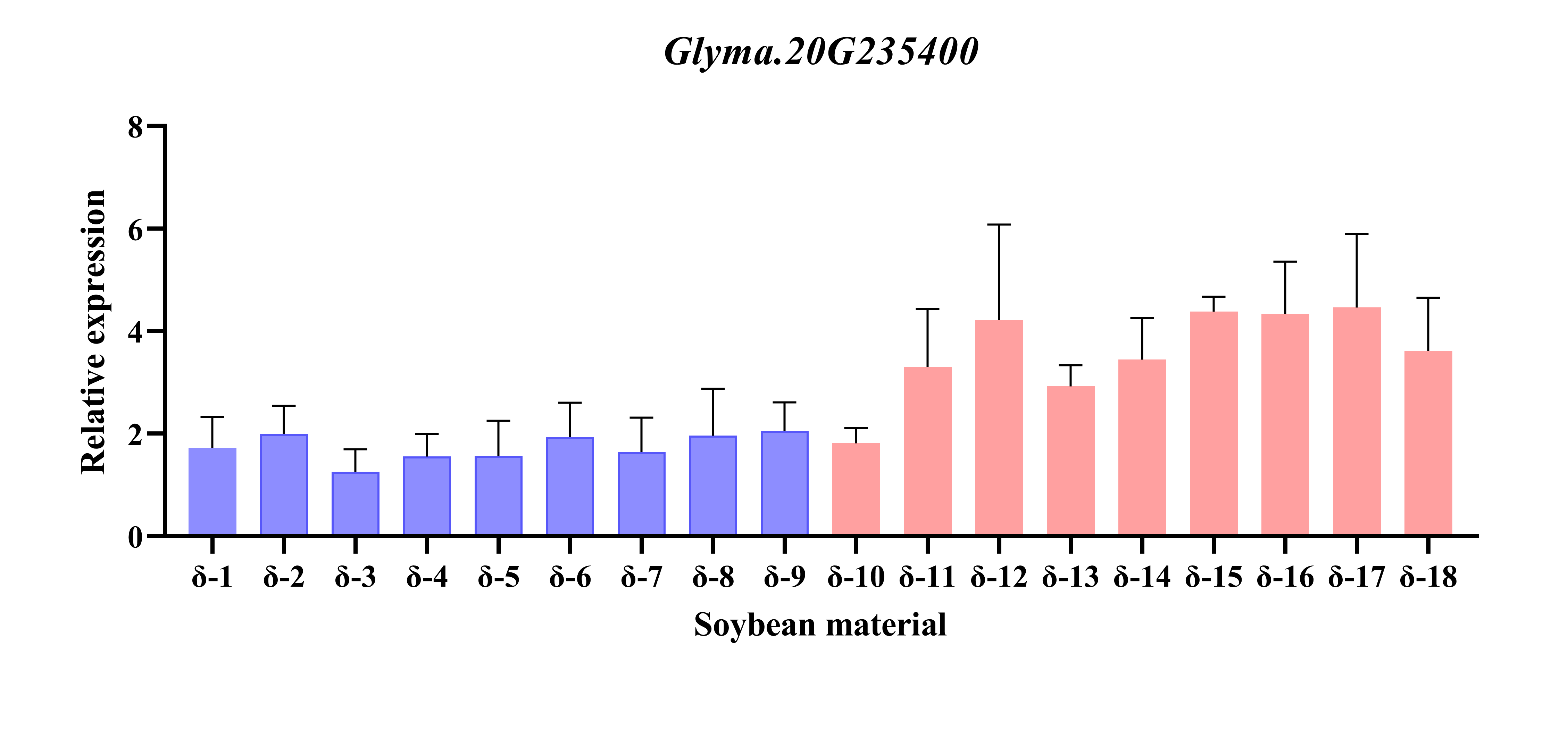

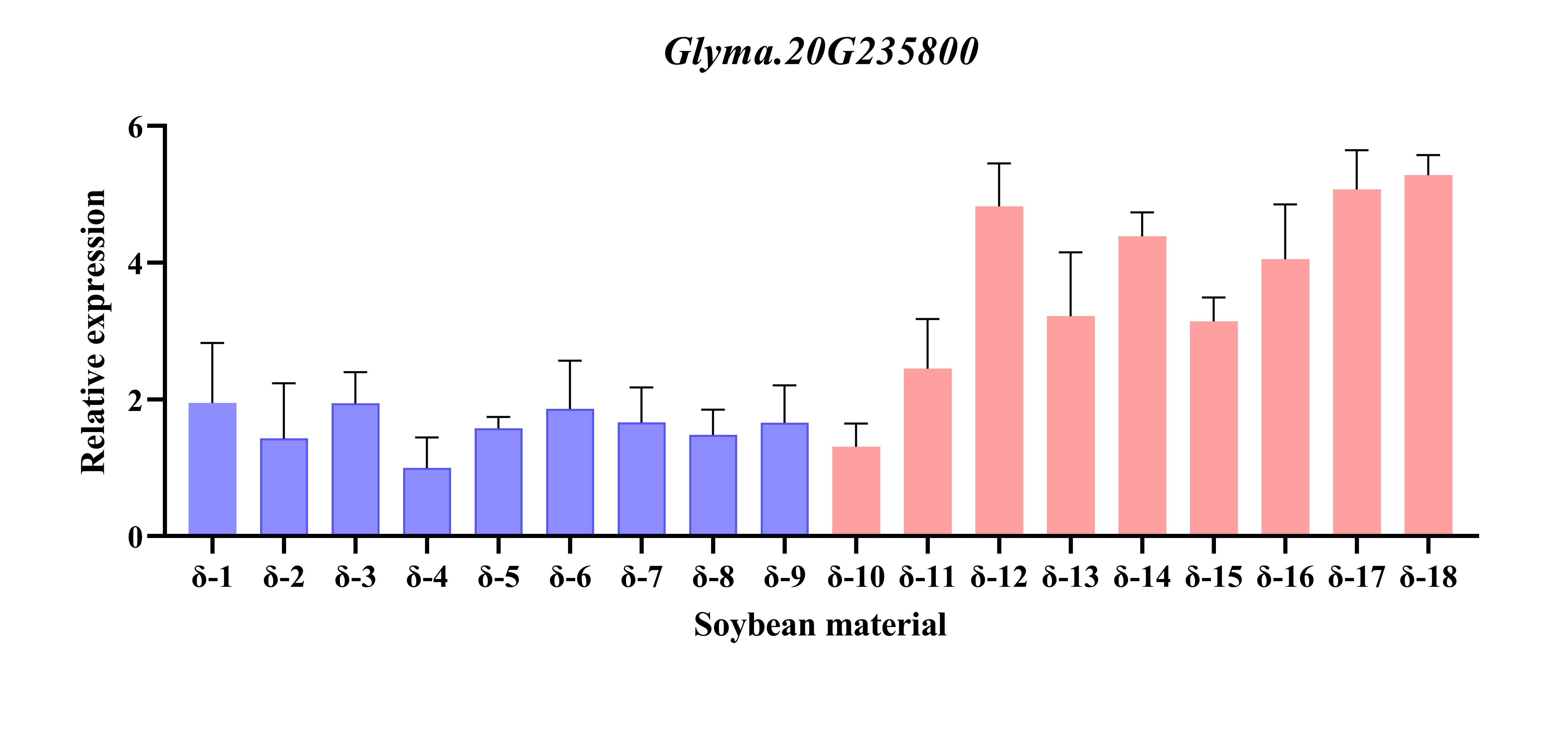

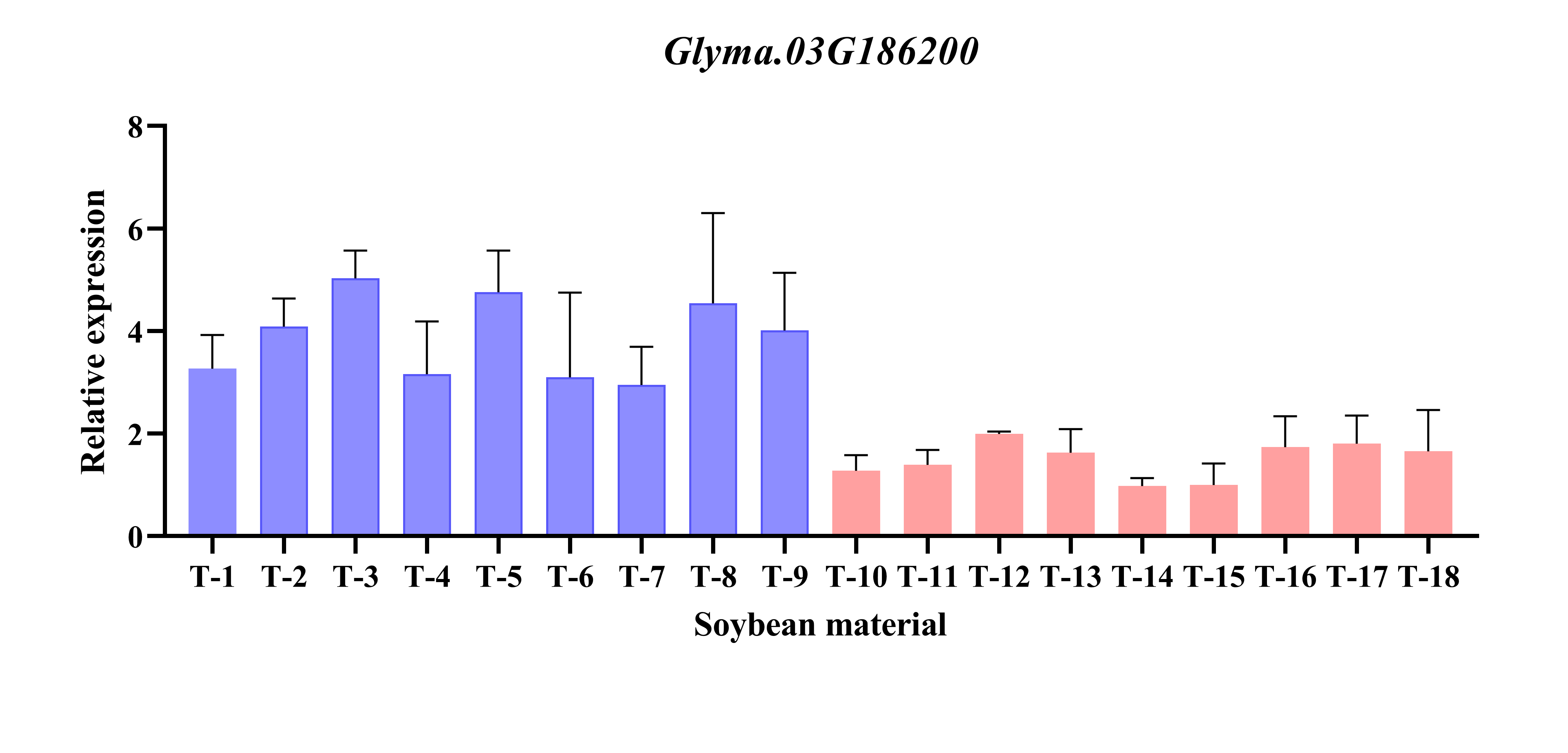

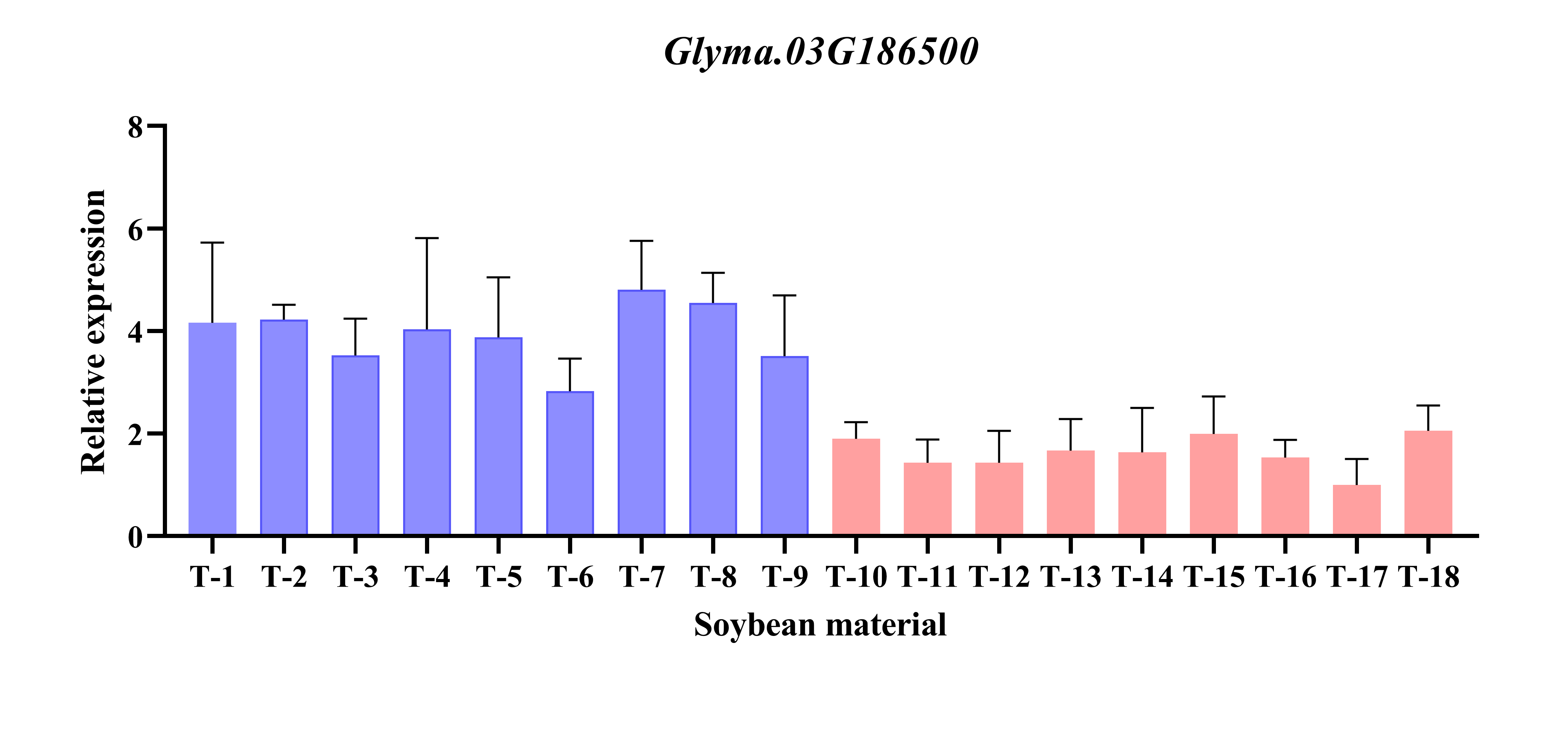

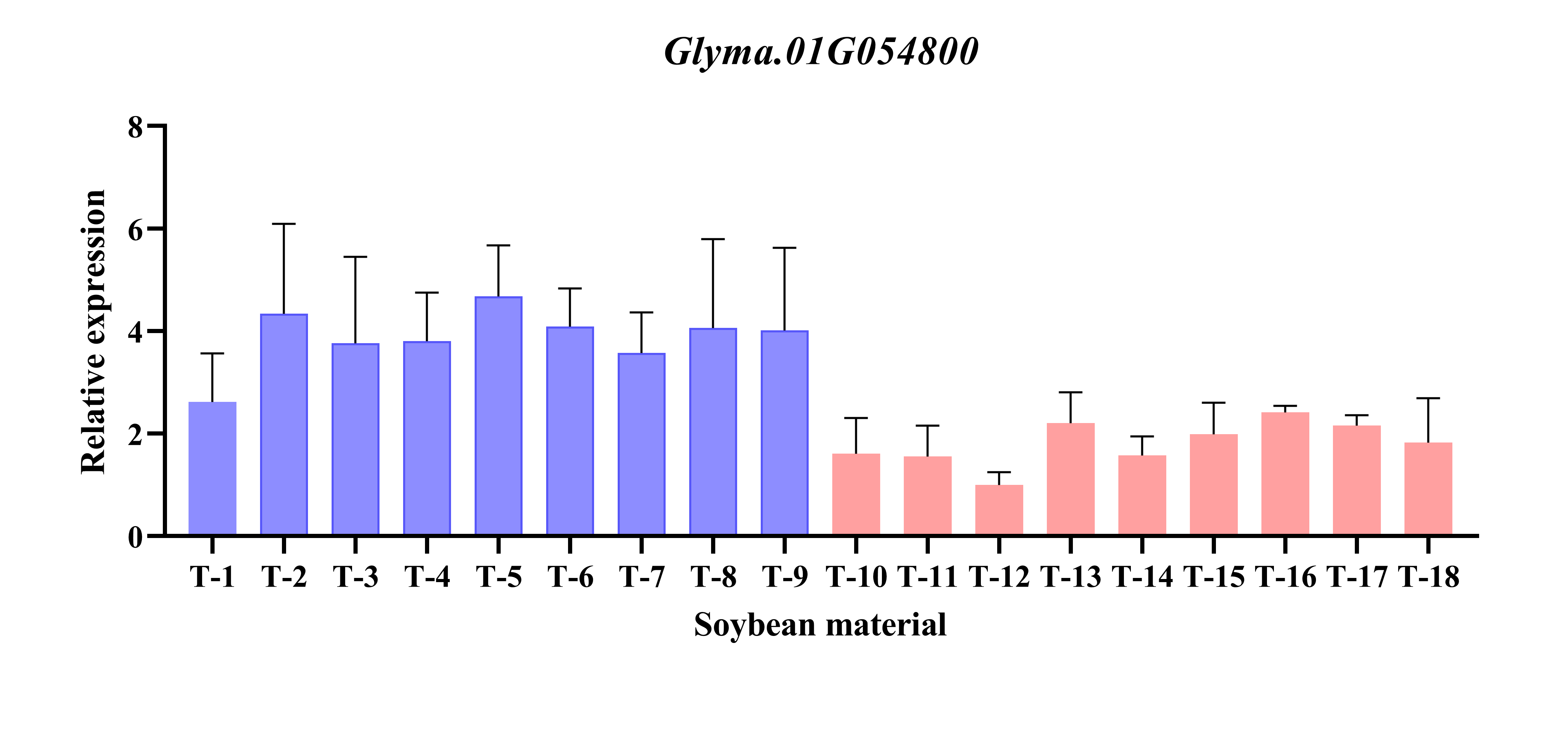

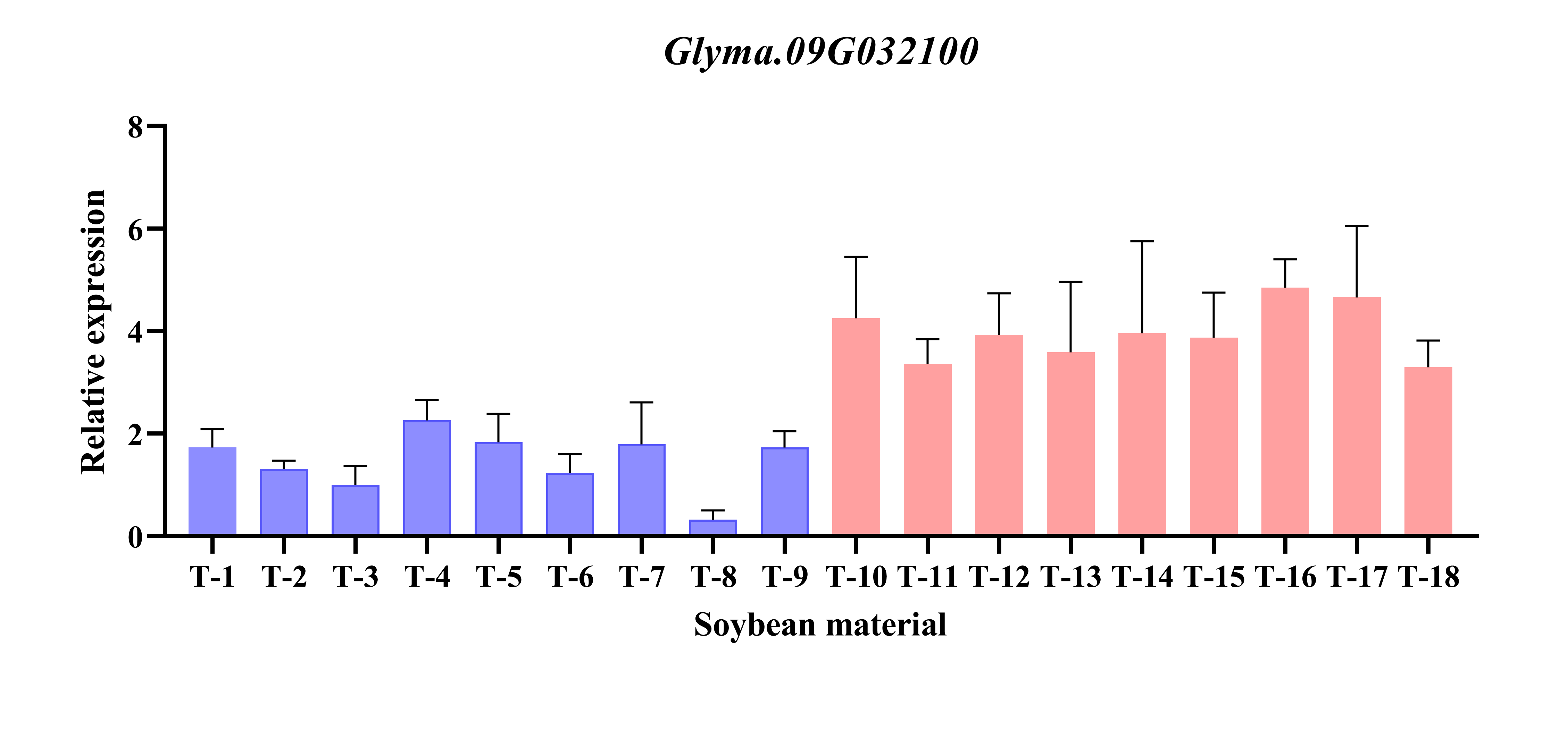

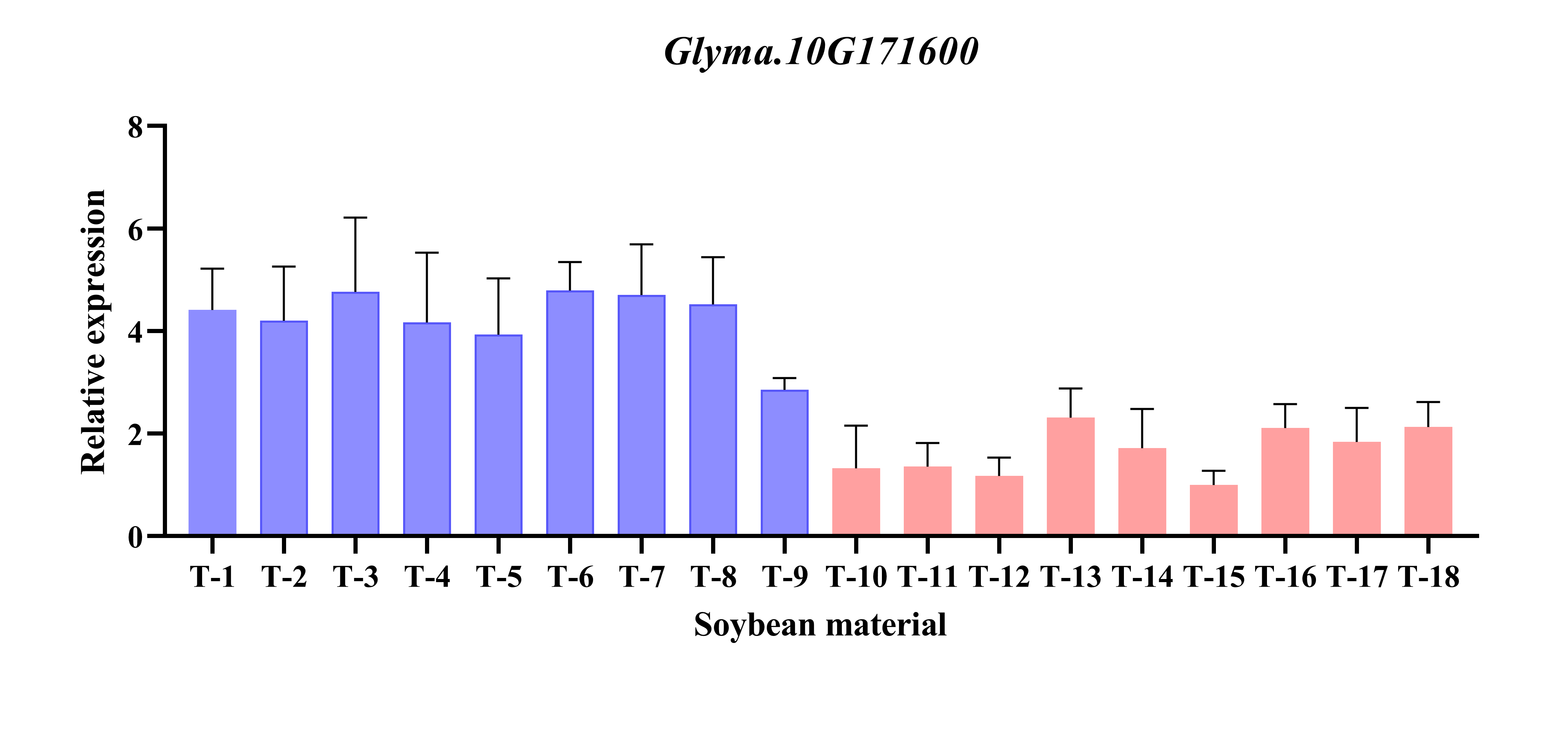


**Figure S12** Candidate genes expression analysis by qRT-PCR.

| **Table S1** The information of 175 soybean accessions. | | | |
| --- | --- | --- | --- |
| Name | Country | Latitude (°N) | Longitude (°W) |
| Big black | China | 36.23 | 118.31 |
| Cateye | China | 37.34 | 100.30 |
| Cdou1 | China | 37.34 | 84.79 |
| Small yellow | China | 37.39 | 112.24 |
| JD17 | China | 39.19 | 116.29 |
| Stone | China | 39.19 | 116.29 |
| Datenhei | China | 39.19 | 116.29 |
| JD12 | China | 39.19 | 116.29 |
| Jin13 | China | 39.19 | 116.29 |
| J06B7 | China | 39.19 | 116.29 |
| HD5 | China | 39.19 | 116.29 |
| HX446 | China | 39.19 | 116.29 |
| ZZ00-683 | China | 40.15 | 116.28 |
| ZD27 | China | 40.15 | 116.28 |
| Z10 | China | 40.15 | 116.28 |
| ZD35 | China | 40.15 | 116.28 |
| Z20 | China | 40.15 | 116.28 |
| Early18 | China | 40.15 | 116.28 |
| KX3 | China | 40.15 | 116.28 |
| Z30 | China | 40.15 | 116.28 |
| Z03-5179 | China | 40.15 | 116.28 |
| Zhonghuang35 | China | 40.15 | 116.28 |
| Z95-5388 | China | 40.15 | 116.28 |
| Z03-5334 | China | 40.15 | 116.28 |
| ZZJ4133 | China | 40.15 | 116.28 |
| Z03-5373 | China | 40.15 | 116.28 |
| JZ4-1 | China | 40.85 | 122.00 |
| L98072 | China | 40.85 | 122.00 |
| TD54 | China | 40.85 | 122.00 |
| LN2 | China | 40.85 | 122.00 |
| Tejia | China | 40.85 | 122.00 |
| LX1 | China | 40.85 | 122.00 |
| Hangbaodou | China | 40.85 | 122.00 |
| Baiqidou | China | 40.85 | 122.00 |
| TD51 | China | 40.85 | 122.00 |
| Tiefeng31 | China | 40.85 | 122.00 |
| TF8 | China | 40.85 | 122.00 |
| TD50 | China | 40.85 | 122.00 |
| TD52 | China | 40.85 | 122.00 |
| Sixty days | China | 40.85 | 122.00 |
| TD58 | China | 40.85 | 122.00 |
| LD3 | China | 40.85 | 122.00 |
| LD23 | China | 40.85 | 122.00 |
| JD33 | China | 40.85 | 122.00 |
| Nova | Italy | 41.54 | 12.30 |
| L-9 | USA | 41.83 | 92.91 |
| L-57 | USA | 41.83 | 92.91 |
| L-57 | USA | 41.83 | 92.91 |
| L-59Peking | USA | 41.83 | 92.91 |
| L-21 | USA | 41.83 | 92.91 |
| L-10 | USA | 41.83 | 92.91 |
| charleston | USA | 41.83 | 92.91 |
| L-9 | USA | 41.83 | 92.91 |
| Williams82 | USA | 41.83 | 92.91 |
| Fendiyellow | China | 43.35 | 126.29 |
| TN13 | China | 43.35 | 126.29 |
| JN21 | China | 43.35 | 126.29 |
| JL30 | China | 43.35 | 126.29 |
| J89 | China | 43.35 | 126.29 |
| JY94 | China | 43.35 | 126.29 |
| Bmoshi | China | 43.35 | 126.29 |
| Gonye04-141 | China | 43.35 | 126.29 |
| J100 | China | 43.35 | 126.29 |
| 4tejia | China | 43.35 | 126.29 |
| Douludou | China | 43.35 | 126.29 |
| HYoutai | China | 43.35 | 126.29 |
| JN20 | China | 43.35 | 126.29 |
| Cse | China | 43.35 | 126.29 |
| JL47 | China | 43.35 | 126.29 |
| Jshanmoshi | China | 43.35 | 126.29 |
| Jcha | China | 43.35 | 126.29 |
| V111-4 | China | 43.35 | 126.29 |
| Heimodou | China | 43.35 | 126.29 |
| DY2004-5 | China | 43.35 | 126.29 |
| Chamoshidou | China | 43.35 | 126.29 |
| L-79 | Canada | 43.40 | 79.25 |
| DN L-13 | Canada | 43.40 | 79.25 |
| DN50 | Canada | 43.40 | 79.25 |
| L-28 | Canada | 43.40 | 79.25 |
| Aika166 | Rumania | 44.23 | 26.10 |
| MD14 | China | 44.49 | 111.70 |
| MD9 | China | 44.49 | 111.70 |
| MD21 | China | 44.49 | 111.70 |
| HJ423 | China | 44.49 | 111.70 |
| HJ04-528 | China | 44.49 | 111.70 |
| Domaka Tolisa-A | Yugoslavia | 46.30 | 14.30 |
| HH38 | China | 48.29 | 128.08 |
| BF9 | China | 48.29 | 128.08 |
| HF35 | China | 48.29 | 128.08 |
| Ken15 | China | 48.29 | 128.08 |
| NF11 | China | 48.29 | 128.08 |
| Heilongjiang41 | China | 48.29 | 128.08 |
| SN10 | China | 48.29 | 128.08 |
| HH18 | China | 48.29 | 128.08 |
| B4834 | China | 48.29 | 128.08 |
| DN48 | China | 48.29 | 128.08 |
| HN37 | China | 48.29 | 128.08 |
| NF15 | China | 48.29 | 128.08 |
| Fenshou6 | China | 48.29 | 128.08 |
| Jishanpu | China | 48.29 | 128.08 |
| HF47 | China | 48.29 | 128.08 |
| HF11 | China | 48.29 | 128.08 |
| HN51 | China | 48.29 | 128.08 |
| Suinong4 | China | 48.29 | 128.08 |
| HF45 | China | 48.29 | 128.08 |
| S02-339 | China | 48.29 | 128.08 |
| K29 | China | 48.29 | 128.08 |
| DN47 | China | 48.29 | 128.08 |
| S28 | China | 48.29 | 128.08 |
| DN46 | China | 48.29 | 128.08 |
| Yanbojin | China | 48.29 | 128.08 |
| MF1 | China | 48.29 | 128.08 |
| Ken18 | China | 48.29 | 128.08 |
| DN49 | China | 48.29 | 128.08 |
| DN43 | China | 48.29 | 128.08 |
| KJ23 | China | 48.29 | 128.08 |
| LongX1 | China | 48.29 | 128.08 |
| S29 | China | 48.29 | 128.08 |
| DN594 | China | 48.29 | 128.08 |
| S03-3952 | China | 48.29 | 128.08 |
| Ken14 | China | 48.29 | 128.08 |
| S04-5804 | China | 48.29 | 128.08 |
| SN1 | China | 48.29 | 128.08 |
| DN1068 | China | 48.29 | 128.08 |
| K04-8579 | China | 48.29 | 128.08 |
| DN44 | China | 48.29 | 128.08 |
| HF25 | China | 48.29 | 128.08 |
| SN14 | China | 48.29 | 128.08 |
| HF52 | China | 48.29 | 128.08 |
| S30 | China | 48.29 | 128.08 |
| SN25 | China | 48.29 | 128.08 |
| HF50 | China | 48.29 | 128.08 |
| Small Moshi | China | 48.29 | 128.08 |
| H05-31 | China | 48.29 | 128.08 |
| DN56 | China | 48.29 | 128.08 |
| Xian2 | China | 48.29 | 128.08 |
| Qian black | China | 48.29 | 128.08 |
| Xian3 | China | 48.29 | 128.08 |
| Huajian2 | China | 48.29 | 128.08 |
| LP03-11 | China | 48.29 | 128.08 |
| Ken22 | China | 48.29 | 128.08 |
| DN93046 | China | 48.29 | 128.08 |
| S04-6018 | China | 48.29 | 128.08 |
| S05-7304 | China | 48.29 | 128.08 |
| HN55 | China | 48.29 | 128.08 |
| DN42 | China | 48.29 | 128.08 |
| B1361 | China | 48.29 | 128.08 |
| H04-1824 | China | 48.29 | 128.08 |
| Harvest10 | China | 48.29 | 128.08 |
| 4yellow | China | 48.29 | 128.08 |
| Yapiche | China | 48.29 | 128.08 |
| BD14 | China | 48.29 | 128.08 |
| B1484 | China | 48.29 | 128.08 |
| B1873 | China | 48.29 | 128.08 |
| HN48 | China | 48.29 | 128.08 |
| S03-3046 | China | 48.29 | 128.08 |
| Hujiao03-286 | China | 48.29 | 128.08 |
| HuaJiang4403 | China | 48.29 | 128.08 |
| HF29 | China | 48.29 | 128.08 |
| H05-991 | China | 48.29 | 128.08 |
| HN44 | China | 48.29 | 128.08 |
| K30 | China | 48.29 | 128.08 |
| Lonqan | China | 48.29 | 128.08 |
| FZmoshidou | China | 48.29 | 128.08 |
| Heinong33 | China | 48.29 | 128.08 |
| HF55 | China | 48.29 | 128.08 |
| HH48 | China | 48.29 | 128.08 |
| DN07-909 | China | 48.29 | 128.08 |
| HH45 | China | 48.29 | 128.08 |
| BD16 | China | 48.29 | 128.08 |
| HF37 | China | 48.29 | 128.08 |
| SN8 | China | 48.29 | 128.08 |
| Heersong2 | Ukraine | 50.28 | 30.29 |
| Boige du | Germany | 52.31 | 13.20 |
| Dunajika | Russia | 61.50 | 98.00 |

| **Table S2** Primers used for quantitative real-time PCR. | | |
| --- | --- | --- |
| Gene ID | Forward primer (5'-3') | Reverse primer (5'-3') |
| *Glyma.01G054800* | TTGTGCTAAAACATCTGTGTCG | CAAGCACCTCAACATCTTTTGA |
| *Glyma.09G032100* | CATCATCGGATTCGTTTGGTAC | TCAGTTGATCATGTGCTTGTTG |
| *Glyma.10G277800* | TATTGGAGATTTGGAGCACGAA | CTCGTTGTCGTTTTTCTCAGTT |
| *Glyma.08G222300* | CTCCTCAATTCTAATCTCGCCT | AGTTTGTACTTTCAAAGCCGAC |
| *Glyma.20G235100* | GAAGATGCAGTAGTTAAGCAGC | GATTGTGAGTCTGTGTTGGAAC |
| *Glyma.20G235400* | GGAAAAATTGTGTCTGGATGCT | ACAACTAATGACACTCTAGCGT |
| *Glyma.20G235800* | TACAGCCTTGCTTTTTCTCCTA | CTTGAGGTTGAAGACATGAACG |
| *Glyma.03G186200* | AATAAGTTGTTGGTGTGTGTGG | CTAGGGAAGGCTAAGCTGTAAG |
| *Glyma.03G186500* | GCTCTTCGTTACAACAAGACTG | AACAAGCTTTTTCCCAGAACTC |
| *Glyma.17G188700* | CTTATGTCAGATGTTTGTGAGG | AATGTAGCCTACAAGCTTTATG |
| *GmActin4* | GTTTCAAGCTCTTGCTCGTAATCA | GTGTCAGCCATACTGTCCCCATTT |

| **Table S3** Peak SNP associated with the Toc content identified by GWAS using the GLM model. | | | | | | | |
| --- | --- | --- | --- | --- | --- | --- | --- |
| SNP | Traits | Environment | Chr. | Position | -log10（P） | MAF | Allele |
| rs9337368 | δ-Toc content | Harbin | 2 | 9337368 | 4.49 | 0.06 | A/T |
| rs19659048 | δ-Toc content | Harbin | 3 | 19659048 | 4.39 | 0.06 | A/G |
| rs19699991 | δ-Toc content | Harbin | 3 | 19699991 | 4.02 | 0.04 | C/T |
| rs19718856 | δ-Toc content | Harbin | 3 | 19718856 | 4.65 | 0.04 | T/C |
| rs19737458 | δ-Toc content | Harbin | 3 | 19737458 | 5.31 | 0.05 | T/A |
| rs20683456 | δ-Toc content | Harbin | 3 | 20683456 | 4.32 | 0.05 | C/A |
| rs20762556 | δ-Toc content | Harbin | 3 | 20762556 | 4.65 | 0.04 | C/A |
| rs20959996 | δ-Toc content | Harbin | 3 | 20959996 | 5.38 | 0.05 | A/G |
| rs21181591 | δ-Toc content | Harbin | 3 | 21181591 | 4.65 | 0.04 | T/C |
| rs21525954 | δ-Toc content | Harbin | 3 | 21525954 | 4.65 | 0.04 | C/A |
| rs21690828 | δ-Toc content | Harbin | 3 | 21690828 | 4.46 | 0.04 | G/A |
| rs22050090 | δ-Toc content | Harbin | 3 | 22050090 | 4.65 | 0.04 | C/A |
| rs22225963 | δ-Toc content | Harbin | 3 | 22225963 | 4.32 | 0.05 | T/C |
| rs22701979 | δ-Toc content | Harbin | 3 | 22701979 | 4.51 | 0.04 | G/A |
| rs22836247 | δ-Toc content | Harbin | 3 | 22836247 | 4.20 | 0.03 | G/T |
| rs22991419 | δ-Toc content | Harbin | 3 | 22991419 | 4.48 | 0.04 | G/A |
| rs23032537 | δ-Toc content | Harbin | 3 | 23032537 | 4.65 | 0.04 | A/G |
| rs23126123 | δ-Toc content | Harbin | 3 | 23126123 | 4.65 | 0.04 | C/T |
| rs23126538 | δ-Toc content | Harbin | 3 | 23126538 | 4.65 | 0.04 | G/T |
| rs23391671 | δ-Toc content | Harbin | 3 | 23391671 | 4.18 | 0.04 | T/C |
| rs27765335 | δ-Toc content | Harbin | 3 | 27765335 | 4.45 | 0.08 | G/T |
| rs27793445 | δ-Toc content | Harbin | 3 | 27793445 | 4.18 | 0.08 | G/A |
| rs30721039 | δ-Toc content | Harbin | 3 | 30721039 | 4.13 | 0.05 | A/G |
| rs39077086 | α-Toc content | Harbin | 3 | 39077086 | 4.38 | 0.43 | T/C |
| rs39895210 | Total-Toc content | Liaoning | 3 | 39895210 | 4.52 | 0.27 | G/A |
| rs2960931 | δ-Toc content | Liaoning | 6 | 2960931 | 4.47 | 0.19 | G/A |
| rs1834346 | α-Toc content | Harbin | 8 | 1834346 | 4.06 | 0.03 | A/T |
| rs19310064 | α-Toc content | Harbin | 8 | 19310064 | 9.43 | 0.01 | A/C |
| rs31044180 | α-Toc content | Jilin | 9 | 31044180 | 4.43 | 0.08 | G/T |
|  | γ-Toc content | Jilin |  |  | 5.15 |  |  |
|  | Total-Toc content | Jilin |  |  | 4.22 |  |  |
| rs7284532 | α-Toc content | Harbin | 10 | 7284532 | 4.19 | 0.05 | G/A |
| rs7543892 | δ-Toc content | Jilin | 10 | 7543892 | 4.25 | 0.06 | T/G |
| rs39590600 | δ-Toc content | Harbin | 10 | 39590600 | 4.78 | 0.04 | G/A |
| rs49928375 | α-Toc content | Harbin | 10 | 49928375 | 5.74 | 0.06 | G/T |
| rs17125409 | α-Toc content | Harbin | 12 | 17125409 | 4.56 | 0.07 | C/A |
| rs38228354 | δ-Toc content | Harbin | 12 | 38228354 | 4.07 | 0.07 | A/G |
| rs330000 | δ-Toc content | Harbin | 13 | 330000 | 4.76 | 0.17 | G/A |
|  | δ-Toc content | Liaoning |  |  | 4.96 |  |  |
| rs10613826 | δ-Toc content | Harbin | 13 | 10613826 | 4.19 | 0.13 | C/T |
| rs10747461 | δ-Toc content | Harbin | 13 | 10747461 | 4.44 | 0.11 | C/T |
| rs11184836 | δ-Toc content | Harbin | 13 | 11184836 | 4.80 | 0.11 | G/A |
| rs11500692 | δ-Toc content | Harbin | 13 | 11500692 | 4.38 | 0.14 | C/T |
| rs12187473 | δ-Toc content | Harbin | 13 | 12187473 | 4.36 | 0.11 | C/T |
| rs8803536 | α-Toc content | Harbin | 14 | 8803536 | 4.54 | 0.07 | T/A |
| rs9782629 | γ-Toc content | Harbin | 14 | 9782629 | 4.89 | 0.07 | G/T |
| rs2417467 | α-Toc content | Harbin | 16 | 2417467 | 4.18 | 0.43 | A/C |
| rs19530677 | γ-Toc content | Harbin | 16 | 19530677 | 6.20 | 0.05 | T/A |
| rs31159905 | δ-Toc content | Harbin | 16 | 31159905 | 4.27 | 0.35 | T/C |
| rs2780598 | Total-Toc content | Harbin | 17 | 2780598 | 5.49 | 0.02 | C/G |
| rs14593163 | δ-Toc content | Harbin | 17 | 14593163 | 4.73 | 0.20 | T/G |
|  | Total-Toc content | Harbin |  |  | 6.12 |  |  |
| rs24979561 | α-Toc content | Harbin | 17 | 24979561 | 6.53 | 0.07 | G/A |
| rs588498 | α-Toc content | Liaoning | 18 | 588498 | 4.26 | 0.43 | G/A |
| rs19903533 | δ-Toc content | Harbin | 18 | 19903533 | 4.04 | 0.03 | G/T |
| rs19962490 | δ-Toc content | Harbin | 18 | 19962490 | 4.84 | 0.02 | T/C |
|  | Total-Toc content | Harbin |  |  | 4.37 |  |  |
| rs49413625 | δ-Toc content | Harbin | 18 | 49413625 | 4.27 | 0.45 | T/C |
| rs53062844 | α-Toc content | Liaoning | 18 | 53062844 | 5.73 | 0.08 | G/T |
| rs7528700 | δ-Toc content | Harbin | 19 | 7528700 | 4.11 | 0.39 | A/G |
| rs8705363 | δ-Toc content | Harbin | 19 | 8705363 | 5.56 | 0.16 | G/A |
| rs8705388 | δ-Toc content | Harbin | 19 | 8705388 | 5.12 | 0.15 | G/A |
| rs8720055 | δ-Toc content | Harbin | 19 | 8720055 | 6.06 | 0.15 | T/A |
| rs8720462 | δ-Toc content | Harbin | 19 | 8720462 | 6.82 | 0.17 | G/A |
|  |  | Liaoning |  |  | 4.48 |  |  |
| rs8782541 | δ-Toc content | Harbin | 19 | 8782541 | 5.10 | 0.15 | G/A |
| rs8917233 | δ-Toc content | Harbin | 19 | 8917233 | 4.32 | 0.16 | C/A |
| rs8988072 | δ-Toc content | Harbin | 19 | 8988072 | 5.44 | 0.16 | C/T |
| rs8988491 | δ-Toc content | Harbin | 19 | 8988491 | 5.23 | 0.17 | C/T |
| rs9156253 | δ-Toc content | Harbin | 19 | 9156253 | 4.90 | 0.17 | G/A |
| rs9156264 | δ-Toc content | Harbin | 19 | 9156264 | 5.44 | 0.16 | G/A |
| rs9386162 | δ-Toc content | Harbin | 19 | 9386162 | 4.87 | 0.36 | A/G |
| rs9479779 | δ-Toc content | Harbin | 19 | 9479779 | 4.04 | 0.37 | A/G |
| rs9530043 | δ-Toc content | Harbin | 19 | 9530043 | 4.57 | 0.35 | A/G |
| rs9566892 | δ-Toc content | Harbin | 19 | 9566892 | 4.31 | 0.36 | G/A |
| rs9567256 | δ-Toc content | Harbin | 19 | 9567256 | 4.40 | 0.36 | T/C |
| rs9576679 | δ-Toc content | Harbin | 19 | 9576679 | 5.04 | 0.38 | A/G |
| rs9613330 | δ-Toc content | Harbin | 19 | 9613330 | 4.04 | 0.37 | A/G |
| rs9615952 | δ-Toc content | Harbin | 19 | 9615952 | 4.35 | 0.36 | T/A |
| rs10359107 | δ-Toc content | Harbin | 19 | 10359107 | 4.04 | 0.37 | A/G |
| rs10866180 | δ-Toc content | Harbin | 19 | 10866180 | 4.35 | 0.35 | A/G |
| rs10866555 | δ-Toc content | Harbin | 19 | 10866555 | 4.33 | 0.37 | T/A |
| rs11539773 | δ-Toc content | Harbin | 19 | 11539773 | 4.04 | 0.37 | T/C |
| rs11732866 | δ-Toc content | Harbin | 19 | 11732866 | 4.28 | 0.39 | A/G |
| rs11838628 | δ-Toc content | Harbin | 19 | 11838628 | 4.04 | 0.37 | A/C |
| rs12543529 | δ-Toc content | Harbin | 19 | 12543529 | 4.08 | 0.37 | C/T |
| rs12982938 | δ-Toc content | Harbin | 19 | 12982938 | 4.04 | 0.37 | A/T |
| rs37558520 | Total-Toc content | Liaoning | 19 | 37558520 | 4.09 | 0.15 | T/C |
| rs47221569 | α-Toc content | Harbin | 19 | 47221569 | 4.33 | 0.43 | A/T |
| rs34774232 | δ-Toc content | Harbin | 20 | 34774232 | 4.82 | 0.23 | A/G |
| rs35655050 | δ-Toc content | Liaoning | 20 | 35655050 | 4.14 | 0.21 | T/C |
| rs35815938 | δ-Toc content | Liaoning | 20 | 35815938 | 4.12 | 0.17 | T/C |
| rs2162876 | α-Toc content | Harbin | 1 | 2162876 | 4.33 | 0.05 | A/C |

| **Table S4** Peak SNP associated with the Toc content identified by GWAS using the MLM model. | | | | | | | |
| --- | --- | --- | --- | --- | --- | --- | --- |
| SNP | Traits | Environment | Chr. | Position | -log10（P） | MAF | Allele |
| rs9337368 | δ-Toc content | Harbin | 2 | 9337368 | 4.03 | 0.06 | A/T |
| rs33956629 | α-Toc content | Harbin | 5 | 33956629 | 4.37 | 0.02 | T/C |
| rs7218697 | α-Toc content | Harbin | 7 | 7218697 | 4.02 | 0.04 | C/T |
| rs1834346 | α-Toc content | Harbin | 8 | 1834346 | 4.16 | 0.03 | A/T |
| rs19310064 | α-Toc content | Harbin | 8 | 19310064 | 9.02 | 0.01 | A/C |
| rs31044180 | γ-Toc content | Jilin | 9 | 31044180 | 4.30 | 0.08 | G/T |
| rs49928375 | α-Toc content | Harbin | 10 | 49928375 | 4.91 | 0.06 | G/T |
| rs9782629 | γ-Toc content | Harbin | 14 | 9782629 | 4.71 | 0.07 | G/T |
| rs19530677 | γ-Toc content | Harbin | 16 | 19530677 | 5.28 | 0.05 | T/A |
| rs2780598 | Total-Toc content | Harbin | 17 | 2780598 | 5.20 | 0.02 | C/G |
| rs14593163 | Total-Toc content | Harbin | 17 | 14593163 | 5.35 | 0.20 | T/G |
| rs24979561 | α-Toc content | Harbin | 17 | 24979561 | 5.87 | 0.07 | G/A |
| rs19903533 | δ-Toc content | Harbin | 18 | 19903533 | 4.67 | 0.03 | G/T |
| rs19962490 | δ-Toc content | Harbin | 18 | 19962490 | 5.42 | 0.02 | T/C |
|  | Total-Toc content | Harbin |  |  | 4.39 |  |  |
| rs24865620 | Total-Toc content | Jilin | 18 | 24865620 | 4.01 | 0.05 | C/T |
| rs53062844 | α-Toc content | Liaoning | 18 | 53062844 | 4.87 | 0.08 | G/T |
| rs6204830 | α-Toc content | Liaoning | 19 | 6204830 | 4.10 | 0.09 | T/G |
| rs12074736 | δ-Toc content | Jilin | 19 | 12074736 | 4.14 | 0.17 | C/A |

| **Table S5** Peak SNP associated with the Toc content identified by GWAS using the CMLM model. | | | | | | | |
| --- | --- | --- | --- | --- | --- | --- | --- |
| SNP | Traits | Environment | Chr. | Position | -log10（P） | MAF | Allele |
| rs2162876 | α-Toc content | Harbin | 1 | 2162876 | 4.33 | 0.05 | A/C |
| rs5437879 | α-Toc content | Harbin | 1 | 5437879 | 4.19 | 0.06 | C/A |
| rs23631390 | γ-Toc content | Harbin | 1 | 23631390 | 5.44 | 0.05 | G/A |
| rs36048309 | γ-Toc content | Jilin | 1 | 36048309 | 4.04 | 0.07 | A/G |
| rs9454083 | α-Toc content | Jilin | 2 | 9454083 | 4.39 | 0.05 | G/A |
| rs9299728 | α-Toc content | Liaoning | 3 | 9299728 | 4.13 | 0.02 | C/T |
| rs12579802 | α-Toc content | Harbin | 4 | 12579802 | 5.59 | 0.02 | G/A |
| rs24977653 | α-Toc content | Harbin | 4 | 24977653 | 5.96 | 0.07 | G/T |
| rs27955520 | α-Toc content | Harbin | 4 | 27955520 | 5.06 | 0.01 | T/G |
| rs48460348 | δ-Toc content | Harbin | 4 | 48460348 | 4.02 | 0.01 | T/G |
|  | Total-Toc  content | Harbin |  |  | 5.16 |  |  |
| rs17449289 | α-Toc content | Harbin | 6 | 17449289 | 4.31 | 0.05 | T/C |
| rs51277551 | α-Toc content | Harbin | 6 | 51277551 | 4.29 | 0.01 | T/A |
| rs7089772 | α-Toc content | Harbin | 7 | 7089772 | 4.57 | 0.03 | C/A |
| rs7182452 | α-Toc content | Harbin | 7 | 7182452 | 4.57 | 0.03 | T/C |
| rs7195097 | α-Toc content | Harbin | 7 | 7195097 | 4.90 | 0.04 | G/A |
| rs19310064 | α-Toc content | Harbin | 8 | 19310064 | 9.02 | 0.01 | A/C |
| rs46769362 | γ-Toc content | Harbin | 8 | 46769362 | 4.23 | 0.05 | G/T |
| rs9047280 | α-Toc content | Harbin | 9 | 9047280 | 5.26 | 0.04 | G/T |
| rs3842394 | α-Toc content | Liaoning | 10 | 3842394 | 4.30 | 0.16 | T/A |
| rs8254757 | δ-Toc content | Harbin | 10 | 8254757 | 4.00 | 0.07 | C/T |
| rs40007289 | δ-Toc content | Liaoning | 10 | 40007289 | 4.74 | 0.03 | T/C |
| rs49928375 | α-Toc content | Harbin | 10 | 49928375 | 5.04 | 0.06 | G/T |
| rs15936090 | α-Toc content | Harbin | 11 | 15936090 | 5.49 | 0.01 | A/G |
| rs17125409 | α-Toc content | Jilin | 12 | 17125409 | 5.21 | 0.07 | C/A |
| rs29183316 | α-Toc content | Jilin | 12 | 29183316 | 4.18 | 0.48 | G/A |
| rs43578642 | α-Toc content | Jilin | 13 | 43578642 | 4.28 | 0.08 | C/A |
| rs9782629 | γ-Toc content | Harbin | 14 | 9782629 | 5.63 | 0.07 | G/T |
| rs19530677 | γ-Toc content | Harbin | 16 | 19530677 | 7.33 | 0.05 | T/A |
|  | Total-Toc content | Harbin |  |  | 6.67 |  |  |
| rs2793608 | δ-Toc content | Jilin | 17 | 2793608 | 4.57 | 0.05 | G/T |
| rs24979561 | α-Toc content | Harbin | 17 | 24979561 | 5.87 | 0.07 | G/A |
| rs40233760 | α-Toc content | Jilin | 17 | 40233760 | 4.10 | 0.08 | G/T |
| rs3764631 | α-Toc content | Harbin | 18 | 3764631 | 4.40 | 0.08 | C/A |
| rs30693197 | α-Toc content | Harbin | 18 | 30693197 | 4.00 | 0.07 | C/A |
| rs49453339 | δ-Toc content | Harbin | 18 | 49453339 | 4.14 | 0.45 | A/G |
| rs53062844 | α-Toc content | Liaoning | 18 | 53062844 | 4.91 | 0.08 | G/T |
| rs31881460 | α-Toc content | Harbin | 19 | 31881460 | 5.02 | 0.09 | G/T |
| rs37037897 | α-Toc content | Liaoning | 19 | 37037897 | 4.03 | 0.10 | G/T |
| rs19168885 | α-Toc content | Liaoning | 20 | 19168885 | 4.05 | 0.05 | C/A |
| rs45306899 | δ-Toc content | Jilin | 20 | 45306899 | 4.01 | 0.45 | A/G |
| rs45306912 | δ-Toc content | Jilin | 20 | 45306912 | 4.11 | 0.46 | C/A |
| rs2162876 | α-Toc content | Harbin | 1 | 2162876 | 4.33 | 0.05 | A/C |

| **Table S6** Peak SNP associated with the Toc content identified by GWAS using the BLINK model. | | | | | | | |
| --- | --- | --- | --- | --- | --- | --- | --- |
| SNP | Traits | Environment | Chr. | Position | -log10（P） | MAF | Allele |
| rs43173548 | γ-Toc content | Jilin | 1 | 43173548 | 4.13 | 0.46 | C/T |
| rs4764653 | α-Toc content | Harbin | 2 | 4764653 | 6.29 | 0.17 | A/T |
| rs9337368 | δ-Toc content | Harbin | 2 | 9337368 | 6.48 | 0.06 | A/T |
| rs33852733 | δ-Toc content | Jilin | 3 | 33852733 | 6.77 | 0.04 | A/G |
| rs37890724 | δ-Toc content | Jilin | 3 | 37890724 | 7.41 | 0.22 | G/T |
| rs39077086 | α-Toc content | Harbin | 3 | 39077086 | 8.06 | 0.43 | C/T |
| rs39895210 | Total-Toc content | Liaoning | 3 | 39895210 | 4.15 | 0.27 | G/A |
| rs5793214 | α-Toc content | Liaoning | 4 | 5793214 | 4.03 | 0.09 | C/A |
| rs30444246 | Total-Toc content | Harbin | 4 | 30444246 | 4.77 | 0.05 | G/T |
| rs46089902 | α-Toc content | Jilin | 4 | 46089902 | 5.02 | 0.35 | T/C |
| rs46672009 | α-Toc content | Jilin | 4 | 46672009 | 6.98 | 0.16 | G/T |
| rs49628199 | δ-Toc content | Harbin | 6 | 49628199 | 6.58 | 0.20 | T/C |
| rs19310064 | α-Toc content | Harbin | 8 | 19310064 | 11.84 | 0.01 | A/C |
| rs20242356 | γ-Toc content | Jilin | 9 | 20242356 | 4.04 | 0.50 | A/C |
| rs7543892 | δ-Toc content | Jilin | 10 | 7543892 | 7.07 | 0.07 | T/G |
| rs17125409 | α-Toc content | Harbin | 12 | 17125409 | 6.09 | 0.07 | C/A |
|  | α-Toc content | Jilin |  |  | 10.27 |  |  |
| rs18678328 | δ-Toc content | Jilin | 12 | 18678328 | 4.07 | 0.10 | G/T |
| rs33250256 | α-Toc content | Jilin | 12 | 33250256 | 5.27 | 0.32 | G/C |
| rs16207451 | δ-Toc content | Jilin | 13 | 16207451 | 5.67 | 0.13 | T/C |
| rs19241701 | α-Toc content | Liaoning | 13 | 19241701 | 6.01 | 0.10 | G/T |
| rs1605554 | γ-Toc content | Harbin | 14 | 1605554 | 4.23 | 0.16 | C/T |
| rs9782629 | γ-Toc content | Harbin | 14 | 9782629 | 7.27 | 0.07 | G/T |
| rs22145998 | Total-Toc content | Jilin | 14 | 22145998 | 4.01 | 0.10 | G/T |
| rs16976090 | α-Toc content | Liaoning | 15 | 16976090 | 7.16 | 0.22 | T/C |
| rs38161412 | α-Toc content | Harbin | 15 | 38161412 | 6.82 | 0.28 | A/G |
| rs12888642 | α-Toc content | Liaoning | 16 | 12888642 | 7.52 | 0.20 | C/A |
| rs19530677 | γ-Toc content | Harbin | 16 | 19530677 | 9.16 | 0.05 | T/A |
|  | Total-Toc content | Harbin |  |  | 4.23 |  |  |
| rs25739207 | α-Toc content | Liaoning | 16 | 25739207 | 7.36 | 0.11 | G/A |
| rs2793608 | δ-Toc content | Jilin | 17 | 2793608 | 7.01 | 0.05 | G/T |
| rs14108110 | Total-Toc content | Harbin | 17 | 14108110 | 4.74 | 0.19 | T/C |
| rs14593134 | Total-Toc content | Harbin | 17 | 14593134 | 4.32 | 0.18 | A/G |
| rs14593163 | δ-Toc content | Harbin | 17 | 14593163 | 6.42 | 0.19 | T/G |
|  | Total-Toc content | Harbin |  |  | 4.56 |  |  |
| rs14680873 | Total-Toc content | Harbin | 17 | 14680873 | 4.51 | 0.19 | A/G |
| rs24979561 | α-Toc content | Harbin | 17 | 24979561 | 7.86 | 0.07 | G/A |
| rs40233760 | α-Toc content | Jilin | 17 | 40233760 | 7.68 | 0.08 | G/T |
| rs5680781 | γ-Toc content | Jilin | 18 | 5680781 | 4.62 | 0.08 | G/T |
|  | Total-Toc content | Jilin |  |  | 5.61 |  |  |
| rs8479815 | δ-Toc content | Jilin | 18 | 8479815 | 4.14 | 0.13 | C/T |
| rs17266245 | γ-Toc content | Jilin | 18 | 17266245 | 4.31 | 0.13 | T/G |
| rs53062844 | α-Toc content | Liaoning | 18 | 53062844 | 12.82 | 0.08 | G/T |
| rs8720462 | δ-Toc content | Harbin | 19 | 8720462 | 7.38 | 0.17 | G/A |
| rs8970462 | δ-Toc content | Jilin | 19 | 8970462 | 5.34 | 0.16 | G/A |

| **Table S7** Peak SNP associated with the Toc content identified by GWAS using the FarmCPU model. | | | | | | | |
| --- | --- | --- | --- | --- | --- | --- | --- |
| SNP | Traits | Environment | Chr. | Position | -log10（P） | MAF | Allele |
| rs9337368 | δ-Toc content | Harbin | 2 | 9337368 | 6.15 | 0.06 | A/T |
| rs10763457 | δ-Toc content | Harbin | 3 | 10763457 | 7.68 | 0.13 | C/A |
| rs39895210 | Total-Toc content | Liaoning | 3 | 39895210 | 4.53 | 0.27 | G/A |
| rs36602236 | α-Toc content | Harbin | 4 | 36602236 | 4.08 | 0.08 | G/T |
| rs11236537 | δ-Toc content | Harbin | 5 | 11236537 | 4.04 | 0.09 | G/A |
| rs22876354 | α-Toc content | Harbin | 5 | 22876354 | 4.64 | 0.07 | G/A |
| rs2960931 | δ-Toc content | Liaoning | 6 | 2960931 | 4.45 | 0.19 | G/A |
| rs9318903 | α-Toc content | Harbin | 8 | 9318903 | 5.44 | 0.50 | A/G |
| rs18596407 | Total-Toc  content | Harbin | 9 | 18596407 | 4.76 | 0.11 | C/A |
| rs31044180 | α-Toc content | Jilin | 9 | 31044180 | 4.43 | 0.08 | G/T |
|  | γ-Toc content | Jilin |  |  | 5.15 |  |  |
|  | Total-Toc content | Jilin |  |  | 4.22 |  |  |
| rs7543892 | δ-Toc content | Jilin | 10 | 7543892 | 4.55 | 0.06 | T/G |
| rs49928375 | α-Toc content | Harbin | 10 | 49928375 | 4.67 | 0.06 | G/T |
| rs11885640 | α-Toc content | Harbin | 11 | 11885640 | 5.41 | 0.29 | C/T |
| rs17125409 | α-Toc content | Harbin | 12 | 17125409 | 7.63 | 0.07 | C/A |
| rs330000 | δ-Toc content | Liaoning | 13 | 330000 | 4.56 | 0.17 | G/A |
| rs9782629 | γ-Toc content | Harbin | 14 | 9782629 | 4.89 | 0.07 | G/T |
| rs13678290 | Total- Toc content | Harbin | 14 | 13678290 | 4.35 | 0.39 | G/A |
| rs28417306 | δ-Toc content | Harbin | 14 | 28417306 | 6.15 | 0.05 | T/C |
| rs12265057 | δ-Toc content | Harbin | 15 | 12265057 | 6.29 | 0.21 | T/C |
| rs23102196 | α-Toc content | Harbin | 15 | 23102196 | 5.35 | 0.11 | C/A |
| rs39722884 | α-Toc content | Harbin | 15 | 39722884 | 4.73 | 0.21 | G/A |
| rs19530677 | γ-Toc content | Harbin | 16 | 19530677 | 6.10 | 0.05 | T/A |
| rs14593163 | Total-Toc content | Harbin | 17 | 14593163 | 7.49 | 0.20 | T/G |
| rs24979561 | α-Toc content | Harbin | 17 | 24979561 | 7.56 | 0.07 | G/A |
| rs588498 | α-Toc content | Liaoning | 18 | 588498 | 4.26 | 0.43 | G/A |
| rs13237620 | δ-Toc content | Harbin | 18 | 13237620 | 4.26 | 0.15 | A/T |
| rs37126946 | δ-Toc content | Harbin | 18 | 37126946 | 5.65 | 0.04 | C/A |
| rs53062844 | α-Toc content | Liaoning | 18 | 53062844 | 5.73 | 0.08 | G/T |
| rs8393139 | Total-Toc content | Harbin | 19 | 8393139 | 4.77 | 0.12 | A/G |
| rs8720462 | δ-Toc content | Harbin | 19 | 8720462 | 4.37 | 0.17 | G/A |
|  | δ-Toc content | Liaoning |  |  | 4.48 |  |  |
| rs37558520 | Total-Toc content | Liaoning | 19 | 37558520 | 4.06 | 0.15 | T/C |
| rs34774232 | δ-Toc content | Harbin | 20 | 34774232 | 4.72 | 0.23 | A/G |
| rs35655050 | δ-Toc content | Liaoning | 20 | 35655050 | 4.14 | 0.21 | T/C |
| rs35815938 | δ-Toc content | Liaoning | 20 | 35815938 | 4.14 | 0.17 | T/C |
| rs8970462 | δ-Toc content | Jilin | 19 | 8970462 | 5.34 | 0.16 | G/A |

| **Table S8** Peak SNP associated with the Toc content identified by GWAS using the 3VmrMLM model single-environment method. | | | | | | | | | | | | | | |
| --- | --- | --- | --- | --- | --- | --- | --- | --- | --- | --- | --- | --- | --- | --- |
| SNP | Traits | Environment | | | Chr. | | Position | | -log10（P） | | | r^2^(%) | Allele | |
| rs1967942 | α-Toc content | Harbin | | | 1 | | 1967942 | | 10.05 | | | 6.38 | C/A | |
| rs7167202 | γ-Toc content | Jilin | | | 1 | | 7167202 | | 5.13 | | | 3.33 | G/T | |
|  | Total-Toc content | Jilin | | |  | |  | | 6.43 | | | 6.38 |  | |
| rs37510038 | γ-Toc content | Liaoning | | | 1 | | 37510038 | | 7.72 | | | 4.79 | G/A | |
| rs40908486 | Total-Toc content | Liaoning | | | 1 | | 40908486 | | 4.56 | | | 2.92 | G/T | |
| rs41784197 | γ-Toc content | Jilin | | | 1 | | 41784197 | | 11.64 | | | 7.93 | T/C | |
| rs47986763 | δ-Toc content | Harbin | | | 1 | | 47986763 | | 5.81 | | | 5.47 | A/C | |
| rs54767936 | γ-Toc content | Harbin | | | 1 | | 54767936 | | 5.33 | | | 3.41 | T/G | |
| rs1634861 | γ-Toc content | Jilin | | | 2 | | 1634861 | | 6.08 | | | 2.56 | G/A | |
| rs3046670 | δ-Toc content | Liaoning | | | 2 | | 3046670 | | 4.99 | | | 4.95 | G/A | |
| rs13744031 | γ-Toc content | Harbin | | | 2 | | 13744031 | | 7.12 | | | 5.45 | G/C | |
| rs14033171 | δ-Toc content | Harbin | | | 2 | | 14033171 | | 5.52 | | | 6.00 | T/A | |
| rs39535378 | α-Toc content | Harbin | | | 2 | | 39535378 | | 7.11 | | | 4.42 | G/A | |
| rs47048006 | Total-Toc content | Liaoning | | | 2 | | 47048006 | | 7.04 | | | 5.99 | G/A | |
| rs3804102 | δ-Toc content | Liaoning | | | 3 | | 3804102 | | 8.65 | | | 3.37 | T/A | |
| rs5009043 | γ-Toc content | Harbin | | | 3 | | 5009043 | | 7.80 | | | 5.49 | C/A | |
| rs5818126 | γ-Toc content | Harbin | | | 3 | | 5818126 | | 6.14 | | | 4.85 | A/T | |
| rs6719191 | α-Toc content | Jilin | | | 3 | | 6719191 | | 4.52 | | | 2.89 | G/T | |
| rs28985136 | γ-Toc content | Jilin | | | 3 | | 28985136 | | 6.63 | | | 4.78 | C/A | |
| rs39895210 | Total-Toc content | Liaoning | | | 3 | | 39895210 | | 19.38 | | | 15.69 | G/A | |
| rs3023696 | γ-Toc content | Jilin | | | 4 | | 3023696 | | 13.08 | | | 5.34 | T/C | |
| rs4518367 | δ-Toc content | Liaoning | | | 4 | | 4518367 | | 7.47 | | | 4.99 | C/A | |
| rs48232808 | Total-Toc content | Jilin | | | 4 | | 48232808 | | 6.36 | | | 8.27 | T/G | |
| rs48514134 | δ-Toc content | Liaoning | | | 4 | | 48514134 | | 8.59 | | | 6.04 | A/G | |
| rs31884576 | α-Toc content | Jilin | | | 5 | | 31884576 | | 10.60 | | | 5.30 | A/G | |
| rs38068554 | Total-Toc content | Jilin | | | 5 | | 38068554 | | 11.59 | | | 4.99 | G/T | |
| rs2960931 | δ-Toc content | Liaoning | | | 6 | | 2960931 | | 10.47 | | | 6.56 | G/A | |
| rs6068605 | α-Toc content | Harbin | | | 6 | | 6068605 | | 23.70 | | | 5.68 | T/A | |
| rs15888622 | Total-Toc content | Liaoning | | | 6 | | 15888622 | | 6.25 | | | 5.34 | A/G | |
| rs16263376 | δ-Toc content | Jilin | | | 6 | | 16263376 | | 4.61 | | | 5.13 | G/T | |
| rs33602349 | δ-Toc content | Harbin | | | 6 | | 33602349 | | 10.34 | | | 7.51 | C/A | |
| rs9140707 | α-Toc content | Liaoning | | | 7 | | 9140707 | | 17.35 | | | 3.12 | G/T | |
| rs9985352 | Total-Toc content | Harbin | | | 7 | | 9985352 | | 6.99 | | | 1.97 | C/A | |
| rs31912929 | γ-Toc content | Liaoning | | | 7 | | 31912929 | | 5.46 | | | 5.49 | G/T | |
| rs36481334 | Total-Toc content | Jilin | | | 7 | | 36481334 | | 4.54 | | | 7.03 | G/T | |
| rs1588537 | Total-Toc content | Harbin | | | 8 | | 1588537 | | 16.79 | | | 6.02 | A/C | |
| rs9319265 | α-Toc content | Harbin | | | 8 | | 9319265 | | 5.28 | | | 3.36 | A/G | |
| rs13209021 | γ-Toc content | Liaoning | | | 8 | | 13209021 | | 16.46 | | | 7.83 | G/T | |
| rs18105573 | δ-Toc content | Jilin | | | 8 | | 18105573 | | 6.44 | | | 7.64 | A/G | |
| rs19332142 | α-Toc content | Liaoning | | | 8 | | 19332142 | | 4.45 | | | 3.06 | C/A | |
| rs45007284 | γ-Toc content | Jilin | | | 8 | | 45007284 | | 8.56 | | | 5.59 | G/T | |
| rs2511987 | Total-Toc content | Liaoning | | | 9 | | 2511987 | | 4.16 | | | 2.47 | C/T | |
| rs2669053 | γ-Toc content | Harbin | | | 9 | | 2669053 | | 16.30 | | | 14.16 | T/C | |
|  | Total-Toc content | Harbin | | |  | |  | | 12.13 | | | 14.09 |  | |
| rs31044180 | δ-Toc content | Jilin | | | 9 | | 31044180 | | 18.07 | | | 4.85 | G/T | |
| rs49333152 | Total-Toc content | Liaoning | | | 9 | | 49333152 | | 12.55 | | | 7.53 | G/T | |
| rs3763850 | α-Toc content | Liaoning | | | 10 | | 3763850 | | 9.56 | | | 4.76 | G/T | |
| rs13737322 | Total-Toc content | Harbin | | | 10 | | 13737322 | | 6.98 | | | 7.64 | G/A | |
| rs49928375 | α-Toc content | Harbin | | | 10 | | 49928375 | | 17.93 | | | 2.57 | G/T | |
| rs17062257 | Total-Toc content | Harbin | | | 11 | | 17062257 | | 8.51 | | | 3.71 | A/C | |
| rs1006049 | α-Toc content | Jilin | | | 12 | | 1006049 | | 7.18 | | | 4.83 | A/C | |
| rs21143057 | α-Toc content | Liaoning | | | 12 | | 21143057 | | 20.64 | | | 4.49 | G/A | |
| rs33250256 | α-Toc content | Jilin | | | 12 | | 33250256 | | 5.32 | | | 3.45 | G/C | |
| rs33687053 | γ-Toc content | Jilin | | | 12 | | 33687053 | | 17.90 | | | 6.00 | G/T | |
| rs330000 | δ-Toc content | Liaoning | | | 13 | | 330000 | | 9.65 | | | 6.45 | G/A | |
| rs5318148 | α-Toc content | Harbin | | | 13 | | 5318148 | | 6.69 | | | 4.32 | T/C | |
| rs14311945 | TotalToc- content | Harbin | | | 13 | | 14311945 | | 30.35 | | | 10.26 | G/T | |
| rs19607617 | γ-Toc content | Liaoning | | | 13 | | 19607617 | | 10.10 | | | 8.36 | G/T | |
| rs20380420 | γ-Toc content | Liaoning | | | 13 | | 20380420 | | 10.70 | | | 5.84 | T/A | |
| rs29588541 | γ-Toc content | Harbin | | | 13 | | 29588541 | | 6.27 | | | 4.23 | A/C | |
| rs29847711 | α-Toc content | Harbin | | | 13 | | 29847711 | | 4.63 | | | 2.94 | C/A | |
| rs43578642 | α-Toc content | Jilin | | | 13 | | 43578642 | | 20.41 | | | 5.33 | C/A | |
| rs29070102 | α-Toc content | Jilin | | | 14 | | 29070102 | | 32.86 | | | 5.73 | C/A | |
| rs11720920 | Total-Toc content | Liaoning | | | 15 | | 11720920 | | 8.42 | | | 7.06 | C/A | |
| rs13830686 | δ-Toc content | Jilin | | | 15 | | 13830686 | | 7.44 | | | 10.35 | A/G | |
| rs14449662 | δ-Toc content | Liaoning | | | 15 | | 14449662 | | 4.88 | | | 4.83 | A/G | |
| rs22341967 | δ-Toc content | Liaoning | | | 15 | | 22341967 | | 5.61 | | | 4.77 | T/G | |
| rs37798958 | Total-Toc content | Liaoning | | | 15 | | 37798958 | | 4.77 | | | 3.60 | C/A | |
| rs43000771 | γ-Toc content | Liaoning | | | 15 | | 43000771 | | 4.07 | | | 4.11 | C/T | |
| rs49527604 | γ-Toc content | Liaoning | | | 15 | | 49527604 | | 8.59 | | | 8.07 | T/G | |
| rs50565303 | α-Toc content | Jilin | | | 15 | | 50565303 | | 7.64 | | | 3.07 | C/T | |
| rs14826149 | α-Toc content | Jilin | | | 16 | | 14826149 | | 9.95 | | | 4.58 | G/T | |
| rs27969771 | Total-Toc content | Jilin | | | 16 | | 27969771 | | 4.38 | | | 5.66 | G/A | |
| rs36081826 | α-Toc content | Jilin | | | 16 | | 36081826 | | 13.47 | | | 4.28 | G/T | |
| rs37783217 | γ-Toc content | Liaoning | | | 16 | | 37783217 | | 6.11 | | | 4.68 | A/G | |
| rs4717651 | δ-Toc content | Liaoning | | | 17 | | 4717651 | | 5.70 | | | 4.70 | C/T | |
| rs12916838 | δ-Toc content | Harbin | | | 17 | | 12916838 | | 4.62 | | | 4.33 | C/G | |
| rs18969794 | γ-Toc content | Harbin | | | 17 | | 18969794 | | 5.25 | | | 2.91 | G/T | |
| rs24979561 | α-Toc content | Harbin | | | 17 | | 24979561 | | 18.72 | | | 3.45 | G/A | |
| rs37757098 | α-Toc content | Liaoning | | | 17 | | 37757098 | | 8.58 | | | 5.24 | G/A | |
| rs588498 | α-Toc content | Liaoning | | | 18 | | 588498 | | 4.53 | | | 3.43 | G/A | |
| rs5680781 | γ-Toc content | Jilin | | | 18 | | 5680781 | | 9.07 | | | 1.99 | G/T | |
| rs17266245 | γ-Toc content | Jilin | | | 18 | | 17266245 | | 16.18 | | | 7.07 | T/G | |
| rs20511904 | α-Toc content | Harbin | | | 18 | | 20511904 | | 13.42 | | | 9.62 | G/T | |
| rs20536038 | Total-Toc content | Harbin | | | 18 | | 20536038 | | 5.41 | | | 4.07 | A/G | |
| rs30553268 | α-Toc content | Harbin | | | 18 | | 30553268 | | 13.30 | | | 1.53 | G/T | |
| rs50030476 | Total-Toc content | Harbin | | | 18 | | 50030476 | | 13.89 | | | 6.24 | G/C | |
| rs1261520 | α-Toc content | Jilin | | | 19 | | 1261520 | | 12.41 | | | 6.37 | G/T | |
| rs5779917 | γ-Toc content | Harbin | | | 19 | | 5779917 | | 7.76 | | | 6.29 | G/T | |
| rs6204830 | α-Toc content | Liaoning | | | 19 | | 6204830 | | 15.42 | | | 4.21 | T/G | |
|  | α-Toc content | Jilin | | |  | |  | | 7.40 | | | 1.88 |  | |
| rs8720462 | δ-Toc content | Harbin | | | 19 | | 8720462 | | 5.61 | | | 3.75 | G/A | |
| rs8917233 | δ-Toc content | Jilin | | | 19 | | 8917233 | | 13.78 | | | 11.49 | C/A | |
| rs10866555 | δ-Toc content | Harbin | | | 19 | | 10866555 | | 6.14 | | | 6.75 | T/A | |
| rs24490221 | γ-Toc content | Harbin | | | 19 | | 24490221 | | 6.35 | | | 5.04 | A/T | |
| rs26086587 | α-Toc content | Liaoning | | | 19 | | 26086587 | | 12.33 | | | 5.57 | G/T | |
| rs35039015 | δ-Toc content | Jilin | | | 19 | | 35039015 | | 11.00 | | | 4.89 | C/A | |
| rs37558520 | α-Toc content | Liaoning | | | 19 | | 37558520 | | 9.34 | | | 4.07 | T/C | |
| rs48042885 | α-Toc content | Harbin | | | 19 | | 48042885 | | 9.27 | | | 5.24 | A/T | |
| rs3023398 | γ-Toc content | Jilin | | | 20 | | 3023398 | | 5.53 | | | 3.48 | A/G | |
| rs8618394 | γ-Toc content | Harbin | | | 20 | | 8618394 | | 4.43 | | | 3.10 | C/A | |
| rs36950954 | Total-Toc content | Liaoning | | | 20 | | 36950954 | | 7.61 | | | 3.96 | C/T | |
| rs38200036 | Total-Toc content | Jilin | | | 20 | | 38200036 | | 9.20 | | | 7.52 | C/A | |
| rs46814888 | δ-Toc content | Harbin | | | 20 | | 46814888 | | 8.64 | | | 5.43 | T/G | |
| **Table S9** Peak SNP associated with the Toc content identified by GWAS using QTL detection of 3VmrMLM multiple-environment method. | | | | | | | | | | | | | |  |
| Marker | Traits | | Chr. | Position | | r^2^(%) | | -log10（P） | | Allele | significance | | |  |
| rs5957934 | δ-Toc content | | 1 | 5957934 | | 0.74 | | 4.79 | | A/G | SUG | | |  |
| rs7167202 | Total- content | | 1 | 7167202 | | 2.19 | | 16.28 | | G/T | SIG | | |  |
| rs33810082 | α-Toc content | | 1 | 33810082 | | 1.79 | | 10.16 | | A/C | SIG | | |  |
| rs41784197 | γ-Toc content | | 1 | 41784197 | | 2.77 | | 12.32 | | T/C | SIG | | |  |
| rs54988794 | γ-Toc content | | 1 | 54988794 | | 2.72 | | 9.91 | | T/A | SIG | | |  |
| rs9337368 | δ-Toc content | | 2 | 9337368 | | 1.12 | | 6.33 | | A/T | SIG | | |  |
| rs14558036 | γ-Toc content | | 3 | 14558036 | | 1.14 | | 5.32 | | G/T | SUG | | |  |
| rs15344256 | α-Toc content | | 3 | 15344256 | | 0.69 | | 4.56 | | C/G | SUG | | |  |
| rs38585708 | α-Toc content | | 3 | 38585708 | | 1.91 | | 10.77 | | C/T | SIG | | |  |
| rs23248953 | Total-Toc content | | 4 | 23248953 | | 2.59 | | 7.10 | | G/C | SIG | | |  |
| rs46089902 | α-Toc content | | 4 | 46089902 | | 3.11 | | 16.84 | | T/C | SIG | | |  |
| rs3776281 | Total- content | | 5 | 3776281 | | 1.82 | | 4.36 | | G/T | SUG | | |  |
| rs2960931 | δ-Toc content | | 6 | 2960931 | | 1.13 | | 10.45 | | G/A | SIG | | |  |
| rs33602349 | δ-Toc content | | 6 | 33602349 | | 0.83 | | 4.45 | | C/A | SUG | | |  |
| rs1125901 | γ-Toc content | | 7 | 1125901 | | 1.09 | | 5.43 | | T/C | SUG | | |  |
| rs9140707 | α-Toc content | | 7 | 9140707 | | 1.64 | | 33.30 | | G/T | SIG | | |  |
| rs1834346 | Total- content | | 8 | 1834346 | | 2.84 | | 11.02 | | A/T | SIG | | |  |
| rs18105573 | δ-Toc content | | 8 | 18105573 | | 0.91 | | 5.58 | | A/G | SUG | | |  |
| rs2623586 | δ-Toc content | | 10 | 2623586 | | 1.15 | | 6.42 | | G/T | SIG | | |  |
| rs7543892 | δ-Toc content | | 10 | 7543892 | | 0.83 | | 11.13 | | T/G | SIG | | |  |
| rs40595691 | γ-Toc content | | 10 | 40595691 | | 1.10 | | 4.35 | | C/T | SUG | | |  |
|  | Total-Toc content | |  |  | | 3.07 | | 7.34 | |  | SIG | | |  |
| rs17125409 | α-Toc content | | 12 | 17125409 | | 2.28 | | 46.05 | | C/A | SIG | | |  |
| rs30745948 | α-Toc content | | 12 | 30745948 | | 1.20 | | 6.36 | | G/T | SIG | | |  |
| rs330000 | δ-Toc content | | 13 | 330000 | | 0.52 | | 4.35 | | G/A | SUG | | |  |
| rs11500692 | δ-Toc content | | 13 | 11500692 | | 1.73 | | 19.05 | | C/T | SIG | | |  |
| rs12489028 | α-Toc content | | 13 | 12489028 | | 1.17 | | 10.29 | | G/T | SIG | | |  |
| rs1605554 | γ-Toc content | | 14 | 1605554 | | 0.93 | | 6.44 | | C/T | SIG | | |  |
| rs1729555 | γ-Toc content | | 14 | 1729555 | | 2.53 | | 9.54 | | G/A | SIG | | |  |
| rs7006046 | γ-Toc content | | 14 | 7006046 | | 1.92 | | 10.65 | | G/A | SIG | | |  |
| rs22683352 | γ-Toc content | | 14 | 22683352 | | 2.35 | | 8.71 | | T/C | SIG | | |  |
| rs25004545 | α-Toc content | | 14 | 25004545 | | 0.56 | | 4.04 | | G/T | SUG | | |  |
| rs12107100 | δ-Toc content | | 15 | 12107100 | | 1.42 | | 14.48 | | T/C | SIG | | |  |
| rs14340230 | δ-Toc content | | 15 | 14340230 | | 2.64 | | 14.25 | | A/G | SIG | | |  |
| rs17568067 | α-Toc content | | 15 | 17568067 | | 1.73 | | 13.65 | | G/A | SIG | | |  |
| rs35823452 | Total-Toc content | | 15 | 35823452 | | 2.87 | | 7.36 | | G/T | SIG | | |  |
| rs36546835 | δ-Toc content | | 15 | 36546835 | | 0.98 | | 5.18 | | C/T | SUG | | |  |
| rs37196130 | δ-Toc content | | 15 | 37196130 | | 0.51 | | 4.08 | | T/C | SUG | | |  |
| rs43000771 | Total-Toc content | | 15 | 43000771 | | 1.75 | | 4.57 | | C/T | SUG | | |  |
| rs9992254 | α-Toc content | | 16 | 9992254 | | 1.65 | | 8.67 | | T/A | SIG | | |  |
| rs28496951 | Total- content | | 16 | 28496951 | | 2.27 | | 20.54 | | G/A | SIG | | |  |
| rs7334748 | δ-Toc content | | 17 | 7334748 | | 0.36 | | 3.63 | | A/G | SUG | | |  |
| rs8202476 | γ-Toc content | | 17 | 8202476 | | 1.62 | | 15.90 | | G/T | SIG | | |  |
| rs13706638 | δ-Toc content | | 17 | 13706638 | | 2.06 | | 12.12 | | A/T | SIG | | |  |
| rs17266245 | γ-Toc content | | 18 | 17266245 | | 1.39 | | 11.10 | | T/G | SIG | | |  |
| rs45649502 | γ-Toc content | | 18 | 45649502 | | 1.17 | | 8.22 | | G/T | SIG | | |  |
| rs53062844 | α-Toc content | | 18 | 53062844 | | 1.57 | | 23.60 | | G/T | SIG | | |  |
| rs55570229 | Total-Toc content | | 18 | 55570229 | | 3.56 | | 8.59 | | T/C | SIG | | |  |
| rs55790524 | α-Toc content | | 18 | 55790524 | | 0.89 | | 5.15 | | G/T | SUG | | |  |
| rs7985649 | γ-Toc content | | 19 | 7985649 | | 3.01 | | 11.71 | | G/T | SIG | | |  |
| rs8720462 | δ-Toc content | | 19 | 8720462 | | 0.59 | | 4.01 | | G/A | SUG | | |  |
| rs47303195 | α-Toc content | | 19 | 47303195 | | 1.67 | | 10.34 | | T/C | SIG | | |  |
| rs29979841 | γ-Toc content | | 20 | 29979841 | | 1.85 | | 9.68 | | G/A | SIG | | |  |
| rs34774232 | δ-Toc content | | 20 | 34774232 | | 1.80 | | 13.07 | | A/G | SIG | | |  |
| rs35562231 | γ-Toc content | | 20 | 35562231 | | 1.77 | | 9.48 | | A/T | SIG | | |  |
| rs35815938 | δ-Toc content | | 20 | 35815938 | | 1.08 | | 10.32 | | T/C | SIG | | |  |
| rs42885365 | γ-Toc content | | 20 | 42885365 | | 1.68 | | 6.70 | | G/T | SIG | | |  |
| rs46814888 | δ-Toc content | | 20 | 46814888 | | 0.87 | | 8.70 | | T/G | SIG | | |  |

| **Table S10** Peak SNP associated with the Toc content identified by GWAS using QEI detection of 3VmrMLM multiple-environment method. | | | | | | | |
| --- | --- | --- | --- | --- | --- | --- | --- |
| Marker | Traits | Chr. | Position | r^2^(%) | -log10（P） | Allele | significance |
| rs12447869 | γ-Toc content | 1 | 12447869 | 1.50 | 4.44 | G/C | SUG |
| rs38321290 | α-Toc content | 1 | 38321290 | 4.92 | 22.17 | C/A | SIG |
| rs37739787 | α-Toc content | 2 | 37739787 | 1.56 | 6.85 | G/T | SIG |
| rs2799329 | α-Toc content | 6 | 2799329 | 4.16 | 19.20 | G/T | SIG |
| rs2669053 | γ-Toc content | 9 | 2669053 | 3.61 | 11.49 | T/C | SIG |
|  | Total-Toc content |  |  | 2.97 | 5.23 |  | SUG |
| rs50270401 | γ-Toc content | 10 | 50270401 | 7.64 | 23.87 | G/T | SIG |
| rs29527738 | δ-Toc content | 11 | 29527738 | 4.97 | 21.84 | T/G | SIG |
| rs9782629 | γ-Toc content | 14 | 9782629 | 4.56 | 15.98 | G/T | SIG |
|  | Total-Toc content |  |  | 9.00 | 18.12 |  | SIG |
| rs19530677 | γ-Toc content | 16 | 19530677 | 10.64 | 32.05 | T/A | SIG |
| rs36745771 | δ-Toc content | 16 | 36745771 | 1.51 | 6.44 | T/C | SIG |
| rs49948296 | δ-Toc content | 18 | 49948296 | 1.27 | 5.41 | C/T | SUG |
| rs55080151 | Total- content | 18 | 55080151 | 2.88 | 5.14 | C/T | SUG |
| rs5779917 | α-Toc content | 19 | 5779917 | 2.01 | 8.90 | G/T | SIG |

| **Table S11** Candidate genes in the flanking regions of peak SNPs of two group. | | | | | | | | | |
| --- | --- | --- | --- | --- | --- | --- | --- | --- | --- |
| Peak SNP | Chr. | Physical position (bp) | | Gene ID | Start Position | | Stop Position | Functional annotation | |
| rs39895210 | 3 | 39895210 | | *Glyma.03G186200* | 39791040 | | 39795337 | RAB GTPase homolog C2A | |
|  |  |  | | *Glyma.03G186500* | 39817953 | | 39826834 | Transducin family protein / WD-40 repeat family protein | |
|  |  |  | | *Glyma.03G186600* | 39827357 | | 39828692 | expansin A9 | |
|  |  |  | | *Glyma.03G186700* | 39832690 | | 39839887 | Tudor/PWWP/MBT superfamily protein | |
|  |  |  | | *Glyma.03G186800* | 39842435 | | 39843916 | ARM repeat superfamily protein | |
|  |  |  | | *Glyma.03G186900* | 39848060 | | 39849803 | UDP-glucosyl transferase 73C2 | |
|  |  |  | | *Glyma.03G187000* | 39852803 | | 39854986 | UDP-glucosyl transferase 73C1 | |
|  |  |  | | *Glyma.03G187100* | 39856018 | | 39858175 | don-glucosyltransferase 1 | |
|  |  |  | | *Glyma.03G187200* | 39860553 | | 39862188 | don-glucosyltransferase 1 | |
|  |  |  | | *Glyma.03G187300* | 39863823 | | 39865741 | UDP-glucosyl transferase 73C7 | |
|  |  |  | | *Glyma.03G187400* | 39868285 | | 39869751 | don-glucosyltransferase 1 | |
|  |  |  | | *Glyma.03G187500* | 39873967 | | 39875695 | UDP-glucosyl transferase 73C1 | |
|  |  |  | | *Glyma.03G187700* | 39882427 | | 39884819 | UDP-glucosyl transferase 73C2 | |
|  |  |  | | *Glyma.03G187900* | 39902224 | | 39905432 | 2-oxoglutarate (2OG) and Fe(II)-dependent oxygenase superfamily protein | |
|  |  |  | | *Glyma.03G188100* | 39936237 | | 39945018 | Modifier of rudimentary (Mod(r)) protein | |
|  |  |  | | *Glyma.03G188300* | 39945140 | | 39946006 | Pollen Ole e 1 allergen and extensin family protein | |
| rs2960931 | 6 | 2960931 | | *Glyma.06G037300* | 2861369 | | 2870869 | uroporphyrinogen-III synthase family protein | |
|  |  |  | | *Glyma.06G037800* | 2894530 | | 2896325 | P-loop containing nucleoside triphosphate hydrolases superfamily protein | |
|  |  |  | | *Glyma.06G037900* | 2902002 | | 2905685 | phospholipase A 2A | |
|  |  |  | | *Glyma.06G038000* | 2908879 | | 2910318 | Alpha/beta-Hydrolases superfamily protein | |
|  |  |  | | *Glyma.06G038100* | 2912312 | | 2913301 | Plant protein of unknown function (DUF639) | |
|  |  |  | | *Glyma.06G038200* | 2914749 | | 2915579 | Histone superfamily protein | |
|  |  |  | | *Glyma.06G038600* | 2930065 | | 2934257 | Protein of unknown function DUF829, transmembrane 53 | |
|  |  |  | | *Glyma.06G038700* | 2935243 | | 2937127 | NAD(P)-binding Rossmann-fold superfamily protein | |
|  |  |  | | *Glyma.06G038800* | 2937412 | | 2937772 | Ribosomal protein L14 | |
|  |  |  | | *Glyma.06G039000* | 2949704 | | 2954653 | Protein phosphatase 2C family protein | |
|  |  |  | | *Glyma.06G039100* | 2963434 | | 2964779 | BRI1 kinase inhibitor 1 | |
|  |  |  | | *Glyma.06G039300* | 2981077 | | 2981364 | calmodulin like 37 | |
|  |  |  | | *Glyma.06G039400* | 2983188 | | 2983676 | calmodulin like 37 | |
|  |  |  | | *Glyma.06G039500* | 2986298 | | 2990124 | Tic22-like family protein | |
|  |  |  | | *Glyma.06G039800* | 3003277 | | 3006341 | Vacuolar import/degradation, Vid27-related protein | |
|  |  |  | | *Glyma.06G040000* | 3012293 | | 3014635 | Tetratricopeptide repeat (TPR)-like superfamily protein | |
|  |  |  | | *Glyma.06G040100* | 3015227 | | 3020272 | Polyketide cyclase / dehydrase and lipid transport protein | |
|  |  |  | | *Glyma.06G040300* | 3025530 | | 3029879 | Protein of unknown function (DUF3223) | |
|  |  |  | | *Glyma.06G040400* | 3041657 | | 3047267 | abscisic acid responsive elements-binding factor 2 | |
|  |  |  | | *Glyma.06G040500* | 3042471 | | 3042767 | abscisic acid responsive elements-binding factor 2 | |
|  |  |  | | *Glyma.06G040800* | 3066046 | | 3067280 | basic helix-loop-helix (bHLH) DNA-binding superfamily protein | |
| rs19310064 | 8 | 19310064 | | *Glyma.08G232600* | 19216622 | | 19224146 | CTP synthase family protein | |
|  |  |  | | *Glyma.08G232800* | 19269469 | | 19273233 | AGC kinase 1.7 | |
|  |  |  | | *Glyma.08G232900* | 19300400 | | 19303730 | DNA glycosylase superfamily protein | |
|  |  |  | | *Glyma.08G233000* | 19323313 | | 19326124 | beta glucosidase 24 | |
|  |  |  | | *Glyma.08G233100* | 19335786 | | 19340584 | PIF1 helicase | |
|  |  |  | | *Glyma.08G233200* | 19351700 | | 19357626 | fatty acid reductase 5 | |
|  |  |  | | *Glyma.08G233300* | 19364232 | | 19367220 | metacaspase 5 | |
|  |  |  | | *Glyma.08G233400* | 19368724 | | 19369138 | RING/U-box superfamily protein | |
|  |  |  | | *Glyma.08G233500* | 19384636 | | 19388161 | metacaspase 4 | |
| rs31044180 | 9 | 31044180 | | *Glyma.09G125600* | 31047252 | | 31063299 | Protein of unknown function (DUF707) | |
|  |  |  | | *Glyma.09G125900* | 31084637 | | 31087925 | receptor like protein 9 | |
|  |  |  | | *Glyma.09G126100* | 31089660 | | 31091018 | Tetratricopeptide repeat (TPR)-like superfamily protein | |
|  |  |  | | *Glyma.09G126200* | 31094159 | | 31095607 | chitinase A | |
|  |  |  | | *Glyma.09G126300* | 31116116 | | 31125068 | plastidic pyruvate kinase beta subunit 1 | |
| rs49928375 | 10 | 49928375 | | *Glyma.10G275400* | 49817347 | | 49826178 | Protein kinase superfamily protein | |
|  |  |  | | *Glyma.10G275600* | 49830805 | | 49832699 | cinnamate-4-hydroxylase | |
|  |  |  | | *Glyma.10G275800* | 49838856 | | 49844704 | protein phosphatase 2A subunit A2 | |
|  |  |  | | *Glyma.10G275900* | 49849464 | | 49850499 | carboxyesterase 17 | |
|  |  |  | | *Glyma.10G276400* | 49881236 | | 49885019 | P-loop containing nucleoside triphosphate hydrolases superfamily protein | |
|  |  |  | | *Glyma.10G276600* | 49901001 | | 49903217 | Major facilitator superfamily protein | |
|  |  |  | | *Glyma.10G276700* | 49908169 | | 49910491 | Major facilitator superfamily protein | |
|  |  |  | | *Glyma.10G276800* | 49917744 | | 49919888 | Major facilitator superfamily protein | |
|  |  |  | | *Glyma.10G276900* | 49922223 | | 49930810 | kow domain-containing transcription factor 1 | |
|  |  |  | | *Glyma.10G277100* | 49932961 | | 49936949 | Protein phosphatase 2A regulatory B subunit family protein | |
|  |  |  | | *Glyma.10G277200* | 49938040 | | 49943119 | Pectin lyase-like superfamily protein | |
|  |  |  | | *Glyma.10G277300* | 49950734 | | 49951378 | ovate family protein 13 | |
|  |  |  | | *Glyma.10G277600* | 49967856 | | 49970190 | Pentatricopeptide repeat (PPR) superfamily protein | |
|  |  |  | | *Glyma.10G277800* | 50004232 | | 50006000 | Duplicated homeodomain-like superfamily protein | |
|  |  |  | | *Glyma.10G277900* | 50008091 | | 50009714 | F1F0-ATPase inhibitor protein, putative | |
|  |  |  | | *Glyma.10G278200* | 50022811 | | 50029454 | clathrin adaptor complexes medium subunit family protein | |
| rs7543892 | 10 | 7543892 | | *Glyma.10G072800* | 7468072 | | 7471098 | PIF1 helicase | |
|  |  |  | | *Glyma.10G073000* | 7486593 | | 7487396 | Ubiquitin-like superfamily protein | |
|  |  |  | | *Glyma.10G073100* | 7518531 | | 7524700 | PIF1 helicase | |
|  |  |  | | *Glyma.10G073200* | 7526300 | | 7528774 | Nucleic acid-binding, OB-fold-like protein | |
|  |  |  | | *Glyma.10G073400* | 7557716 | | 7560013 | alpha carbonic anhydrase 7 | |
|  |  |  | | *Glyma.10G073500* | 7583618 | | 7587863 | ARM repeat superfamily protein | |
|  |  |  | | *Glyma.10G073800* | 7639982 | | 7645813 | ABC2 homolog 12 | |
| rs14593163 | 17 | 14593163 | | *Glyma.17G162400* | 14412395 | | 14414851 | TTF-type zinc finger protein with HAT dimerisation domain | |
|  |  |  | | *Glyma.17G162600* | 14432028 | | 14434043 | P-loop containing nucleoside triphosphate hydrolases superfamily protein | |
|  |  |  | | *Glyma.17G162700* | 14474135 | | 14474806 | glutathione S-transferase THETA 2 | |
|  |  |  | | *Glyma.17G162800* | 14500483 | | 14502989 | GRAS family transcription factor | |
|  |  |  | | *Glyma.17G163600* | 14606633 | | 14610410 | Ribonuclease III family protein | |
|  |  |  | | *Glyma.17G163700* | 14613303 | | 14621128 | casein kinase alpha 1 | |
|  |  |  | | *Glyma.17G163800* | 14622846 | | 14625505 | Uncharacterised protein family SERF | |
|  |  |  | | *Glyma.17G164100* | 14689702 | | 14692966 | Subtilase family protein | |
| rs24979561 | 17 | 24979561 | | *Glyma.17G188700* | 24919859 | | 24928912 | hAT dimerisation domain-containing protein / transposase-related | |
| rs19962490 | 18 | 19962490 | | *Glyma.18G136400* | 19991643 | | 19993714 | TTF-type zinc finger protein with HAT dimerisation domain | |
|  |  |  | | *Glyma.18G136500* | 19997853 | | 20004268 | K+ uptake permease 6 | |
| rs588498 | 18 | 588498 | | *Glyma.18G006800* | 495989 | | 502111 | DNA-binding HORMA family protein | |
|  |  |  | | *Glyma.18G006900* | 504226 | | 508901 | SCP1-like small phosphatase 5 | |
|  |  |  | | *Glyma.18G007100* | 514114 | | 514936 | nuclear factor Y, subunit C13 | |
|  |  |  | | *Glyma.18G007500* | 563494 | | 565524 | Ribosomal protein L22p/L17e family protein | |
|  |  |  | | *Glyma.18G007700* | 568294 | | 572190 | Minichromosome maintenance (MCM2/3/5) family protein | |
|  |  |  | | *Glyma.18G007900* | 574957 | | 578056 | Galactose oxidase/kelch repeat superfamily protein | |
|  |  |  | | *Glyma.18G008400* | 607810 | | 610597 | Cysteine proteinases superfamily protein | |
|  |  |  | | *Glyma.18G008700* | 624556 | | 633668 | Protein kinase superfamily protein | |
|  |  |  | | *Glyma.18G008900* | 645064 | | 648182 | Acid phosphatase/vanadium-dependent haloperoxidase-related protein | |
|  |  |  | | *Glyma.18G009200* | 667359 | | 671598 | NAD(P)-binding Rossmann-fold superfamily protein | |
|  |  |  | | *Glyma.18G009400* | 679092 | | 681773 | lysine histidine transporter 1 | |
|  |  |  | | *Glyma.18G009500* | 682756 | | 686119 | Ankyrin repeat family protein | |
| rs37558520 | 19 | 37558520 | | *Glyma.19G118000* | 37469212 | | 37472211 | cycling DOF factor 3 | |
|  |  |  | | *Glyma.19G118100* | 37508562 | | 37509254 | Peroxidase superfamily protein | |
|  |  |  | | *Glyma.19G118400* | 37574793 | | 37577163 | WUSCHEL related homeobox 11 | |
|  |  |  | | *Glyma.19G118500* | 37592389 | | 37594845 | myb domain protein 117 | |
|  |  |  | | *Glyma.19G118800* | 37643375 | | 37647739 | frataxin homolog | |
| rs6204830 | 19 | 6204830 | | *Glyma.19G041900* | 6109157 | | 6115034 | TRICHOME BIREFRINGENCE-LIKE 19 | |
|  |  |  | | *Glyma.19G042400* | 6177407 | | 6180442 | tryptophan synthase beta type 2 | |
|  |  |  | | *Glyma.19G042500* | 6199179 | | 6202156 | Protein of unknown function (DUF1262) | |
|  |  |  | | *Glyma.19G042600* | 6211567 | | 6214311 | NB-ARC domain-containing disease resistance protein | |
|  |  |  | | *Glyma.19G042700* | 6236487 | | 6239735 | Integral membrane HRF1 family protein | |
|  |  |  | | *Glyma.19G042900* | 6251912 | | 6255738 | Methylenetetrahydrofolate reductase family protein | |
|  |  |  | | *Glyma.19G043200* | 6293349 | | 6295943 | glycosyl hydrolase family 81 protein | |
| rs34774232 | 20 | 34774232 | | *Glyma.20G104500* | 34678294 | | 34681733 | Pentatricopeptide repeat (PPR) superfamily protein | |
|  |  |  | | *Glyma.20G104600* | 34682410 | | 34684475 | nodulin MtN21 /EamA-like transporter family protein | |
|  |  |  | | *Glyma.20G104700* | 34687457 | | 34690497 | CAX interacting protein 4 | |
|  |  |  | | *Glyma.20G104900* | 34706863 | | 34713989 | Transcription factor jumonji (jmjC) domain-containing protein | |
|  |  |  | | *Glyma.20G105000* | 34726756 | | 34729686 | F-box/RNI-like superfamily protein | |
|  |  |  | | *Glyma.20G105400* | 34796301 | | 34808587 | mechanosensitive channel of small conductance-like 6 | |
|  |  |  | | *Glyma.20G105500* | 34813357 | | 34815998 | FAD/NAD(P)-binding oxidoreductase family protein | |
| rs35815938 | 20 | 35815938 | | *Glyma.20G116500* | 35894331 | | 35895981 | isopentenyltransferase 1 | |
| rs7167202 | 1 | 7167202 | *Glyma.01G054700* | | 7072113 | 7074464 | | | TTF-type zinc finger protein with HAT dimerisation domain |
|  |  |  | *Glyma.01G054800* | | 7093897 | 7097567 | | | Plant protein of unknown function (DUF863) |
|  |  |  | *Glyma.01G054900* | | 7108035 | 7110521 | | | Tetratricopeptide repeat (TPR)-like superfamily protein |
|  |  |  | *Glyma.01G055000* | | 7113117 | 7115770 | | | uvrB/uvrC motif-containing protein |
|  |  |  | *Glyma.01G055100* | | 7122525 | 7123323 | | | NB-ARC domain-containing disease resistance protein |
|  |  |  | *Glyma.01G055200* | | 7147570 | 7149067 | | | chitinase A |
|  |  |  | *Glyma.01G055300* | | 7150244 | 7159553 | | | appr-1-p processing enzyme family protein |
|  |  |  | *Glyma.01G055500* | | 7168367 | 7171683 | | | Ankyrin repeat family protein |
|  |  |  | *Glyma.01G055700* | | 7212719 | 7214922 | | | HCO3- transporter family |
|  |  |  | *Glyma.01G055800* | | 7221416 | 7222744 | | | Chaperone DnaJ-domain superfamily protein |
|  |  |  | *Glyma.01G055900* | | 7228693 | 7231351 | | | RING/U-box superfamily protein |
|  |  |  | *Glyma.01G056000* | | 7231999 | 7235244 | | | Remorin family protein |
|  |  |  | *Glyma.01G056100* | | 7235777 | 7238343 | | | senescence-related gene 1 |
|  |  |  | *Glyma.01G056200* | | 7266581 | 7268146 | | | mitochondrial editing factor 21 |
|  |  |  | *Glyma.01G056300* | | 7269225 | 7273861 | | | Leucine-rich repeat (LRR) family protein |
| rs9140707 | 7 | 9140707 | *Glyma.07G096400* | | 9042279 | 9047301 | | | Protein of unknown function (DUF789) |
|  |  |  | *Glyma.07G096500* | | 9048744 | 9054130 | | | ATP binding microtubule motor family protein |
|  |  |  | *Glyma.07G096600* | | 9057087 | 9064826 | | | phosphoglucosamine mutase-related |
|  |  |  | *Glyma.07G096700* | | 9066581 | 9067553 | | | Bifunctional inhibitor/lipid-transfer protein/seed storage 2S albumin superfamily protein |
|  |  |  | *Glyma.07G096800* | | 9079793 | 9081431 | | | chlorophyllase 2 |
|  |  |  | *Glyma.07G096900* | | 9083837 | 9085839 | | | chlorophyllase 1 |
|  |  |  | *Glyma.07G097000* | | 9118318 | 9120182 | | | chlorophyllase 1 |
|  |  |  | *Glyma.07G097100* | | 9123060 | 9125558 | | | Reticulon family protein |
|  |  |  | *Glyma.07G097200* | | 9128724 | 9128960 | | | Putative membrane lipoprotein |
|  |  |  | *Glyma.07G097300* | | 9139095 | 9141147 | | | Tetratricopeptide repeat (TPR)-like superfamily protein |
|  |  |  | *Glyma.07G097400* | | 9141472 | 9143551 | | | Ribosomal protein S24/S35, mitochondrial |
|  |  |  | *Glyma.07G097500* | | 9157923 | 9159868 | | | Reticulon family protein |
|  |  |  | *Glyma.07G097700* | | 9180670 | 9186248 | | | RING/U-box superfamily protein |
|  |  |  | *Glyma.07G097800* | | 9188050 | 9193513 | | | Uncharacterised conserved protein UCP031088, alpha/beta hydrolase |
|  |  |  | *Glyma.07G097900* | | 9199620 | 9202160 | | | Octicosapeptide/Phox/Bem1p family protein |
|  |  |  | *Glyma.07G098100* | | 9213663 | 9215834 | | | Domain of unknown function (DUF2431) |
|  |  |  | *Glyma.07G098200* | | 9230897 | 9233289 | | | Copper transport protein family |
| rs18105573 | 8 | 18105573 | *Glyma.08G221700* | | 18002978 | 18006419 | | | RNA-binding KH domain-containing protein |
|  |  |  | *Glyma.08G221800* | | 18006458 | 18012023 | | | ZIM-LIKE 2 |
|  |  |  | *Glyma.08G221900* | | 18013825 | 18020613 | | | alpha-mannosidase 1 |
|  |  |  | *Glyma.08G222000* | | 18021880 | 18025538 | | | Cation efflux family protein |
|  |  |  | *Glyma.08G222100* | | 18026866 | 18029025 | | | Ribosomal protein L30/L7 family protein |
|  |  |  | *Glyma.08G222200* | | 18038277 | 18058503 | | | autoinhibited Ca(2+)-ATPase 9 |
|  |  |  | *Glyma.08G222300* | | 18059686 | 18063561 | | | O-fucosyltransferase family protein |
|  |  |  | *Glyma.08G222400* | | 18068218 | 18073981 | | | Coatomer, beta subunit |
|  |  |  | *Glyma.08G222500* | | 18075163 | 18077665 | | | exocyst subunit exo70 family protein G1 |
|  |  |  | *Glyma.08G222600* | | 18078248 | 18079952 | | | proton gradient regulation 7 |
|  |  |  | *Glyma.08G222800* | | 18085127 | 18088964 | | | Cellulose-synthase-like C5 |
|  |  |  | *Glyma.08G222900* | | 18090096 | 18091341 | | | ATP synthase epsilon chain, mitochondrial |
|  |  |  | *Glyma.08G223000* | | 18093196 | 18102042 | | | exocyst complex component 84B |
|  |  |  | *Glyma.08G223200* | | 18103948 | 18105613 | | | RNA-binding (RRM/RBD/RNP motifs) family protein |
|  |  |  | *Glyma.08G223300* | | 18107199 | 18109906 | | | D-3-phosphoglycerate dehydrogenase |
|  |  |  | *Glyma.08G223400* | | 18127350 | 18129276 | | | mitogen-activated protein kinase kinase 4 |
|  |  |  | *Glyma.08G223600* | | 18138052 | 18142927 | | | Galactose mutarotase-like superfamily protein |
|  |  |  | *Glyma.08G223700* | | 18147188 | 18154565 | | | Protein kinase superfamily protein |
|  |  |  | *Glyma.08G223800* | | 18162779 | 18168705 | | | Rad23 UV excision repair protein family |
|  |  |  | *Glyma.08G223900* | | 18169513 | 18171372 | | | YGGT family protein |
|  |  |  | *Glyma.08G224000* | | 18175289 | 18182199 | | | Ubiquitin-specific protease family C19-related protein |
|  |  |  | *Glyma.08G224200* | | 18187905 | 18191895 | | | Ergosterol biosynthesis ERG4/ERG24 family |
|  |  |  | *Glyma.08G224400* | | 18196846 | 18202474 | | | vacuolar ATP synthase subunit A |
|  |  |  | *Glyma.08G224500* | | 18204060 | 18208035 | | | regulatory particle triple-A ATPase 6A |
|  |  |  | *Glyma.08G224600* | | 18208948 | 18211498 | | | Plastid-lipid associated protein PAP / fibrillin family protein |
| rs2669053 | 9 | 2669053 | *Glyma.09G031100* | | 2556358 | 2563473 | | | DEAD box RNA helicase family protein |
|  |  |  | *Glyma.09G031200* | | 2566174 | 2573725 | | | evolutionarily conserved C-terminal region 7 |
|  |  |  | *Glyma.09G031300* | | 2568171 | 2569388 | | | Ubiquitin carboxyl-terminal hydrolase family protein |
|  |  |  | *Glyma.09G031400* | | 2581494 | 2586811 | | | HCO3- transporter family |
|  |  |  | *Glyma.09G031600* | | 2592440 | 2595781 | | | Pectin lyase-like superfamily protein |
|  |  |  | *Glyma.09G031700* | | 2609839 | 2612385 | | | Protein phosphatase 2C family protein |
|  |  |  | *Glyma.09G031800* | | 2618477 | 2623018 | | | ribosomal protein S1 |
|  |  |  | *Glyma.09G031900* | | 2623854 | 2634294 | | | zinc knuckle (CCHC-type) family protein |
|  |  |  | *Glyma.09G032000* | | 2638311 | 2644265 | | | ubiquitin protein ligase 6 |
|  |  |  | *Glyma.09G032100* | | 2673509 | 2675937 | | | myb domain protein 78 |
|  |  |  | *Glyma.09G032200* | | 2708170 | 2715984 | | | gibberellin 2-oxidase 4 |
|  |  |  | *Glyma.09G032500* | | 2751892 | 2753408 | | | agenet domain-containing protein |
|  |  |  | *Glyma.09G032600* | | 2755503 | 2757441 | | | F-box family protein |
|  |  |  | *Glyma.09G032800* | | 2762138 | 2767670 | | | Chaperone DnaJ-domain superfamily protein |
| rs40595691 | 10 | 40595691 | *Glyma.10G171400* | | 40494363 | 40498737 | | | Integrase-type DNA-binding superfamily protein |
|  |  |  | *Glyma.10G171500* | | 40502502 | 40517656 | | | RNA binding (RRM/RBD/RNP motifs) family protein |
|  |  |  | *Glyma.10G171600* | | 40519258 | 40523745 | | | RAB GTPase homolog A5A |
|  |  |  | *Glyma.10G171700* | | 40523949 | 40538605 | | | Endonuclease/exonuclease/phosphatase family protein |
|  |  |  | *Glyma.10G171800* | | 40542750 | 40545783 | | | Heavy metal transport/detoxification superfamily protein |
|  |  |  | *Glyma.10G171900* | | 40552680 | 40562442 | | | Transcription factor jumonji (jmj) family protein / zinc finger (C5HC2 type) family protein |
|  |  |  | *Glyma.10G172000* | | 40563768 | 40568736 | | | Nucleoporin autopeptidase |
|  |  |  | *Glyma.10G172100* | | 40573252 | 40577616 | | | Polyketide cyclase/dehydrase and lipid transport superfamily protein |
|  |  |  | *Glyma.10G172200* | | 40581056 | 40586186 | | | UDP-Glycosyltransferase superfamily protein |
|  |  |  | *Glyma.10G172300* | | 40588121 | 40591913 | | | Glycosyl hydrolase superfamily protein |
|  |  |  | *Glyma.10G172400* | | 40592783 | 40603954 | | | ubiquitin-specific protease 9 |
|  |  |  | *Glyma.10G172500* | | 40614446 | 40624015 | | | RING/FYVE/PHD zinc finger superfamily protein |
|  |  |  | *Glyma.10G172600* | | 40628401 | 40633140 | | | alpha/beta-Hydrolases superfamily protein |
|  |  |  | *Glyma.10G172700* | | 40646539 | 40649573 | | | methionine gamma-lyase |
|  |  |  | *Glyma.10G172800* | | 40658709 | 40668066 | | | Tetratricopeptide repeat (TPR)-like superfamily protein |
|  |  |  | *Glyma.10G172900* | | 40666359 | 40670242 | | | YELLOW STRIPE like 1 |
|  |  |  | *Glyma.10G173000* | | 40686435 | 40696440 | | | Protein kinase superfamily protein |
|  |  |  | *Glyma.10G173100* | | 40699391 | 40701312 | | | early nodulin-like protein 8 |
| rs43000771 | 15 | 43000771 | *Glyma.15G229000* | | 42907304 | 42908462 | | | Duplicated homeodomain-like superfamily protein |
|  |  |  | *Glyma.15G229100* | | 42976994 | 42977913 | | | sulfotransferase 2A |
|  |  |  | *Glyma.15G229200* | | 42980067 | 42983136 | | | CCT motif family protein |
|  |  |  | *Glyma.15G229300* | | 42991865 | 42992644 | | | Protein of unknown function, DUF617 |
|  |  |  | *Glyma.15G229500* | | 43045904 | 43050324 | | | Plant protein of unknown function (DUF869) |
|  |  |  | *Glyma.15G229600* | | 43046109 | 43063832 | | | Tetratricopeptide repeat (TPR)-like superfamily protein |
|  |  |  | *Glyma.15G229700* | | 43053298 | 43055121 | | | Nucleotide-diphospho-sugar transferase family protein |
|  |  |  | *Glyma.15G229800* | | 43054739 | 43055522 | | | Tim10/DDP family zinc finger protein |
|  |  |  | *Glyma.15G229900* | | 43055321 | 43056426 | | | stress-inducible protein, putative |
|  |  |  | *Glyma.15G230000* | | 43064967 | 43065299 | | | helicase in vascular tissue and tapetum |
| rs5779917 | 19 | 5779917 | *Glyma.19G039600* | | 5681475 | 5682707 | | | HXXXD-type acyl-transferase family protein |
|  |  |  | *Glyma.19G039700* | | 5720079 | 5721875 | | | HXXXD-type acyl-transferase family protein |
|  |  |  | *Glyma.19G039800* | | 5736959 | 5737278 | | | GATA type zinc finger transcription factor family protein |
|  |  |  | *Glyma.19G040000* | | 5770969 | 5772171 | | | Late embryogenesis abundant protein (LEA) family protein |
|  |  |  | *Glyma.19G040100* | | 5773912 | 5781906 | | | Ypt/Rab-GAP domain of gyp1p superfamily protein |
|  |  |  | *Glyma.19G040200* | | 5791073 | 5796786 | | | cell division protein ftsH, putative |
|  |  |  | *Glyma.19G040300* | | 5819045 | 5819405 | | | Homeodomain-like superfamily protein |
|  |  |  | *Glyma.19G040400* | | 5826182 | 5829696 | | | CBL-interacting protein kinase 8 |
|  |  |  | *Glyma.19G040500* | | 5831176 | 5834955 | | | histone acetyltransferase of the CBP family 12 |
|  |  |  | *Glyma.19G040600* | | 5840532 | 5843788 | | | histone acetyltransferase of the CBP family 1 |
|  |  |  | *Glyma.19G040700* | | 5843994 | 5848776 | | | histone acetyltransferase of the CBP family 12 |
|  |  |  | *Glyma.19G040800* | | 5871283 | 5882245 | | | Proline-rich spliceosome-associated (PSP) family protein / zinc knuckle (CCHC-type) family protein |
| rs46814888 | 20 | 46814888 | *Glyma.20G234600* | | 46716599 | 46733339 | | | homolog of yeast sucrose nonfermenting 4 |
|  |  |  | *Glyma.20G234800* | | 46744367 | 46747550 | | | cyclin H;1 |
|  |  |  | *Glyma.20G234900* | | 46751287 | 46755695 | | | nuclear factor Y, subunit B4 |
|  |  |  | *Glyma.20G235100* | | 46782014 | 46785981 | | | indeterminate(ID)-domain 2 |
|  |  |  | *Glyma.20G235200* | | 46788735 | 46789537 | | | Photosystem II reaction center PsbP family protein |
|  |  |  | *Glyma.20G235300* | | 46790436 | 46798140 | | | Zinc finger, RING-type;Transcription factor jumonji/aspartyl beta-hydroxylase |
|  |  |  | *Glyma.20G235400* | | 46801199 | 46803834 | | | P-loop containing nucleoside triphosphate hydrolases superfamily protein |
|  |  |  | *Glyma.20G235500* | | 46807219 | 46815704 | | | acyl-CoA binding protein 4 |
|  |  |  | *Glyma.20G235700* | | 46819317 | 46823815 | | | Tudor/PWWP/MBT superfamily protein |
|  |  |  | *Glyma.20G235800* | | 46825099 | 46828197 | | | Transducin/WD40 repeat-like superfamily protein |
|  |  |  | *Glyma.20G236000* | | 46832583 | 46839603 | | | RECQ helicase SIM |
|  |  |  | *Glyma.20G236100* | | 46840729 | 46844649 | | | Actin-binding FH2 (formin homology 2) family protein |
|  |  |  | *Glyma.20G236200* | | 46855413 | 46862143 | | | respiratory burst oxidase homolog B |
|  |  |  | *Glyma.20G236400* | | 46872743 | 46878156 | | | rapid alkalinization factor 1 |
|  |  |  | *Glyma.20G236500* | | 46879505 | 46883572 | | | regulatory particle triple-A ATPase 5A |
|  |  |  | *Glyma.20G236600* | | 46883985 | 46884780 | | | translationally controlled tumor protein |
|  |  |  | *Glyma.20G236700* | | 46886326 | 46894385 | | | RING/U-box superfamily protein |
|  |  |  | *Glyma.20G236800* | | 46896125 | 46902283 | | | SERINE-ARGININE PROTEIN 30 |
|  |  |  | *Glyma.20G236900* | | 46905295 | 46907950 | | | Hypoxia-responsive family protein |
|  |  |  | *Glyma.20G237000* | | 46909760 | 46911144 | | | Ribosomal L22e protein family |

| **Table S12** Candidate genes in the flanking regions of peak SNPs of QEIs. | | | | | | | |
| --- | --- | --- | --- | --- | --- | --- | --- |
| Peak SNP | Chr. | Physical position (bp) | Gene ID | Gene ID | Start Position | Stop Position | Functional annotation |
| rs38321290 | 1 | 38321290 | *Glyma.01G112600* | KRH75828 | 38353594 | 38358163 | Laccase/Diphenol oxidase family protein |
|  |  |  | *Glyma.01G112700* | KRH75829 | 38401762 | 38404642 | Duplicated homeodomain-like superfamily protein |
| rs37739787 | 2 | 37739787 | *Glyma.02G197800* | KRH75830 | 37686736 | 37687986 | NDR1/HIN1-like 2 |
|  |  |  | *Glyma.02G198000* | KRH75831 | 37806790 | 37811619 | Double Clp-N motif-containing P-loop nucleoside triphosphate hydrolases superfamily protein |
| rs2799329 | 6 | 2799329 | *Glyma.06G034700* | KRH75832 | 2694644 | 2695761 | calmodulin-like 41 |
|  |  |  | *Glyma.06G035000* | KRH75833 | 2713959 | 2719986 | Protein kinase superfamily protein |
|  |  |  | *Glyma.06G035200* | KRH75834 | 2724860 | 2727223 | cytochrome P450, family 82, subfamily C, polypeptide 4 |
|  |  |  | *Glyma.06G035300* | KRH75835 | 2729440 | 2733576 | cytochrome P450, family 82, subfamily C, polypeptide 4 |
|  |  |  | *Glyma.06G035400* | KRH75836 | 2736074 | 2739766 | cytochrome P450, family 82, subfamily C, polypeptide 4 |
|  |  |  | *Glyma.06G035600* | KRH75837 | 2741102 | 2743007 | cytochrome P450, family 82, subfamily C, polypeptide 4 |
|  |  |  | *Glyma.06G035700* | KRH75838 | 2745480 | 2748590 | Homeodomain-like superfamily protein |
|  |  |  | *Glyma.06G035900* | KRH75839 | 2759124 | 2760027 | cytochrome b6f complex subunit (petM), putative |
|  |  |  | *Glyma.06G036000* | KRH75840 | 2763988 | 2765469 | Leucine-rich repeat (LRR) family protein |
|  |  |  | *Glyma.06G036100* | KRH75841 | 2772753 | 2775133 | Peptidase S24/S26A/S26B/S26C family protein |
|  |  |  | *Glyma.06G036200* | KRH75842 | 2776996 | 2780971 | nitrate transporter 1:2 |
|  |  |  | *Glyma.06G036300* | KRH75843 | 2787223 | 2790348 | Serine protease inhibitor (SERPIN) family protein |
|  |  |  | *Glyma.06G036400* | KRH75844 | 2793752 | 2800249 | mitogen-activated protein kinase kinase kinase 5 |
|  |  |  | *Glyma.06G036500* | KRH75845 | 2800883 | 2810069 | Spc97 / Spc98 family of spindle pole body (SBP) component |
|  |  |  | *Glyma.06G036700* | KRH75846 | 2815452 | 2816744 | Cupredoxin superfamily protein |
|  |  |  | *Glyma.06G036800* | KRH75847 | 2820598 | 2822127 | myb domain protein r1 |
|  |  |  | *Glyma.06G036900* | KRH75848 | 2833967 | 2834662 | Protein of unknown function (DUF617) |
|  |  |  | *Glyma.06G037000* | KRH75849 | 2839896 | 2841514 | Protein of unknown function (DUF1645) |
|  |  |  | *Glyma.06G037300* | KRH75850 | 2861369 | 2870869 | uroporphyrinogen-III synthase family protein |
|  |  |  | *Glyma.06G037600* | KRH75851 | 2879848 | 2887029 | RNA-binding protein 47C |
|  |  |  | *Glyma.06G037800* | KRH75852 | 2894530 | 2896325 | P-loop containing nucleoside triphosphate hydrolases superfamily protein |
| rs50270401 | 10 | 50270401 | *Glyma.10G280500* | KRH75853 | 50163026 | 50173048 | GTP binding |
|  |  |  | *Glyma.10G280600* | KRH75854 | 50174326 | 50178862 | Nucleotide-diphospho-sugar transferase family protein |
|  |  |  | *Glyma.10G280700* | KRH75855 | 50181762 | 50199555 | Sec23/Sec24 protein transport family protein |
|  |  |  | *Glyma.10G280800* | KRH75856 | 50201731 | 50204631 | Uncharacterised protein family (UPF0041) |
|  |  |  | *Glyma.10G281000* | KRH75857 | 50214835 | 50221594 | Homeodomain-like superfamily protein |
|  |  |  | *Glyma.10G281100* | KRH75858 | 50240906 | 50243956 | AP2/B3-like transcriptional factor family protein |
|  |  |  | *Glyma.10G281200* | KRH75859 | 50246999 | 50249746 | AP2/B3-like transcriptional factor family protein |
|  |  |  | *Glyma.10G281300* | KRH75860 | 50251157 | 50252837 | AP2/B3-like transcriptional factor family protein |
|  |  |  | *Glyma.10G281400* | KRH75861 | 50254563 | 50258108 | hydroxypyruvate reductase |
|  |  |  | *Glyma.10G281500* | KRH75862 | 50260657 | 50263445 | GCIP-interacting family protein |
|  |  |  | *Glyma.10G281600* | KRH75863 | 50265922 | 50270455 | Arabidopsis phospholipase-like protein (PEARLI 4) family |
|  |  |  | *Glyma.10G281800* | KRH75864 | 50284138 | 50287875 | basic helix-loop-helix (bHLH) DNA-binding superfamily protein |
|  |  |  | *Glyma.10G281900* | KRH75865 | 50290469 | 50291914 | Cupredoxin superfamily protein |
|  |  |  | *Glyma.10G282000* | KRH75866 | 50294239 | 50301335 | ubiquitin-conjugating enzyme 23 |
|  |  |  | *Glyma.10G282100* | KRH75867 | 50303921 | 50307325 | FAD/NAD(P)-binding oxidoreductase family protein |
|  |  |  | *Glyma.10G282200* | KRH75868 | 50314887 | 50318030 | FAD/NAD(P)-binding oxidoreductase family protein |
|  |  |  | *Glyma.10G282300* | KRH75869 | 50320024 | 50322909 | FAD/NAD(P)-binding oxidoreductase family protein |
|  |  |  | *Glyma.10G282400* | KRH75870 | 50323152 | 50331936 | Splicing factor, CC1-like |
|  |  |  | *Glyma.10G282700* | KRH75871 | 50332764 | 50339544 | translation initiation factor 3 (IF-3) family protein |
|  |  |  | *Glyma.10G282900* | KRH75872 | 50358644 | 50363494 | Protein phosphatase 2C family protein |
|  |  |  | *Glyma.10G283000* | KRH75873 | 50369012 | 50373942 | Protein of unknown function (DUF630 and DUF632) |
|  |  |  | *Glyma.10G283100* | KRH75874 | 50376354 | 50379746 | Protein of unknown function (DUF3353) |
| rs29527738 | 11 | 29527738 | *Glyma.11G207200* | KRH75875 | 29447370 | 29448199 | serine carboxypeptidase-like 45 |
|  |  |  | *Glyma.11G207300* | KRH75876 | 29452563 | 29461608 | cysteine-rich RLK (RECEPTOR-like protein kinase) 2 |
|  |  |  | *Glyma.11G207400* | KRH75877 | 29487026 | 29487550 | TTF-type zinc finger protein with HAT dimerisation domain |
|  |  |  | *Glyma.11G207500* | KRH75878 | 29490841 | 29496018 | cysteine-rich RLK (RECEPTOR-like protein kinase) 2 |
|  |  |  | *Glyma.11G207600* | KRH75879 | 29503252 | 29504278 | serine carboxypeptidase-like 48 |
| rs9782629 | 14 | 9782629 | *Glyma.14G099700* | KRH75880 | 9714389 | 9719658 | Alfin-like 5 |
|  |  |  | *Glyma.14G099800* | KRH75881 | 9727169 | 9729817 | Mitochondrial substrate carrier family protein |
|  |  |  | *Glyma.14G099900* | KRH75882 | 9736779 | 9737754 | 17.6 kDa class II heat shock protein |
|  |  |  | *Glyma.14G100000* | KRH75883 | 9740301 | 9741067 | 17.6 kDa class II heat shock protein |
|  |  |  | *Glyma.14G100100* | KRH75884 | 9770124 | 9770678 | WRKY DNA-binding protein 50 |
|  |  |  | *Glyma.14G100300* | KRH75885 | 9818448 | 9819839 | Concanavalin A-like lectin protein kinase family protein |
|  |  |  | *Glyma.14G100400* | KRH75886 | 9839656 | 9841977 | Concanavalin A-like lectin protein kinase family protein |
|  |  |  | *Glyma.14G100500* | KRH75887 | 9859595 | 9860951 | cyclic nucleotide gated channel 3 |
|  |  |  | *Glyma.14G100600* | KRH75888 | 9884437 | 9887016 | Concanavalin A-like lectin protein kinase family protein |
|  |  |  | *Glyma.14G100700* | KRH75889 | 9887705 | 9890065 | Concanavalin A-like lectin protein kinase family protein |
| rs19530677 | 16 | 19530677 | *Glyma.16G099800* | KRH75890 | 19456420 | 19474307 | alpha/beta-Hydrolases superfamily protein |
|  |  |  | *Glyma.16G100000* | KRH75891 | 19639139 | 19640322 | Protein kinase superfamily protein |
|  |  |  | *Glyma.16G205700* | KRH75892 | 36636652 | 36645502 | ATP-dependent Clp protease |
|  |  |  | *Glyma.16G205900* | KRH75893 | 36650239 | 36655749 | DNAJ heat shock N-terminal domain-containing protein |
|  |  |  | *Glyma.16G206000* | KRH75894 | 36656248 | 36658633 | Pentatricopeptide repeat (PPR) superfamily protein |
|  |  |  | *Glyma.16G206200* | KRH75895 | 36663932 | 36664834 | HSP20-like chaperones superfamily protein |
|  |  |  | *Glyma.16G206400* | KRH75896 | 36670972 | 36674446 | plastid movement impaired1 |
|  |  |  | *Glyma.16G206500* | KRH75897 | 36675871 | 36679332 | Ribosomal protein L2 family |
|  |  |  | *Glyma.16G206600* | KRH75898 | 36680202 | 36681939 | ATP binding;nucleic acid binding;helicases |
|  |  |  | *Glyma.16G206800* | KRH75899 | 36684395 | 36684947 | A20/AN1-like zinc finger family protein |
|  |  |  | *Glyma.16G206900* | KRH75900 | 36686943 | 36688802 | ENTH/ANTH/VHS superfamily protein |
|  |  |  | *Glyma.16G207000* | KRH75901 | 36692744 | 36698385 | Class I peptide chain release factor |
|  |  |  | *Glyma.16G207100* | KRH75902 | 36701476 | 36704764 | oxidoreductases |
|  |  |  | *Glyma.16G207200* | KRH75903 | 36705861 | 36716997 | histone-lysine N-methyltransferase ASHH3 |
|  |  |  | *Glyma.16G207300* | KRH75904 | 36718287 | 36720700 | Ribosomal protein S3 family protein |
|  |  |  | *Glyma.16G207400* | KRH75905 | 36722555 | 36724883 | Ribosomal protein S3 family protein |
|  |  |  | *Glyma.16G207500* | KRH75906 | 36728513 | 36732189 | Peroxidase superfamily protein |
|  |  |  | *Glyma.16G207600* | KRH75907 | 36733685 | 36738992 | Got1/Sft2-like vescicle transport protein family |
|  |  |  | *Glyma.16G207700* | KRH75908 | 36739878 | 36743378 | catalytic LigB subunit of aromatic ring-opening dioxygenase family |
|  |  |  | *Glyma.16G207800* | KRH75909 | 36750028 | 36751833 | catalytic LigB subunit of aromatic ring-opening dioxygenase family |
|  |  |  | *Glyma.16G208100* | KRH75910 | 36776806 | 36778169 | carboxyesterase 18 |
|  |  |  | *Glyma.16G208200* | KRH75911 | 36780247 | 36781946 | carboxyesterase 18 |
|  |  |  | *Glyma.16G208300* | KRH75912 | 36786114 | 36787686 | carboxyesterase 18 |
|  |  |  | *Glyma.16G208400* | KRH75913 | 36789890 | 36801967 | chloride channel C |
|  |  |  | *Glyma.16G208500* | KRH75914 | 36806718 | 36809299 | Translation initiation factor IF6 |
|  |  |  | *Glyma.16G208600* | KRH75915 | 36810473 | 36813869 | Translation initiation factor IF6 |
|  |  |  | *Glyma.16G208700* | KRH75916 | 36814752 | 36820289 | TCP-1/cpn60 chaperonin family protein |
|  |  |  | *Glyma.16G208800* | KRH75917 | 36826359 | 36834460 | SWIB/MDM2 domain;Plus-3;GYF |
|  |  |  | *Glyma.16G209000* | KRH75918 | 36841786 | 36845498 | Disease resistance protein (TIR-NBS-LRR class) family |
|  |  |  | *Glyma.16G209100* | KRH75919 | 36847918 | 36850157 | Plant protein of unknown function (DUF247) |
| rs49948296 | 18 | 49948296 | *Glyma.18G212000* | KRH75920 | 49844858 | 49845229 | Phototropic-responsive NPH3 family protein |
|  |  |  | *Glyma.18G212100* | KRH75921 | 49846552 | 49850610 | saposin B domain-containing protein |
|  |  |  | *Glyma.18G212200* | KRH75922 | 49872222 | 49875889 | SOS3-interacting protein 1 |
|  |  |  | *Glyma.18G212300* | KRH75923 | 49880681 | 49885779 | TATA binding protein 2 |
|  |  |  | *Glyma.18G212500* | KRH75924 | 49917527 | 49919691 | AZA-guanine resistant1 |
|  |  |  | *Glyma.18G212600* | KRH75925 | 49924801 | 49930944 | N-acetylglucosaminyl transferase component family protein / Gpi1 family protein |
|  |  |  | *Glyma.18G212700* | KRH75926 | 49942123 | 49944169 | SOS3-interacting protein 4 |
|  |  |  | *Glyma.18G212800* | KRH75927 | 49960776 | 49964807 | serine/threonine protein kinase 2 |
|  |  |  | *Glyma.18G213100* | KRH75928 | 49992860 | 50002425 | aminophospholipid ATPase 1 |
|  |  |  | *Glyma.18G213200* | KRH75929 | 50013378 | 50015386 | WRKY DNA-binding protein 70 |
|  |  |  | *Glyma.18G213300* | KRH75930 | 50026808 | 50028261 | Protein of unknown function, DUF599 |
|  |  |  | *Glyma.18G213400* | KRH75931 | 50037003 | 50043647 | vacuolar sorting receptor homolog 1 |
|  |  |  | *Glyma.18G213500* | KRH75932 | 50044567 | 50050213 | Transmembrane proteins 14C |
| rs55080151 | 18 | 55080151 | *Glyma.18G264800* | KRH75933 | 54983775 | 54984648 | LYR family of Fe/S cluster biogenesis protein |
|  |  |  | *Glyma.18G264900* | KRH75934 | 54986680 | 54988748 | calmodulin-binding protein-related |
|  |  |  | *Glyma.18G265000* | KRH75935 | 54990671 | 54993790 | putative endonuclease or glycosyl hydrolase with C2H2-type zinc finger domain |
|  |  |  | *Glyma.18G265100* | KRH75936 | 54995065 | 54997875 | UDP-glucose 6-dehydrogenase family protein |
|  |  |  | *Glyma.18G265200* | KRH75937 | 55004174 | 55008145 | TPX2 (targeting protein for Xklp2) protein family |
|  |  |  | *Glyma.18G265300* | KRH75938 | 55010646 | 55014571 | biotin carboxyl carrier protein 2 |
|  |  |  | *Glyma.18G265400* | KRH75939 | 55017312 | 55018922 | expansin A23 |
|  |  |  | *Glyma.18G265500* | KRH75940 | 55019514 | 55022070 | F-box family protein |
|  |  |  | *Glyma.18G265600* | KRH75941 | 55024122 | 55024816 | Cytochrome P450 superfamily protein |
|  |  |  | *Glyma.18G265700* | KRH75942 | 55026144 | 55028979 | UDP-Glycosyltransferase superfamily protein |
|  |  |  | *Glyma.18G265800* | KRH75943 | 55035928 | 55038615 | UDP-Glycosyltransferase superfamily protein |
|  |  |  | *Glyma.18G265900* | KRH75944 | 55040041 | 55042737 | UDP-Glycosyltransferase superfamily protein |
|  |  |  | *Glyma.18G266000* | KRH75945 | 55045033 | 55047026 | UDP-Glycosyltransferase superfamily protein |
|  |  |  | *Glyma.18G266100* | KRH75946 | 55052591 | 55056489 | UDP-Glycosyltransferase superfamily protein |
|  |  |  | *Glyma.18G266200* | KRH75947 | 55065716 | 55066843 | Histone superfamily protein |
|  |  |  | *Glyma.18G266300* | KRH75948 | 55071421 | 55074175 | dsRNA-binding domain-like superfamily protein |
|  |  |  | *Glyma.18G266400* | KRH75949 | 55076548 | 55086297 | RS2-interacting KH protein |
|  |  |  | *Glyma.18G266500* | KRH75950 | 55088284 | 55091427 | glycine-rich RNA-binding protein 3 |
|  |  |  | *Glyma.18G266600* | KRH75951 | 55096167 | 55099111 | exocyst subunit exo70 family protein E1 |
|  |  |  | *Glyma.18G266700* | KRH75952 | 55105365 | 55112399 | serine carboxypeptidase-like 25 |
|  |  |  | *Glyma.18G266900* | KRH75953 | 55125943 | 55128833 | glycosyl hydrolase family 81 protein |
|  |  |  | *Glyma.18G267000* | KRH75954 | 55133437 | 55137590 | receptor-like protein kinase 2 |
|  |  |  | *Glyma.18G267100* | KRH75955 | 55151772 | 55154403 | glycosyl hydrolase family 81 protein |
|  |  |  | *Glyma.18G267200* | KRH75956 | 55161119 | 55166101 | ABI five binding protein 3 |
|  |  |  | *Glyma.18G267200* | KRH75957 | 55161119 | 55166101 | ABI five binding protein 3 |
|  |  |  | *Glyma.18G267400* | KRH75958 | 55171420 | 55176356 | Nuclear transport factor 2 (NTF2) family protein with RNA binding (RRM-RBD-RNP motifs) domain |
|  |  |  | *Glyma.18G267500* | KRH75959 | 55179516 | 55181798 | O-methyltransferase family protein |
|  |  |  | *Glyma.18G267600* | KRH75960 | 55186346 | 55189874 | 3\'-5\'-exoribonuclease family protein |
|  |  |  | *Glyma.18G267700* | KRH75961 | 55189880 | 55191241 | 3\'-5\'-exoribonuclease family protein |

| **Table S13** Gene-based association analysis of candidate genes. | | | | | | | | | |
| --- | --- | --- | --- | --- | --- | --- | --- | --- | --- |
| Group | Gene ID | Chr. | Physical position (bp) | Trait | Allele | Location | Region | -log_10_^(P)^ | Functional annotation |
| group-Ⅰ | *Glyma.03G186200* | 3 | 39794841 | Total-Toc | A/G | Harbin | exonic | 1.75 | RAB GTPase homolog C2A |
|  |  |  |  |  |  | Liaoning |  | 3.30 |  |
|  |  |  |  |  |  | Jilin |  | 2.85 |  |
|  |  |  | 39796103 | Total-Toc | G/T | Harbin | downstream | 1.55 |  |
|  |  |  |  |  |  | Liaoning |  | 2.50 |  |
|  |  |  |  |  |  | Jilin |  | 1.70 |  |
| group-Ⅰ | *Glyma.03G186500* | 3 | 39818854 | Total-Toc | T/G | Harbin | exonic | 2.12 | transducin family protein / WD-40 repeat family protein |
|  |  |  |  |  |  | Liaoning |  | 2.93 |  |
|  |  |  |  |  |  | Jilin |  | 2.50 |  |
|  |  |  | 39818869 | Total-Toc | C/T | Harbin | intronic | 2.12 |  |
|  |  |  |  |  |  | Liaoning |  | 2.93 |  |
|  |  |  |  |  |  | Jilin |  | 2.50 |  |
|  |  |  | 39819771 | Total-Toc | A/C | Harbin | exonic | 1.54 |  |
|  |  |  |  |  |  | Liaoning |  | 2.33 |  |
|  |  |  |  |  |  | Jilin |  | 1.89 |  |
|  |  |  | 39819930 | Total-Toc | A/C | Harbin | exonic | 1.55 |  |
|  |  |  |  |  |  | Liaoning |  | 2.27 |  |
|  |  |  |  |  |  | Jilin |  | 1.84 |  |
|  |  |  | 39821130 | Total-Toc | C/T | Harbin | exonic | 2.12 |  |
|  |  |  |  |  |  | Liaoning |  | 2.93 |  |
|  |  |  |  |  |  | Jilin |  | 2.50 |  |
|  |  |  | 39823409 | Total-Toc | T/C | Harbin | intronic | 2.12 |  |
|  |  |  |  |  |  | Liaoning |  | 2.93 |  |
|  |  |  |  |  |  | Jilin |  | 2.50 |  |
| group-Ⅰ | *Glyma.06G038000* | 6 | 2908711 | δ-Toc | C/T | Harbin | upstream | 7.72 | Alpha/beta-Hydrolases superfamily protein |
|  |  |  |  |  |  | Liaoning |  | 6.41 |  |
|  |  |  |  |  |  | Jilin |  | 5.72 |  |
|  |  |  | 45983026 | δ-Toc | G/T | Harbin | downstream | 8.87 |  |
|  |  |  |  |  |  | Liaoning |  | 7.49 |  |
|  |  |  |  |  |  | Jilin |  | 6.67 |  |
| group-Ⅰ | *Glyma.17G188700* | 17 | 24919348 | α-Toc | C/T | Harbin | upstream | 2.23 | hAT dimerisation domain-containing protein / transposase-related |
|  |  |  |  |  |  | Liaoning |  | 2.54 |  |
|  |  |  |  |  |  | Jilin |  | 2.47 |  |
|  |  |  | 24919381 | α-Toc | C/T | Harbin | upstream | 2.23 |  |
|  |  |  |  |  |  | Liaoning |  | 2.54 |  |
|  |  |  |  |  |  | Jilin |  | 2.47 |  |
|  |  |  | 24919416 | α-Toc | C/T | Harbin | upstream | 2.23 |  |
|  |  |  |  |  |  | Liaoning |  | 2.54 |  |
|  |  |  |  |  |  | Jilin |  | 2.47 |  |
|  |  |  | 24920365 | α-Toc | G/A | Harbin | exonic | 5.85 |  |
|  |  |  |  |  |  | Liaoning |  | 8.55 |  |
|  |  |  |  |  |  | Jilin |  | 9.15 |  |
|  |  |  | 24922656 | α-Toc | A/G | Harbin | exonic | 2.26 |  |
|  |  |  |  |  |  | Liaoning |  | 2.00 |  |
|  |  |  |  |  |  | Jilin |  | 1.52 |  |
|  |  |  | 24922901 | α-Toc | T/C | Harbin | exonic | 2.21 |  |
|  |  |  |  |  |  | Liaoning |  | 2.47 |  |
|  |  |  |  |  |  | Jilin |  | 1.67 |  |
| group-Ⅱ | *Glyma.01G054800* | 1 | 7094520 | γ-Toc | C/T | Harbin | exonic | 2.13 | Plant protein of unknown function (DUF863) |
|  |  |  |  |  |  | Liaoning |  | 2.54 |  |
|  |  |  |  |  |  | Jilin |  | 2.72 |  |
|  |  |  | 7094818 | γ-Toc | T/C | Harbin | intronic | 2.20 |  |
|  |  |  |  |  |  | Liaoning |  | 2.81 |  |
|  |  |  |  |  |  | Jilin |  | 2.05 |  |
|  |  |  | 7094520 | Total-Toc | C/T | Harbin | exonic | 2.33 |  |
|  |  |  |  |  |  | Liaoning |  | 2.43 |  |
|  |  |  |  |  |  | Jilin |  | 2.93 |  |
|  |  |  | 7094818 | Total-Toc | T/C | Harbin | intronic | 2.42 |  |
|  |  |  |  |  |  | Liaoning |  | 2.20 |  |
|  |  |  |  |  |  | Jilin |  | 2.77 |  |
| group-Ⅱ | *Glyma.08G222300* | 8 | 18061449 | δ-Toc | T/A | Harbin | exonic | 2.81 | O-fucosyltransferase family protein |
|  |  |  |  |  |  | Liaoning |  | 2.84 |  |
|  |  |  |  |  |  | Jilin |  | 1.84 |  |
|  |  |  | 18061942 | δ-Toc | T/A | Harbin | exonic | 3.05 |  |
|  |  |  |  |  |  | Liaoning |  | 3.11 |  |
|  |  |  |  |  |  | Jilin |  | 2.05 |  |
|  |  |  | 18062024 | δ-Toc | A/T | Harbin | exonic | 1.11 |  |
|  |  |  |  |  |  | Liaoning |  | 2.60 |  |
|  |  |  |  |  |  | Jilin |  | 0.68 |  |
|  |  |  | 18062224 | δ-Toc | T/C | Harbin | intronic | 1.11 |  |
|  |  |  |  |  |  | Liaoning |  | 2.60 |  |
|  |  |  |  |  |  | Jilin |  | 0.68 |  |
|  |  |  | 18063365 | δ-Toc | A/C | Harbin | exonic | 2.33 |  |
|  |  |  |  |  |  | Liaoning |  | 2.38 |  |
|  |  |  |  |  |  | Jilin |  | 1.44 |  |
| group-Ⅱ | *Glyma.09G032100* | 9 | 2674177 | Total-Toc | T/C | Harbin | exonic | 2.26 | myb domain protein 78 |
|  |  |  |  |  |  | Liaoning |  | 3.87 |  |
|  |  |  |  |  |  | Jilin |  | 4.27 |  |
|  |  |  | 2674189 | Total-Toc | T/A | Harbin | intronic | 2.26 |  |
|  |  |  |  |  |  | Liaoning |  | 3.87 |  |
|  |  |  |  |  |  | Jilin |  | 4.27 |  |
|  |  |  | 2674397 | Total-Toc | A/T | Harbin | exonic | 2.93 |  |
|  |  |  |  |  |  | Liaoning |  | 4.41 |  |
|  |  |  |  |  |  | Jilin |  | 4.96 |  |
|  |  |  | 2676198 | Total-Toc | C/A | Harbin | downstream | 3.75 |  |
|  |  |  |  |  |  | Liaoning |  | 8.34 |  |
|  |  |  |  |  |  | Jilin |  | 7.75 |  |
|  |  |  | 2674177 | γ-Toc | T/C | Harbin | exonic | 2.10 |  |
|  |  |  |  |  |  | Liaoning |  | 2.32 |  |
|  |  |  |  |  |  | Jilin |  | 2.16 |  |
|  |  |  | 2674189 | γ-Toc | T/A | Harbin | intronic | 2.10 |  |
|  |  |  |  |  |  | Liaoning |  | 2.32 |  |
|  |  |  |  |  |  | Jilin |  | 2.16 |  |
|  |  |  | 2674397 | γ-Toc | A/T | Harbin | exonic | 1.48 |  |
|  |  |  |  |  |  | Liaoning |  | 2.83 |  |
|  |  |  |  |  |  | Jilin |  | 1.01 |  |
|  |  |  | 2676198 | γ-Toc | C/A | Harbin | downstream | 2.10 |  |
|  |  |  |  |  |  | Liaoning |  | 2.32 |  |
|  |  |  |  |  |  | Jilin |  | 2.16 |  |
| group-Ⅱ | *Glyma.10G171600* | 10 | 40517805 | Total-Toc | T/C | Harbin | downstream | 3.75 | RAB GTPase homolog A5A |
|  |  |  |  |  |  | Liaoning |  | 8.34 |  |
|  |  |  |  |  |  | Jilin |  | 7.75 |  |
|  |  |  | 40517815 | Total-Toc | T/C | Harbin | downstream | 2.64 |  |
|  |  |  |  |  |  | Liaoning |  | 2.64 |  |
|  |  |  |  |  |  | Jilin |  | 3.70 |  |
|  |  |  | 40517805 | γ-Toc | T/C | Harbin | downstream | 2.24 |  |
|  |  |  |  |  |  | Liaoning |  | 3.08 |  |
|  |  |  |  |  |  | Jilin |  | 2.29 |  |
|  |  |  | 40517815 | γ-Toc | T/C | Harbin | downstream | 2.05 |  |
|  |  |  |  |  |  | Liaoning |  | 2.74 |  |
|  |  |  |  |  |  | Jilin |  | 2.72 |  |
| group-Ⅱ | *Glyma.20G235100* | 20 | 46784932 | δ-Toc | A/C | Harbin | exonic | 3.43 | indeterminate(ID)-domain 2 |
|  |  |  |  |  |  | Liaoning |  | 4.08 |  |
|  |  |  |  |  |  | Jilin |  | 3.09 |  |
|  |  |  | 46786459 | δ-Toc | A/G | Harbin | downstream | 4.28 |  |
|  |  |  |  |  |  | Liaoning |  | 4.88 |  |
|  |  |  |  |  |  | Jilin |  | 3.90 |  |
|  |  |  | 46787012 | δ-Toc | T/C | Harbin | downstream | 4.28 |  |
|  |  |  |  |  |  | Liaoning |  | 4.88 |  |
|  |  |  |  |  |  | Jilin |  | 3.90 |  |
| group-Ⅱ | *Glyma.20G235400* | 20 | 46802178 | δ-Toc | A/C | Harbin | exonic | 3.50 | P-loop containing nucleoside triphosphate hydrolases superfamily protein |
|  |  |  |  |  |  | Liaoning |  | 1.54 |  |
|  |  |  |  |  |  | Jilin |  | 2.75 |  |
|  |  |  | 46802214 | δ-Toc | A/C | Harbin | exonic | 3.50 |  |
|  |  |  |  |  |  | Liaoning |  | 1.54 |  |
|  |  |  |  |  |  | Jilin |  | 2.75 |  |
|  |  |  | 46803240 | δ-Toc | C/G | Harbin | intronic | 1.51 |  |
|  |  |  |  |  |  | Liaoning |  | 2.54 |  |
|  |  |  |  |  |  | Jilin |  | 1.73 |  |
|  |  |  | 46803774 | δ-Toc | C/A | Harbin | exonic | 3.71 |  |
|  |  |  |  |  |  | Liaoning |  | 4.25 |  |
|  |  |  |  |  |  | Jilin |  | 4.48 |  |
| group-Ⅱ | *Glyma.20G235800* | 20 | 46825298 | δ-Toc | C/T | Harbin | exonic | 1.51 | Transducin/WD40 repeat-like superfamily protein |
|  |  |  |  |  |  | Liaoning |  | 2.56 |  |
|  |  |  |  |  |  | Jilin |  | 2.74 |  |
|  |  |  | 46825616 | δ-Toc | G/A | Harbin | intronic | 2.77 |  |
|  |  |  |  |  |  | Liaoning |  | 2.63 |  |
|  |  |  |  |  |  | Jilin |  | 2.07 |  |
|  |  |  | 46825944 | δ-Toc | A/T | Harbin | exonic | 2.77 |  |
|  |  |  |  |  |  | Liaoning |  | 2.63 |  |
|  |  |  |  |  |  | Jilin |  | 2.07 |  |
|  |  |  | 46826130 | δ-Toc | T/C | Harbin | exonic | 1.51 |  |
|  |  |  |  |  |  | Liaoning |  | 2.56 |  |
|  |  |  |  |  |  | Jilin |  | 2.74 |  |
|  |  |  | 46827063 | δ-Toc | G/A | Harbin | intronic | 3.94 |  |
|  |  |  |  |  |  | Liaoning |  | 4.02 |  |
|  |  |  |  |  |  | Jilin |  | 3.52 |  |
|  |  |  | 46827575 | δ-Toc | G/A | Harbin | exonic | 3.57 |  |
|  |  |  |  |  |  | Liaoning |  | 4.03 |  |
|  |  |  |  |  |  | Jilin |  | 3.13 |  |
